# Discovery of Novel Hepatitis C Virus NS5B Polymerase Inhibitors by Combining Random Forest, Multiple e-Pharmacophore Modeling and Docking

**DOI:** 10.1371/journal.pone.0148181

**Published:** 2016-02-04

**Authors:** Yu Wei, Jinlong Li, Jie Qing, Mingjie Huang, Ming Wu, Fenghua Gao, Dongmei Li, Zhangyong Hong, Lingbao Kong, Weiqiang Huang, Jianping Lin

**Affiliations:** 1 State Key Laboratory of Medicinal Chemical Biology, College of Pharmacy and Tianjin Key Laboratory of Molecular Drug Research, Nankai University, Tianjin, 300071, China; 2 High-Throughput Molecular Drug Discovery Center, Tianjin Joint Academy of Biomedicine and Technology, Tianjin, 300457, China; 3 PracticaChem-China, Tianjin, 300192, PR China; 4 Tsinghua-Peking Center for Life Sciences, School of Life Sciences, Tsinghua University, Beijing, 100084, China; 5 College of Life Sciences, Nankai University, Tianjin, 300071, China; 6 College of Bioscience and Engineering, Jiangxi Agricultural University, Nanchang, 330045, China; Institute of Molecular Genetics IMG-CNR, ITALY

## Abstract

The NS5B polymerase is one of the most attractive targets for developing new drugs to block Hepatitis C virus (HCV) infection. We describe the discovery of novel potent HCV NS5B polymerase inhibitors by employing a virtual screening (VS) approach, which is based on random forest (RB-VS), e-pharmacophore (PB-VS), and docking (DB-VS) methods. In the RB-VS stage, after feature selection, a model with 16 descriptors was used. In the PB-VS stage, six energy-based pharmacophore (e-pharmacophore) models from different crystal structures of the NS5B polymerase with ligands binding at the palm I, thumb I and thumb II regions were used. In the DB-VS stage, the Glide SP and XP docking protocols with default parameters were employed. In the virtual screening approach, the RB-VS, PB-VS and DB-VS methods were applied in increasing order of complexity to screen the InterBioScreen database. From the final hits, we selected 5 compounds for further anti-HCV activity and cellular cytotoxicity assay. All 5 compounds were found to inhibit NS5B polymerase with IC_50_ values of 2.01–23.84 μM and displayed anti-HCV activities with EC_50_ values ranging from 1.61 to 21.88 μM, and all compounds displayed no cellular cytotoxicity (CC_50_ > 100 μM) except compound N2, which displayed weak cytotoxicity with a CC_50_ value of 51.3 μM. The hit compound N2 had the best antiviral activity against HCV, with a selective index of 32.1. The 5 hit compounds with new scaffolds could potentially serve as NS5B polymerase inhibitors through further optimization and development.

## Introduction

Chronic hepatitis C virus (HCV) infection has become a major global health problem because it can result in chronic liver disease, progressing to cirrhosis and hepatocellular carcinoma [1]. There are approximately 130 to 150 million people infected with HCV globally, of whom 350,000 to 500,000 die each year from HCV-related liver diseases [2]. Currently, the therapy for treating HCV infection includes taking ribavirin daily in addition to one of the two antiviral medications, sofosbuvir or simeprevir, and, in many instances, injection of pegylated α-interferon (PEG-IFN) [3]. However, the current HCV therapies are accompanied by numerous side effects and drug resistances [4]. Therefore, it is urgent to develop additional new anti-HCV drugs.

HCV NS5B polymerase, an RNA-dependent RNA polymerase, is responsible for the replication of positive-strand genomic RNA of HCV [5,6]. NS5B polymerase is considered an attractive target for therapeutic intervention of HCV-related diseases due to its unique and distinctive ability to utilize the RNA template that the host mammalian cell lacks [7]. NS5B polymerase is a 66 kDa protein of ~590 amino acids located at the C-terminus of the 3000-amino-acid polyprotein encoded by the HCV virus [8]. Similar to other known polymerases, the three-dimensional structure of NS5B comprises a right-hand topology with the characteristic finger, palm, and thumb regions [9,10]. The active site of the polymerase is located in the palm region [11]. Recently, several X-ray structures of the inhibitor-bound HCV NS5B polymerase or free enzyme have been solved [9,11], which provide help for the discovery and development of new structure-based NS5B polymerase inhibitors.

In the past decade, many NS5B polymerase inhibitors have been reported, and they can be classified into non-nucleoside inhibitors (NNIs) and nucleoside or nucleotide inhibitors (NIs) based on their mode of action [12]. NIs act as substrate mimics for the polymerase, preventing the replication elongation of the RNA chain by competing with the natural nucleoside triphosphate, while NNIs bind to the allosteric sites on the thumb or palm region of NS5B, inducing the conformation change of the NS5B polymerase that is needed for initiation of RNA synthesis and blocking of the enzyme activity [13]. The known NS5B polymerase inhibitors are reported as offering an excellent foundation for the discovery of new inhibitors.

Numerous *in silico* methods have been applied to discover and develop new NS5B polymerase inhibitors [14–33], including quantitative structure-activity relationships (QSAR), pharmacophore modeling, molecular dynamics (MD) simulation and molecular docking. Barreca et al. reviewed conventional computational methods and the recent development of in silico approaches aimed at identification and optimization of non-nucleoside inhibitors binding to allosteric sites of the HCV NS5B polymerase [13]. Talele et al. employed a step-wise approach for the virtual screening, including database filtration and succeeding high-throughput docking against thumb site I, and identified two novel chemotypes displaying good NS5B inhibitory activity [17]. A funnel approach was employed to develop potential thumb site II inhibitors by Corbeil et al. in 2008, and two druglike compounds were identified from maybridge library [24]. Musmuca et al employed ligand based and structure based alignments for 3D-QSAR studies to identify four new thumb site II inhibitors with IC_50_ values ranging between 46 and 73.3 μM [25]. Recently, Therese et al. have employed multiple e-pharmacophore modeling, 3D-QSAR models and high-throughput virtual screening to discover two new NNIs with IC_50_ values ranging between 28.8 and 47.3 μM targeting NS5B polymerase [34]. Computational strategies have been proven to be a powerful and available tool for the identification of new chemotypes as NS5B polymerase NNIs.

**Fig 1:**
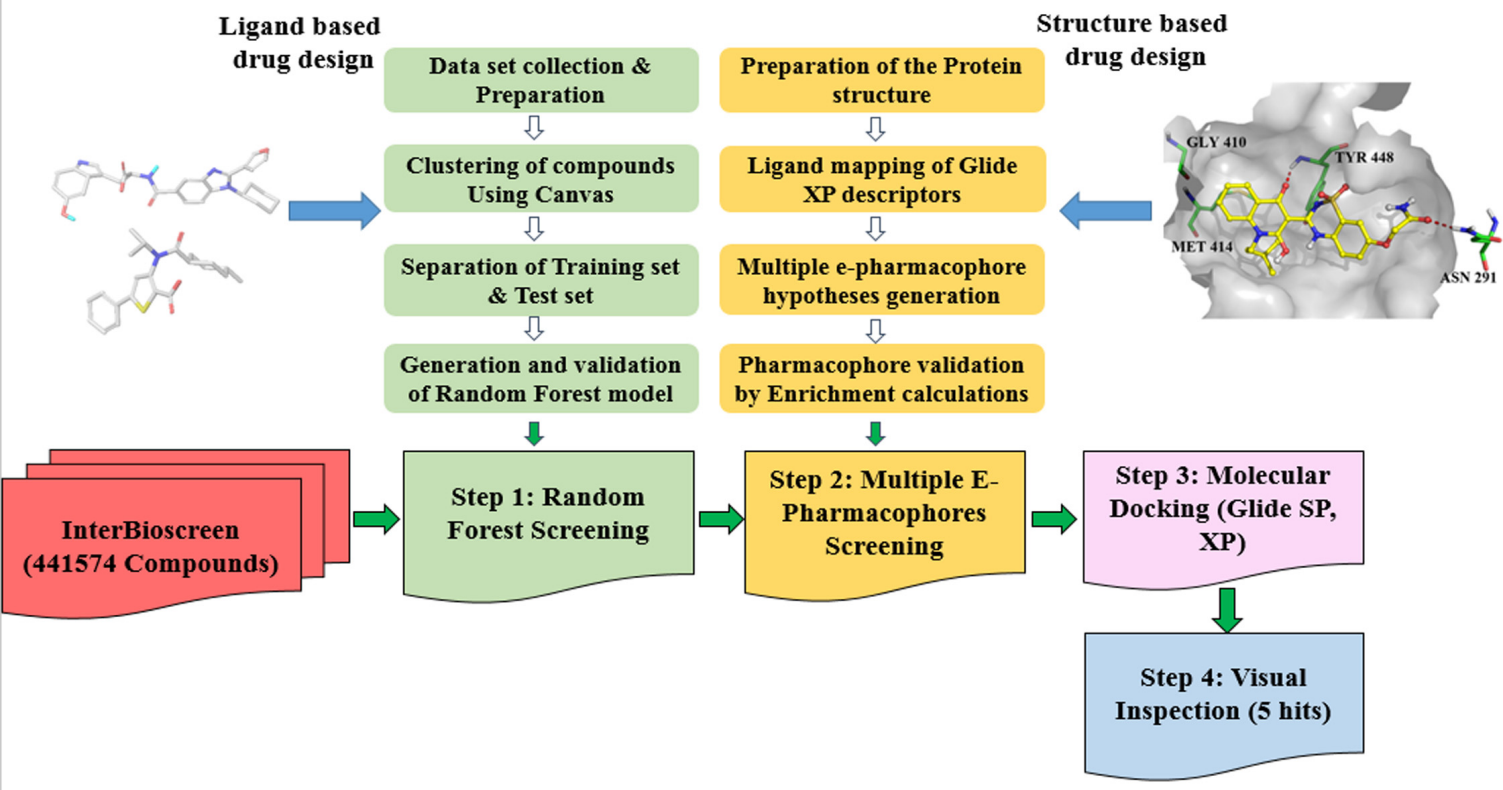
A chart for the virtual screen targeting HCV NS5B polymerase.

In the present study, we discovered a series of novel small molecule NS5B polymerase inhibitor leads using a virtual screening workflow that includes random forest (RB-VS), e-pharmacophore (PB-VS), and molecular docking (DB-VS) methods. The virtual screening workflow is depicted in Fig 1. First, the random forest (RF) method was used to build the predictive models of the NS5B polymerase inhibitors. 16 descriptors were selected, and the overall classification accuracy of the constructed Model III was 84.4%. Then, six co-crystal structures of NS5B polymerase with inhibitors binding at the palm I, thumb I and thumb II regions were used to build the multiple e-pharmacophore models [13]. Third, Glide SP and XP docking protocols were utilized in the DB-VS stage. The three virtual screening methods were applied in a hierarchical fashion that the fastest filter RB-VS was first applied, and the second fast filter PB-VS was subsequently applied, and the slowest filter DB-VS was finally applied. A chemical library, including 441,574 compounds from the InterBioScreen database, was screened with the above virtual screening approach. We selected 5 compounds from the final hits for further anti-HCV and cellular cytotoxicity assay. All 5 compounds showed inhibitory potency against NS5B polymerase with IC_50_ value of 2.01–23.84 μM, and displayed certain inhibitory activities against HCV and no (or weak) cellular cytotoxicity. These compounds can be further optimized and developed into potent and highly active NS5B polymerase inhibitors.

## Materials and Methods

### Data sets and database preparation

A total of 1029 compounds (see S1–S3 Tables in supporting information), including 579 positives (NS5B inhibitors, IC_50_ ≤ 400 nM) and 450 negatives (NS5B noninhibitors, IC_50_ ≥ 600 nM) [35], were selected from the literature, Binding DB and CHEMBL database [36–49]. The 1029 compounds were first divided into different clusters on the basis of their scaffolds. Then, compounds were selected from each cluster randomly but approximately proportional to construct the training and test sets for the generation and validation of the RF models and the e-pharmacophore models[50]. The training set for the RF models contains 389 positives (IC_50_ ≤ 400 nM) and 383 negatives (IC_50_ ≥ 600 nM) [51]. The test set for the RF models comprises 74 positives (IC_50_ ≤ 400 nM) and 67 negatives (IC_50_ ≥ 600 nM). The remaining 116 positive (IC_50_ ≤ 400 nM) compounds (test set for palm I: 63 positives; test set for thumb I: 36 positives; test set for thumb II: 17 positives), together with the 1000 decoys (the “dl-400” data set) obtained from Schrödinger [52], were used to evaluate the performances of the e-pharmacophore models. To evaluate the performance of the multistage VS approach, an additional validation set consisting of the 73 positives (IC_50_ ≤ 400 nM) with 2190 decoys molecules (30 decoys from DecoyFinder [53] for each active) were screened using RB-VS, PB-VS and DB-VS in different consequential orders.

Data set and chemical database preparation was performed by the LigPrep [54] with Epik [55] to explore protonation and tautomeric states at 7.0 ± 2.0 pH units. The ConfGen [56] conformational sampling algorithm with the OPLS_2005 force field [57]was used to generate conformers for the compounds. Redundant conformers were removed using a duplicate pose elimination criterion of 1.0 Å RMSD (root-mean-square deviation)[34]. Electrostatic interactions were treated with a distance-dependent dielectric solvation.

### Random forest modeling

The initial descriptors used in this study were calculated with Dragon 6.0 [58]. The “*randomForest*” package in ***R*** [59] was employed to construct random forest models for classification of HCV NS5B polymerase inhibitors and noninhibitors. RF constructed a multitude of decision trees and used the ensemble learning method for classification of the samples. Approximately two-thirds of the data set were used to build a classification tree. Approximately one-third of the data were left, called Out Of Bag (OOB) data. OOB data that gives an internal validation of RF was utilized to estimate the prediction accuracy of the RF model. The overall accuracy of the entire forest is measured by the average of the error rates for all decision trees. The Mean Decrease in Accuracy Decrement importance measure was used to choose important variables during the process of constructing classification trees. The number of decision trees was designated to 1000. The default values of the ***R*** software were designed for the other parameters.

### E-pharmacophore generation and validation

To improve the potency of identifying active and novel hits from the in silico screening, we used a total of six co-crystal structures of HCV NS5B polymerase (PDB ID: 3HHK, 3SKA, 2BRK, 4DRU, 2GIR and 3PHE) [47,60–64] bound with inhibitors to build e-pharmacophore models. To discover high-affinity ligands, we built the pharmacophore models based on a series of 6 structurally diverse chemicals exhibiting IC_50_ or K_d_ values from 2.4 nM to 140 nM for all three regions of NS5B polymerase. The co-crystal ligand structures and the resolution and affinity values are listed in S1 Fig (see supporting information). The protein preparation wizard in Schrödinger software [65] was used to process the protein structures. All water molecules in structures were removed [14,25,34,66,67], and the resulting structures were prepared by assigning bond order, adding hydrogen atoms, treating disulfides, and assigning protonation states, as well as by performing minimization with OPLS_2005 force field by converging the heavy atoms to an RMSD of 0.30 Å [57].

Glide energy grids were set up for all six prepared protein structures using the Receptor Grid Generation panel in Maestro. The crystal ligands were redocked using the “Refine” option in Glide [68], and the “write XP descriptor information” option was chosen. The optimization and scoring were performed using default settings. On the basis of the XP descriptors information, pharmacophore sites were automatically generated with Phase [69] using the default set of six chemical features: hydrogen-bond acceptor (A), hydrogen-bond donor (D), hydrophobe (H), negative ionizable (N), positive ionizable (P), and aromatic ring (R). Initially, the number of pharmacophore sites was designed to 10 for all the crystal structures. The energetic value assigned to each pharmacophore feature site was equal to the sum of the Glide XP energies from the atoms comprising the site. Then, each pharmacophore feature site was then quantified and ranked based on its energetic value [70,71].

The ability to reproduce known inhibitors of the e-pharmacophore hypotheses was evaluated by the three test sets, respectively. Enrichment Factor (EF) was employed for describing the number of known inhibitors recovered when the database is screened. In this study, we focused primarily on EF(1%), which refers to the enrichment in the top 1% of the decoys [72]. The Boltzmann-enhanced discrimination of the receiver operating characteristic (BEDROC) metric was used to measure the early recognition of hits in an ordered list [73]. α = 20.0 and α = 160.9 were all used for the comparison. The value of α = 20.0, which corresponds to 80% of the score being accounted for in the top 8% of the database, was suggested as a reasonable choice for virtual screening evaluations [73].

### Molecular docking

The docking algorithm Glide was used to perform all the molecular docking studies [68]. The six co-crystal structures (PDB ID: 3HHK, 3SKA, 2BRK, 4DRU, 2GIR and 3PHE) [47,60–64] were prepared and then used to build the energy grid. The grids were generated at the centroid of the co-crystallized ligands. Default settings were employed for both the grid generations and docking. Post-minimization was used to optimize the geometry of the poses.

### Virtual screening

We constructed a virtual screening approach by combining the RF-based virtual screening (RB-VS), the e-pharmacophore-based virtual screening (PB-VS) and the docking-based virtual screening (DB-VS) methods. In this investigation, we applied the three virtual screening methods in increasing order of complexity. In the RB-VS stage, a chemical library, including 441,574 compounds from the InterBioScreen database, was screened. The compounds that passed through the RB-VS filter then were processed by a second filtering of PB-VS. In the PB-VS stage, screening molecules were required to match each site in the hypothesis. The distance matching tolerance was designated to 2.0 Å. A fitness score was used to rank the database hits based on their RMSD with the hypothesis involving site matching, vector alignments and volume terms [74]. In the DB-VS stage, the compounds that passed through the PB-VS filter were further screened using docking methods.

### Anti-replicon activity and cytotoxicity assays

#### HCV virus and replicon cell lines

The HCV virus assay was constructed by using the method developed as previously described, albeit with a slight modification [75–77]. Briefly, the plasmid of pRluc-JFH-1 was constructed as following. Based on the plasmid of pJFH-1, as a gift from Apath,L.L.C., a humanized Renilla luciferase reporter gene was introduced into the C-terminus of NS5A in the JFH-1 genome. The plasmid phRluc-JFH-1 was made via digestion with the XbaI restriction enzyme and used as a template for RNA transcription. The virus transcripts were prepared in vitro by using the Ambion MEGAscript Kits, and then 10 μg of RNA was mixed with 400 ml of Huh7.5.1, which was gifted by Jin Zhong (Institute Pasteur of Shanghai, Chinese Academy of Science), at a concentration of 1×10^7^ cells/ml. After electroporation, the Huh7.5.1 cells containing virus transcripts were seeded in a 10-cm dish. After the cells were cultured for 4 days, the supernatant was collected and filtered to obtain the stock solution of the hRluc-JFH-1 virus. To obtain the virus titer, the virus stocks were diluted at a gradient of 1:10, and the Huh7.5.1 cells were incubated for 48h at 37°C. Then, the cells were harvested, and the luminescence was detected via the manufacturer’s protocol of the Renilla-Glo™ Luciferase Assay System (Promega).

#### Viral inhibition assay

Huh7.5.1 cells were seeded in 96-well plates at a density of 2 ×10^4^ cells per well at 37°C overnight. All of the synthetic compounds were diluted with DMSO to 10 mM of stock solution. The initial concentration of compounds was 20 μM and then diluted at a gradient of 1:4 to concentrations ranging from 20 μM to 0.02 μM, containing 0.5% DMSO. For the HCVcc system, serial diluted compounds were mixed with a certain titer of HCVcc-hRluc-JFH1 virus, and the final concentration of HCVcc-hRluc-JFH1 virus titer was diluted to the numbers of relative luminescence units (RLU) ranging from 20,000 to 50,000 RLU and then added to the Huh7.5.1 cells. Then, the cells were cultured for 2 days at 37°Cand harvested. The luminescence was detected via the manufacturer’s protocol of the Renilla-Glo™ Luciferase Assay System. EC_50_ is the concentration of the compound at which the HCV luminescence level in the Huh7.5.1 cells is reduced by 50%. The values of EC_50_ were plotted by the GraphPad Prism 5 software.

#### Cell proliferation assay

Huh7.5.1 cells were seeded in 96-well plates at a density of 2 × 10^4^ cells per well overnight. Cells were incubated with serial diluted compounds for 48 h. The viability of Huh7.5.1 cells was determined in 96-well tissue culture plates using the cell proliferation reagent WST-1 (Roche), and absorbance (OD450 / reference OD630) was measured to detect the cytotoxicity of compounds according to the manufacturer’s protocol for the Cell Proliferation Reagent WST-1. CC_50_ is the concentration of the compound at which the cell growth was inhibited by 50%. The values of CC_50_ were plotted by the GraphPad Prism 5 software.

### SPR interaction analysis

The SPR experiments were performed using a Biacore T200 optical biosensor (Biacore Life Sciences, GE Healthcare). The NS5B protein at 50 μg/ml prepared in buffer of 10 mM sodium acetate (pH 5.5) was immobilized by standard amine coupling to a CM5 biosensor chip. The compounds were injected for 60 s at a flow rate of 30 μL/min using in concentration series between 1 and 250 μM in running buffer (1 × PBS with 5% DMSO). The dissociation was monitored for 300 s. Raw data collected on an SPR biosensor were further processed to eliminate any artifacts such as nonspecific binding and discrepancies in buffer composition. All data processing and analysis was performed using the Biacore T200 Evaluation Software.

### NS5B polymerase inhibition assay

The inhibitory effects of the tested reagents on in vitro NS5B activity were determined by NS5B-catalyzed RNA synthesis assay as described previously [78]. Purified HCV NS5B protein and HCV (-) 3’ T RNA template were obtained as described previously [79]. The tested reagents were added at a variety of concentrations in 50 μl reaction mixture consisting of 20 mM HEPES (pH 8.0), 1.5 mM MnCl_2_, 100 mM ammonium acetate, 1 mM DTT, 500 μM GTP, 250 μM each of CTP, ATP and UTP, 40U of RNasin (Biostar, Canada), 2 μg/ml HCV (-) 3’ TRNA template and 300 ng purified NS5B protein. After 2 h at 30°C, the reaction was stopped by 100 mM EDTA and RNA was purified using the RNaid Kit (Qbiogene). To determine NS5B-catalyzed RNA synthesis, real-time RT-PCR was performed. cDNA was synthesized using AMV-reverse transcriptase (TAKARA, Japan) and the primer targeted to NS5B-catalyzed RNA product (5’-caagcaccctatcaggcagt-3’) and then digested with RNAase. PCR was carried out using RNA product–specific primers (forward, gaaaggctgggtcctttctt, reverse 5’-caagcaccctatcaggcagt-3’, probe, 5’-ctagcctagtagcgttgggttgcgaac-3’) and TaqMan RT-PCR Kit according to the instruction. 50% inhibitory concentrations (IC_50_) were calculated using Statistical SPSS program version 11.5 by probit analysis of regression.

## Results and Discussion

### Establishment and validation of the RF model

The training set comprises 772 compounds, including 389 known NS5B polymerase inhibitors and 383 putative noninhibitors. Initially, 4882 molecular descriptors were generated with Dragon 6.0. The initial descriptors were pretreated to discard the “bad” descriptors: (1) descriptors with too many zero values were removed; (2) descriptors with very small standard deviation values (< 0.5%) were removed; and (3) descriptors that are highly correlated with others (correlation coefficients > 95%) were removed[50]. A total of 577 molecular descriptors were left after preprocessing. Then, the 577 descriptors were further filtered using the RF method.

In the first stage, a full RF model (Model I) was built using all 577 available descriptors. To drop unimportant variables from an RF, the Mean Decrease in Accuracy Decrement importance measure was used. By dropping the less important descriptors (Mean Decrease in Accuracy < 5), a total of 43 descriptors were obtained in the second stage. The 43 descriptors were used to build a second RF model (Model II). In the third stage, the dropping process was repeated until only a few descriptors (with a Mean Decrease in Accuracy > 6) remained. At this point, 16 descriptors were finally chosen to build the third RF model (Model III). The 16 descriptors can be roughly divided into several groups: Walk and path counts (1); Topological indices (1); RDF descriptors (1); GETAWAY descriptors (3); Edge adjacency indices (6); CATS 2D (2); Atom-type E-state indices (1) and 2D autocorrelations (1) (see S4 Table in supporting information).

Subsequently, the three established models were validated with an independent test set (74 inhibitors and 67 noninhibitors). To evaluate and compare the different RF models, the sensitivity (SE), specificity (SP) and overall accuracy (Q) were used as the performance criterions. Table 1 lists the values of SE, SP, and Q for the three RF models. Model I showed an SE of 77.0%, an SP of 83.6% and a Q of 80.2%. Model II showed an SE of 78.4%, an SP of 83.6% and a Q of 80.9%. Model III showed an SE of 81.1%, an SP of 88.1% and a Q of 84.4%. The higher values of SE and SP for Model III indicate that the prediction accuracies for inhibitors and noninhibitors are higher than those of Model I and II. The higher value of Q indicates that the overall prediction accuracy of Model III is better than those of Model I and II [80]. In the paper of Svetnik et al [59], when they built random forest models using different number of descriptors (based on variable importance measure method) from 1522 to 3, the performances of random forest were roughly constant until the number of descriptors is reduced to about 191, after which the performance begins to degrade, which is consistent with the results from our model I, II, III. Therefore, the simpler Model III, with only 16 descriptors, is better than Model I and Model II with respect to the values of SP, SE and Q. RF Model III was adopted for further virtual screening of HCV NS5B polymerase inhibitors.

**Table 1:** Results of RF model validation by an independent test set.

model	no. of descriptors	TP^*a*^	FN^*b*^	TN^*c*^	FP^*d*^	SE (%)^*e*^	SP (%)^*f*^	Q (%)^*g*^
I	577	57	17	56	11	77.0	83.6	80.2
II	43	58	16	56	11	78.4	83.6	80.9
III	16	60	14	59	8	81.1	88.1	84.4

^*a*^ TP: true positive.

^*b*^ FN: false negative.

^*c*^ TN: true negative.

^*d*^ FP: false positive.

^*e*^ SE (%): sensitivity, SE = TP/ (TP + FN).

^*f*^ SP (%): specificity, SP = TN/ (TN + FP).

^*g*^ Q (%): overall accuracy, Q = (TP + TN)/ (TP + FP + TN + FN).

To evaluate the effect of activity cutoffs on random forest accuracy and performance, we constructed three datasets: (i) Set-150: positives ≤ 150 nM, negatives > 150 nM, (ii) Set-400: positives ≤ 400 nM, negatives ≥ 600 nM, (iii) Set-950: positives ≤ 950 nM, negatives > 950 nM [81]. The results (see S5 Table) indicated that Set-400 generated better RF model than Set-150 and Set-950 (see supporting information for detailed discussion). The influence of the selected training data randomly and by scaffold on the prediction precision was also analyzed [50,82–84]. The results (S6 Table of supporting information) showed that models based on scaffold method generated better statistical results for the test sets than models based on random division (see supporting information for detailed discussion).

Overfitting is always a weak point limiting the application of machine learning approach [85]. In order to explore whether a RF model is robust to overfitting, we compare the error rates of out-of-bag, training set and independent test set as increase the number of trees [59]. The test and OOB error rates converged to their “asymptotic” values, which is close to their minimum, rather than increasing after the training error reaches zero. When the number of trees is sufficiently large, the OOB error rate correlates with the test error rate quite well. This result demonstrates that there is no over-fitting in our model. An intuitive comparison of error rates is provided in S2 Fig.

### E-pharmacophore modeling and model validation

**Fig 2:**
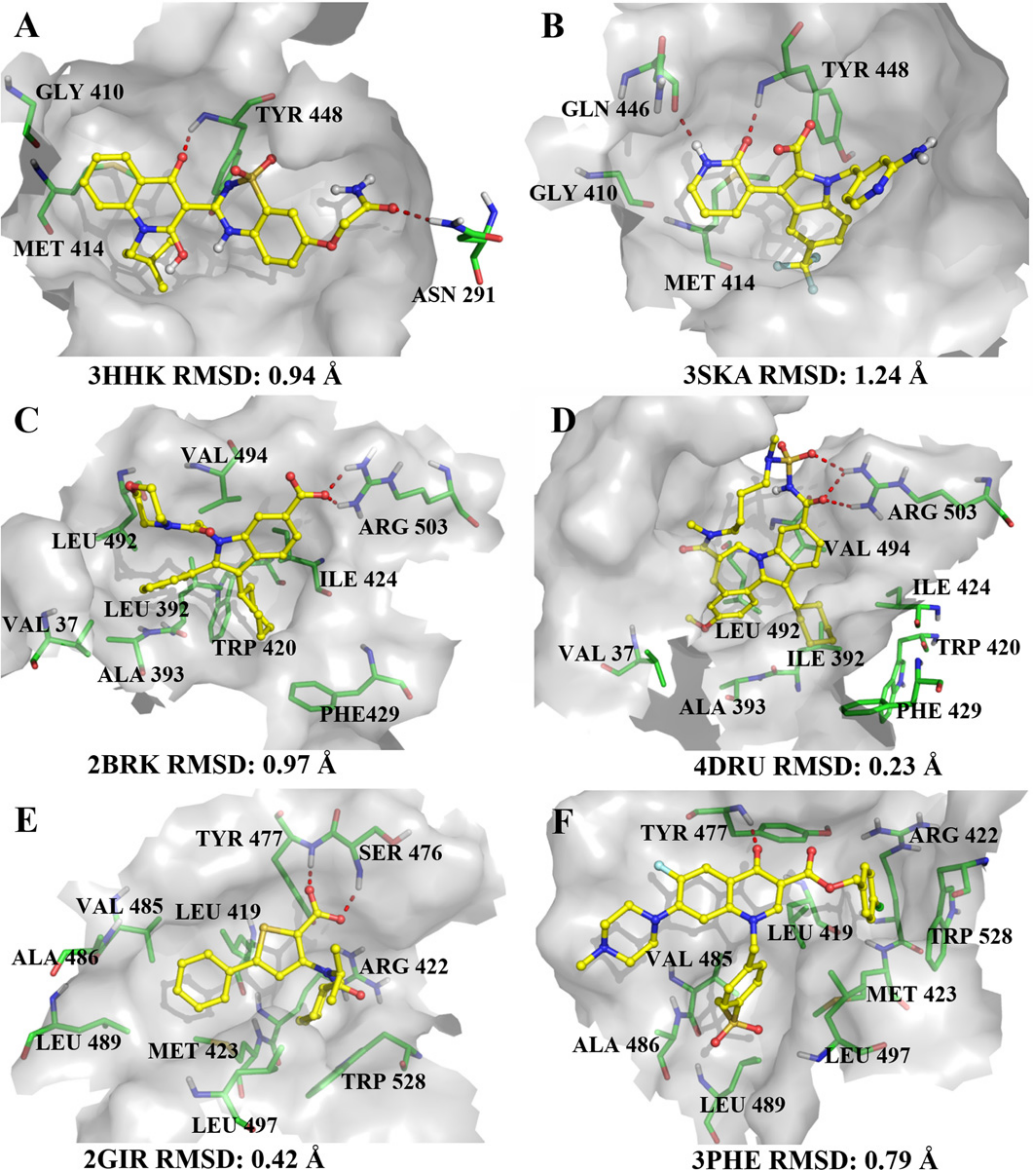
Redocked binding modes of the co-crystalized inhibitors in the active site of NS5B polymerase. (A-B) the palm I region, (C-D) the thumb I region, (E-F) the thumb II region.

Of the six NS5B polymerase crystal structures, inhibitors of 3HHK and 3SKA bound to the palm I region, inhibitors of 2BRK and 4DRU bound to the thumb I region, and inhibitors of 2GIR and 3PHE bound to the thumb II region. The six crystal ligands were redocked in the active sites of NS5B to generate e-pharmacophore, respectively. All the redocked structures showed low RMSD values between the docked conformations of ligands and their crystal structures’ binding poses. All the RMSD values were less than 1.0 Å except 3SKA (see Fig 2). Fig 2 shows that the important residues in the palm I region were Asn291, Gln446 and Tyr448, the important residue in the thumb I region was Arg503, and the important residues in the thumb II region were Ser476 and Tyr477. These important residues are consistent with the original crystal structures [8,86,87].

**Fig 3:**
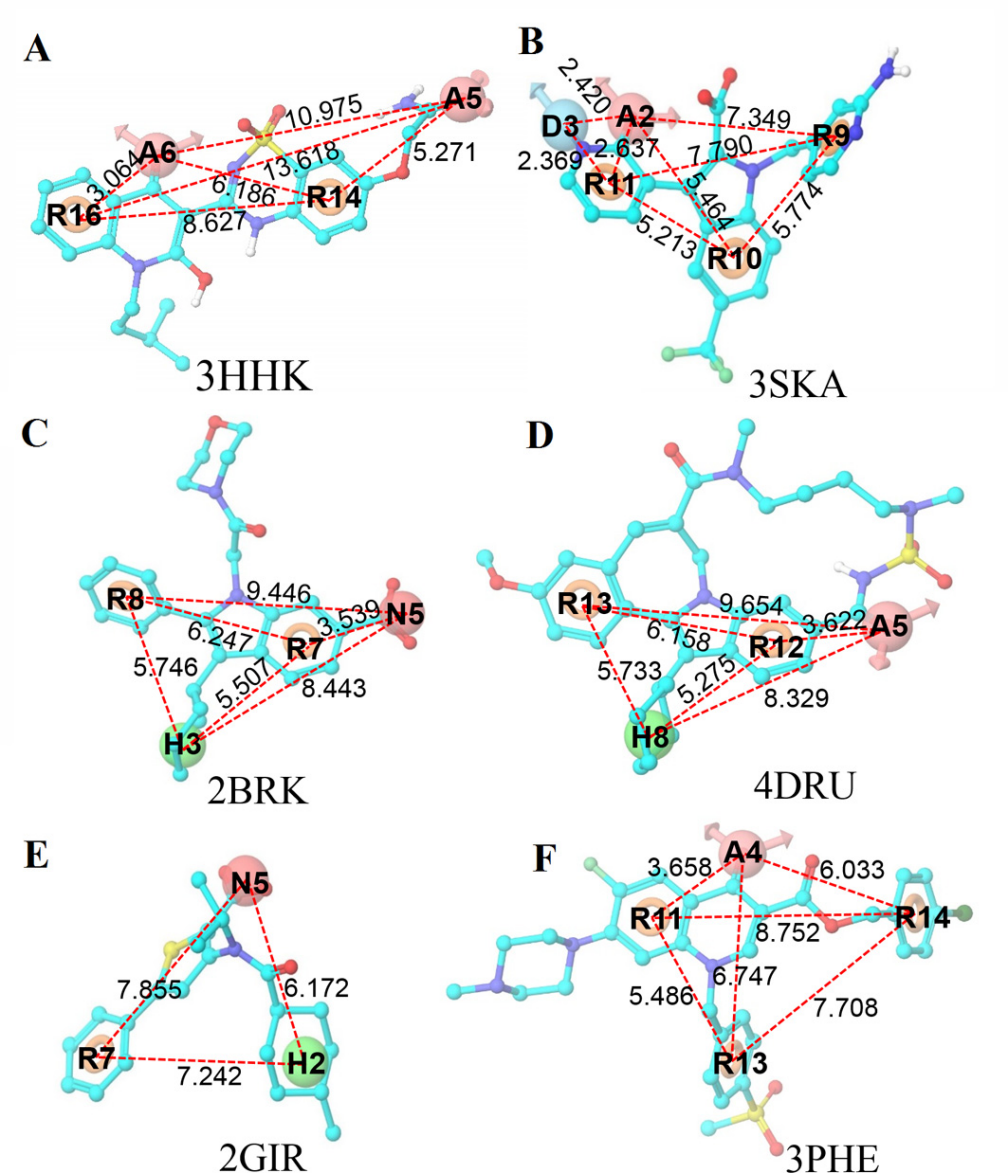
E-pharmacophore hypotheses with energetically favorable sites from the six crystal structures. Pink sphere represents hydrogen-bond acceptor (A); orange ring represents aromatic ring (R); blue sphere represents hydrogen-bond donor (D); red sphere represents negatively ionizable (N); green spheres represent hydrophobic (H).

The e-pharmacophore method incorporating the aspects of structure-based and ligand-based techniques was used to explore the six crystal structures of the NS5B polymerase. This technique not only helps us eliminate the pharmacophore sites that lack significant interactions but also prioritize the sites during virtual screening. Glide XP energetic terms were mapped onto pharmacophore sites to generate pharmacophore hypotheses. These pharmacophore sites were calculated based on the energy and structural information of protein complex. Table 2 lists the number of pharmacophore sites for each ligand prior to energy-based site selection, the number of selected sites, the e-pharmacophore hypotheses and the scores for each feature in the hypotheses. Therefore, the pharmacophoric features for the palm I region were A5A6R14R16 (3HHK) and A2D3R9R10R11 (3SKA), the pharmacophoric features for the thumb I region were N5H3R7R8 (2BRK) and A5H8R12R13 (4DRU), and the pharmacophoric features for the thumb II region were N5H2R7 (2GIR) and A4R11R13R14 (3PHE) (see Fig 3). The sites show high scores as the ligand atoms mapping to them exhibited promising interaction energy with the amino acids in the binding pocket. The general pharmacophoric sites of the palm I region were acceptor (A) and ring (R). The important sites obtained in the e-pharmacophore, such as A6 (in 3HHK) and A2 (in 3SKA), correspond to the important hydrogen bond in the backbone amino group of Tyr448, which can be observed clearly from Fig 2A and 2B. The two ring sites R16 (in 3HHK) and R11 (in 3SKA) occupy a hydrophobic pocket mainly defined by the residues Met414 and Gly410. Hydrophobic group (H) and ring (R) features were common for the thumb I region. The two hydrophobic sites H3 (in 2BRK) and H8 (in 4DRU) were well placed in the hydrophobic pocket formed by Leu392, Trp420, Ile424, and Phe429 (see Fig 2C and 2D). R8 (in 2BRK) and R13 (in 4DRU) point towards the hydrophobic pocket formed by the residues Val37, Ala393, Leu492 and Val494. N5 (in 2BRK) and A5 (in 4DRU) form two hydrogen bonds with the guanidine of Arg503 (see Fig 2C and 2D). In the thumb II region, the ring (R) feature was common, and N5 (in 2GIR) and A4 (in 3PHE) form hydrogen bonds with the backbone amino groups of Ser476 and Tyr477. H2 (in 2GIR) and R14 (in 3PHE) occupy a hydrophobic pocket formed by Leu419, Arg422, Met423, and Trp528. R7 (in 2GIR) and R13 (in 3PHE) occupy a second shallow hydrophobic pocket formed by Leu419, Val485, Ala486, Leu489, Leu497, and Met423 (see Fig 2E and 2F).

**Table 2:** E-pharmacophore features and the scores for each feature in e-pharmacophore hypotheses generated from the six crystal structures.

PDB code	no. of possible sites	no. of selected sites	hypothesis	feature score
3HHK	6	4	A^*a*^5A6R^*b*^14R16	A5: -0.71; A6: -0.64; R14: -0.58; R16: -0.88
3SKA	7	5	A2D^*c*^3R9R10R11	A2: -0.63; D3: -0.63; R9: -0.69; R10: -0.83; R11: -0.77
2BRK	4	4	N^*d*^5H^*e*^3R7R8	N5: -3.43; H3: -0.51; R7: -0.74; R8: -1.10
4DRU	5	4	A5H8R12R13	A5: -0.18; H8: -0.56; R12: -0.59; R13: -0.81
2GIR	4	3	N5H2R7	N5: -0.70; H2: -0.19; R7: -0.98
3PHE	5	4	A4R11R13R14	A4: -0.70; R11: -0.69; R13: -0.63; R14: -1.21

^*a*^ A: acceptor.

^*b*^ R: ring aromatic.

^*c*^ D: donor.

^*d*^ N: negatively ionizable.

^*e*^ H: hydrophobic.

Fig 3 presents the distance mapping among the features in each e-pharmacophore hypothesis. Regarding the distance among different features when comparing two ligands from the same active region, for the palm I region, the distances among A6, R14 and R16 in the hypothesis A5A6R14R16 (3HHK) are similar to the distances among A2, R9 and R11 in the hypothesis A2D3R9R10R11 (3SKA) (see Fig 3A and 3B). For the thumb I region, the distances among N5, R8, R7 and H13 in the hypothesis N5H3R7R8 (2BRK) are similar to the distances among A5, R12, R13 and H8 in the hypothesis A5H8R12R13 (4DRU) (see Fig 3C and 3D). For the thumb II region, the distances among N5, H2 and R7 in the hypothesis N5H2R7 (2GIR) are similar to the distances among A4, R14 and R13 in the hypothesis A4R11R13R14 (3PHE) (see Fig 3E and 3F).

To explore the performance of e-pharmacophore hypotheses, three test sets were employed to evaluate whether the e-pharmacophore models have the ability to differentiate between NS5B polymerase inhibitors and noninhibitors. The test set for the palm I region includes 63 known inhibitors and 1000 decoys, the test set for the thumb I region includes 36 known inhibitors and 1000 decoys, and the test set for the thumb II region includes 17 known inhibitors and 1000 decoys. The enrichment results of all six e-pharmacophore hypotheses were compared for the enrichment factor (EF1%) and BEDROC (α = 160.9 and α = 20.0), based on the recovery rate of inhibitors against the respective ranked test set (see Table 3). For the palm I region, the 3HHK pharmacophore showed better EF1% (12) and BEDROC values (α-160.9 = 0.76 and α-20 = 0.52) than those of the 3SKA pharmacophore (EF1% = 6, α-160.9 = 0.37 and α-20 = 0.15). For the thumb I region, the 4DRU pharmacophore and 2BRK pharmacophore showed similar performance, with similar EF1% and BEDROC values. For the thumb II region, the 2GIR pharmacophore showed higher EF1% (54) and BEDROC values (α-160.9 = 0.82 and α-20 = 0.57) than those of the 3PHE pharmacophore (EF1% = 12, α-160.9 = 0.24 and α-20 = 0.14). The enrichment results for all of the pharmacophoric hypotheses were compared based on enrichment factor (EF) and BEDROC (α = 160.9, a = 20.0) as shown in S7–S11 Tables. Among these hypotheses, pharmacophore model A5A6R14R16 for 3HHK, A2D3R9R10R11 for 3SKA, N5H3R7R8 for 2BRK, A5H8R12R13 for 4DRU, N5H2R7 for 2GIR and A4R11R13R14 for 3PHE show the highest EF1% and BEDROC values, which were selected for further screening.

**Table 3:** Validation of the six e-pharmacophore hypotheses.

PDB code	Hypothesis	EF1%^*a*^	RIE^*b*^	ROC^*c*^	BEDROC (α-160.9) ^*d*^	BEDROC (α-20)
3HHK	A5A6R14R16	12	6.08	1.02	0.76	0.52
3SKA	A2D3R9R10R11	6	1.75	1.03	0.37	0.15
2BRK	N5H3R7R8	29	9.18	1.01	0.93	0.64
4DRU	A5H8R12R13	29	10.83	1.01	0.97	0.75
2GIR	N5H2R7	12	4.20	0.97	0.29	0.25
3PHE	A4R11R13R14	12	2.36	0.99	0.24	0.14

^*a*^ EF1%: enrichment factor at 1% of the decoy data set.

^*b*^ RIE: robust initial enhancement.

^*c*^ ROC: receiver operating characteristic curve value.

^*d*^ BEDROC: boltzmann-enhanced discrimination of receiver operating characteristic.[34]

The EF1% and BEDROC values of the six pharmacophore models indicate that the pharmacophore models generated from the six co-crystal structures of the NS5B polymerase could effectively identify the inhibitors in the entire ranked decoy database. Moreover, pharmacophore models developed from the same protein target based on different regions and different ligands are important to identify diverse hits from the database screening. Therefore, the six pharmacophore models were all subjected to the following virtual screening.

### Determining docking protocols

To determine the docking protocol, the six co-crystal ligands that were retrieved from the palm I (3HHK and 3SKA), thumb I (2BRK and 4DRU) and thumb II (2GIR and 3PHE) regions were docked to their corresponding active sites of the NS5B polymerase. In 2014, Barreca et al [8] pointed out that several factors including target identity and conformation, and ligand chemotype would have a highly impact on the effect of water molecules in docking-based experiments. In order to evaluate the effect of water molecule on docking-based virtual screening simulations, 63 inhibitors and 1000 decoys molecules were docked against two NS5B polymerase crystal structures 3HHK and 3SKA, which contain bound inhibitors in the palm I region (see S12 Table in supporting information). The result of NW-docking (docking without water) have a similar effect to the W-docking (docking with water). As Barreca et al [8] described, water molecules can be replaced by an acceptor group. So we removed water molecules from the complexes in docking procedure to increase the diversity of selected compounds (see supporting information for detailed discussion) [14,25,34,66,67]. Three docking protocols (HTVS, SP and XP) and default docking parameters were used to reproduce their crystallized structures in the binding sites of the NS5B polymerase. Table 4 lists the RMSD values between the crystallized and redocked conformations of the six ligands. For the HTVS docking protocol, the RMSD values of 3SKA, 4DRU and 2GIR were less than 2 Å, while the RMSD values of 3HHK, 2BRK and 3PHE were 2.85, 6.30 and 6.21 Å, respectively. It can be observed clearly from Table 4 that both the Glide SP and XP docking protocols generated low RMSD values (< 1.5 Å) except 3HHK, whose SP and XP RMSD values were higher than 3 Å. These indicate that Glide SP and XP are dependable docking protocols for reproducing the crystal pose of the ligands in the active sites of the NS5B polymerase. Thus, the Glide SP and XP protocols and default parameters were used for further virtual screening processes.

**Table 4:** The RMSD (Å) for glide HTVS, SP and XP.

PDB entry	HTVS (RMSD Å)	SP (RMSD Å)	XP (RMSD Å)
3HHK	2.85	3.58	3.45
3SKA	1.00	1.20	1.20
2BRK	6.30	0.44	1.15
4DRU	0.42	0.56	0.37
2GIR	0.56	0.33	0.58
3PHE	6.21	0.82	0.48

### Virtual screening

After the validation of the RF model, the e-pharmacophore models and the docking protocols, we constructed a virtual screening process combining the RF-based virtual screening (RB-VS), the e-pharmacophore-based virtual screening (PB-VS) and the docking-based virtual screening (DB-VS) methods. In order to evaluate the performance of the multistage VS approach, we created a validation set that comprises 73 known HCV NS5B polymerase inhibitors and 2190 decoys from PubChem database to assess different VS methods (see S13 Table in supporting information). The result indicated that RB/PB/DB VS method was the most efficient combination (detailed discussion in supporting information). The RB-VS, PB-VS, and DB-VS were used in a hierarchical fashion that the fastest filter RB-VS was first applied, and the second fast filter PB-VS was subsequently applied, and the slowest filter DB-VS was finally applied. We also did a test of the data fusion model and evaluated the performance of data fusion method by screening NCI database (see S14 Table in supporting information). The number of results and time of the fusion method were therefore 1070 compounds and 7960 hours, respectively. And the number of result and time of the multistage method were therefore 539 compounds and 8 hours, respectively. As shown in S14 Table, the fusion method is a big improvement over single methods, but the result of multistage method is comparable in a tiny fraction of the time. A large chemical library, including 441,574 compounds from the InterBioscreen database, was used to retrieve new potent NS5B polymerase inhibitors. In the RB-VS stage, the RF Model III with 16 descriptors was used to screen the entire library. 51769 compounds passed through the RB-VS stage. These 51769 compounds were further screened by the six e-pharmacophore models in the PB-VS stage. Finally, the compounds filtered with the e-pharmacophore models were subjected to the DB-VS stage by using Glide SP and XP.

The number of hits from each of the 6 e-pharmacophore models and Glide docking (SP and XP) are presented in Table 5. To ensure chemotype diversity, we cluster hits of each screening stage by their Bemis−Murcko atomic frameworks [88,89]. For the palm I region, the e-pharmacophore model A5A6R14R16 for 3HHK yielded 2603 hits, belonging to 1289 clusters, when a fitness value of more than 1.6 was taken as the threshold. These 2603 compounds were then docked to the palm I region of 3HHK, and 911 hits belonging to 559 clusters were retrieved from the Glide SP docking with a docking score cutoff value of ≤ -6.0 kcal/mol. Subsequently, the Glide XP module was used to further down-size the 911 hits, and 783 hits belonging to 478 clusters were retrieved with a docking score cutoff value of ≤ -5.0 kcal/mol. Finally, the top 783 ligand molecules belonging to 478 clusters were visually inspected based on docking pose and their interactions with the important binding residues, and 23 hits with diverged structure scaffolds were selected. The e-pharmacophore model A2D3R9R10R11 from 3SKA retrieved 753 hits, belonging to 224 clusters, with a fitness value above 1.5. Glide SP docking retrieved 349 hits with a docking score cutoff value of ≤ -6.0 kcal/mol. Glide XP docking retrieved 294 hits with a docking score cutoff value of ≤ -5.0 kcal/mol. The top 294 ligand molecules, belonging to 150 clusters, were visually inspected based on docking pose and their interactions with the important binding residues, and 17 hits with diverged structure scaffolds were finally selected. Similar screening processes were carried out for the other e-pharmacophore models in the thumb I and thumb II regions. The e-pharmacophore model from 2BRK was restrictive and retrieved only 7 hits from the 51,769 compounds with a fitness value above 2.0. These indicated that different pharmacophore models derived from different protein complexes may have quite diverse performance from a screening compound database, and these pharmacophore models can retrieve diverse hits and improve the overall screening efficacy.

**Table 5:** Number of hits retrieved at the PB-VS and DB-VS stage of screening.

PDB code	number of phase identified hits	clusters	number of SP hits	clusters	number of XP hits	clusters	number of final hits
3HHK	2603	1289	911	559	783	478	23
3SKA	753	224	349	166	294	150	17
2BRK	16	12	10	9	8	7	7
4DRU	653	266	508	224	220	129	20
2GIR	5905	1817	1132	518	922	443	42
3PHE	789	316	221	122	184	113	7

The final hits found by the virtual screening process combining RB-VS, PB-VS and DB-VS were further inspected for synthesis availabilities (easiness) and expense limitations, and 5 compounds were finally selected (the synthesis and characterization were attached in S1 File) for the subsequent anti-HCV activity and cytotoxicity assay.

### Anti-replicon activity and cytotoxicity assays

To determine the inhibitory activities of the 5 hit compounds, we prepared an HCV cell culture system (HCVcc-hRluc-JFH1) with an HCV genotype 2a JFH-1 virus containing a humanized Rellina luciferase reporter gene (for experimental details, see materials and methods). The anti-HCV activities of the synthesized hit compounds were then evaluated using the HCV cell culture system, with PSI-7977 [90] and telaprevir [91] as positive control. To further judge the experimental results, we cited the reported anti-HCV data of compound 7r (an indole-based palm site inhibitor of HCV NS5B polymerase corresponding to 3SKA in S1 Fig) [61]. The results are summarized in Table 6. As shown, all 5 hit compounds displayed inhibitory activity against HCV (JFH-1, genotype 2a), with EC_50_ values ranging from 1.61 to 21.28 μM. Among them, the compound N2 exhibited more potent activities than the other hit compounds, with an EC_50_ value of 1.61 μM. The cytotoxicity of the hit compounds was determined by measuring the absorbance (OD450, reference OD630). The cytotoxicity testing showed that all compounds displayed no cellular cytotoxicity (CC_50_ > 100 μM) except the compound N2, which displayed weak cytotoxicity with a CC_50_ value of 51.3 μM. To further evaluate if the inhibition observed by compound N2 was due to cellular toxicity, we tested the inhibitory activity against HCV of the compound N2 at a concentration of 12.5 μM. The HCV virus titers were suppressed by almost 99% (see S3A Fig), but it displayed no cellular cytotoxicity (see S3B Fig), and thus compound N2 has a specific anti-HCV activity. The CC_50_ of compound 6a (in the paper of Eda Canales) [92] and compound 12e (in the paper of Kyeong Lee) [93] were therefore 44 μM and 61.8 μM, respectively. The hit compound N2 has the best antiviral activity against HCV, with a selective index (SI) of 32.1. These compounds may serve as a valuable candidate for the development of a new class of HCV NS5B polymerase inhibitors in the future.

**Table 6:** Antiviral activity and cytotoxicity of the 5 hits.

	SPR on NS5B	NS5B inhibition assay	Anti-HCV activity virus assay JFH1				
Compounds	K_D_ (μM)^*a*^	IC_50_ (μM)^*b*^	EC_50_ (μM)^*c*^	CC_50_ (μM)^*d*^	SI^*e*^	LLE (K_D_)^*f*^	LLE (IC_50_)^*g*^
N1	62.89	3.63	>12.5	>100	≥8.0	0.72	1.96
N2	4.67	3.00	1.61	51.3	31.1	0.10	0.29
N3	86.12	23.84	21.28	>100	4.7	2.65	3.21
N4	123.1	2.01	>12.5	>100	≥8.0	-0.34	1.45
N5	ND^*h*^	2.20	10.91	>100	9.2	—	1.47
PSI-7977^*i*^			0.49	>20	>40.82		
telaprevir^*j*^			0.02	>10	>500		
7r^*k*^			^*l*^0.3	—	—		

^*a*^ K_D_: Dissociation equilibrium constant. At this concentration 50% of all binding site are occupied.

^*b*^ IC_50_ (μM): Concentration of compound that inhibits 50% enzyme activity in vitro. 2’-O-Me-CTP was used as the positive control.

^*c*^ EC_50_ (μM) and

^*d*^ CC_50_ (μM): Each value indicates the mean of three experiments in each assay, and the values were plotted by the GraphPad Prism 5 software. The 50% inhibitory concentration (EC_50_) defined as the inhibitor concentration that reduced luminescence by 50%, the 50% cytotoxic concentration (CC_50_) defined as the compound concentration that inhibited cell growth by 50% and the

^*e*^ SI (selective index) is the ratio of CC_50_ to EC_50_.

^*f*^ LLE(K_D_) = pK_D_ − predicted log P.

^*g*^ LLE(IC_50_) = pIC_50_ − predicted log P.

^*h*^ ND: not determined.

^*i*^ PSI-7977 (sofosbuvir: nucleotide inhibitor of NS5B) and

^*j*^ telaprevir (inhibitor of NS3 protease) were used as the positive control.

^*k*^ 7r: an indole-based palm site inhibitor of HCV NS5B polymerase corresponding to 3SKA in S1 Fig.

^*l*^ 0.3: the data is cited from reference [61].

### SPR interaction analysis

In order to explore the binding affinity of hits for HCV NS5B polymerase, SPR was used to evaluate the interaction between the hits and NS5B polymerase. The dissociation constants (K_D_) for the binding to NS5B were determined for all compounds except N5. N5 might interact with the NS5B, but solubility issues possibly prevented a proper determination of the binding affinity. The Table 6 indicated the binding affinities of 4 compounds to NS5B polymerase are in the micromolar range (K_D_ = 4.67–123.1 μM). The ligand-lipophilic efficiency (LLE) calculated from K_D_ and IC_50_ values has become a popular measure to estimate druglikeness in drug discovery [94]. QikProp [95] was used to calculate the relevant parameter logP (see S15 Table in supporting information). N3 exhibited the highest LLE value (2.65), which could be a promising HTS hit. In particular, compound N2 displayed the best affinity (K_D_ = 4.67 μM) and the lowest LLE value of 0.10. Hence, N2 displayed a much worse potential druglikeness and higher logP value than others. Table 6 showed that there was a discrepancy between the measured EC_50_ and K_D_ values, which might cause by the fact that only partially occupied NS5B polymerases could achieve maximal anti-HCV activity [96]. These compounds could be designated as binders (or hits) of NS5B polymerase.

### NS5B polymerase inhibition assay

The inhibition of NS5B RdRp activity was evaluated by NS5B-catalyzed RNA synthesis assay. In this study, 2’-O-Me-CTP [78] was used as the positive control. IC_50_ values were obtained from the dose-response curves (see S4 Fig in supporting information). Five compounds tested were found to inhibit NS5B RdRp activity with IC_50_ values ranging from 2.01 to 23.84 μM, and with LLE values ranging from 0.29 to 3.21. Among the tested compounds, compound N4 exhibited the most potent activity and showed IC_50_ of 2.01 μM, and LLE of 1.45. However, its negative LLE calculated from K_D_ value was clearly unfavourable. In particular, compound N3 displayed the highest LLE of 3.21, with corresponding IC_50_ value of 23.84 μM. Thus, the inhibition of HCV replication in cell-based assays of the 5 hit compounds could be ascribed to targeting to NS5B polymerase.

### Analysis of the hit compounds

**Fig 4:**
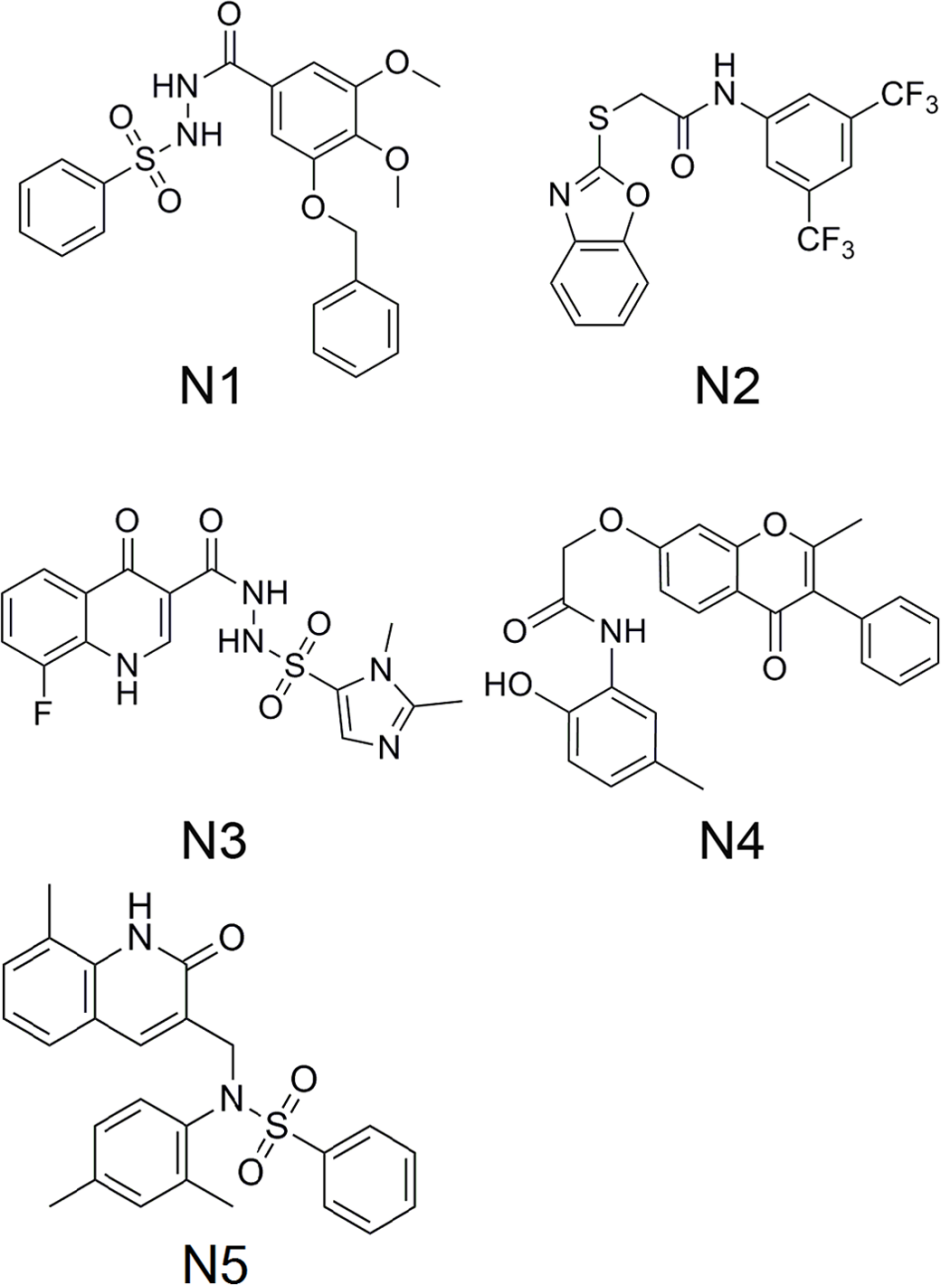
Structures of the 5 hit compounds.

**Fig 5:**
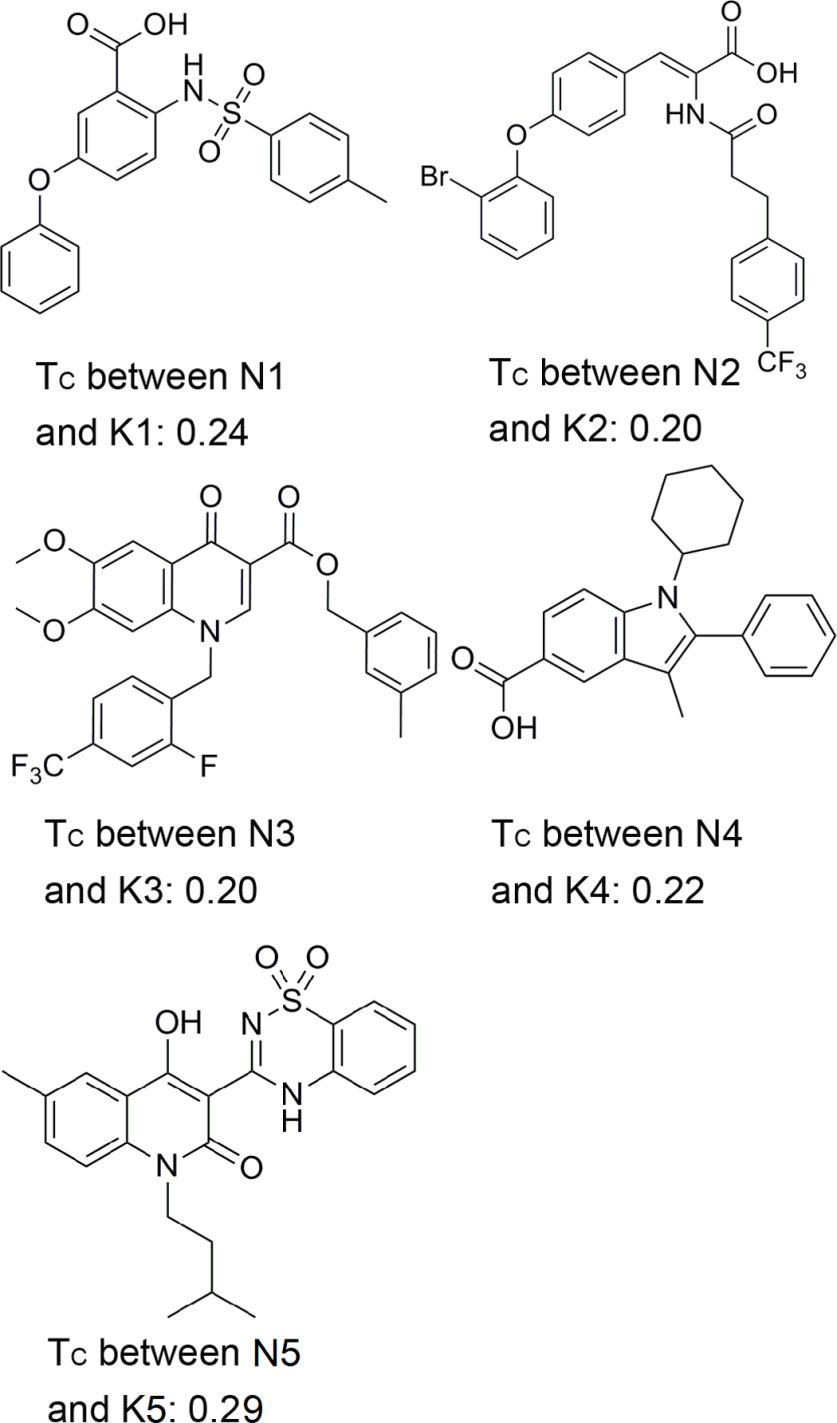
The structures of known NS5B inhibitors (K1 –K5). These inhibitors have the highest Tcs with N1 –N5, respectively.

The structures of the 5 hit compounds (N1 –N5) are shown in Fig 4. These hits belong to diverse chemotypes including benzenesulfonylhydrazine, benzoxazole, quinolinone, chromanone. These 5 compounds have new scaffolds and have never been reported as NS5B polymerase inhibitors. To further analyze the novelty of N1 –N5, a similarity calculation based on the extended connectivity fingerprints (ECFPs) was done to compare the Tanimoto coefficients (Tc) between the 5 compounds and all the known NS5B polymerase inhibitors[97]. The known NS5B inhibitors (K1 –K5) that have the largest Tcs with N1 –N5 were presented in Fig 5, respectively. The Tcs of N*i*—K*i* (*i* = 1, 2, 3, 4, 5) are all less than 0.3, which indicated that the chemical structures of N*i* are diversified from the K*i* compounds.

**Fig 6:**
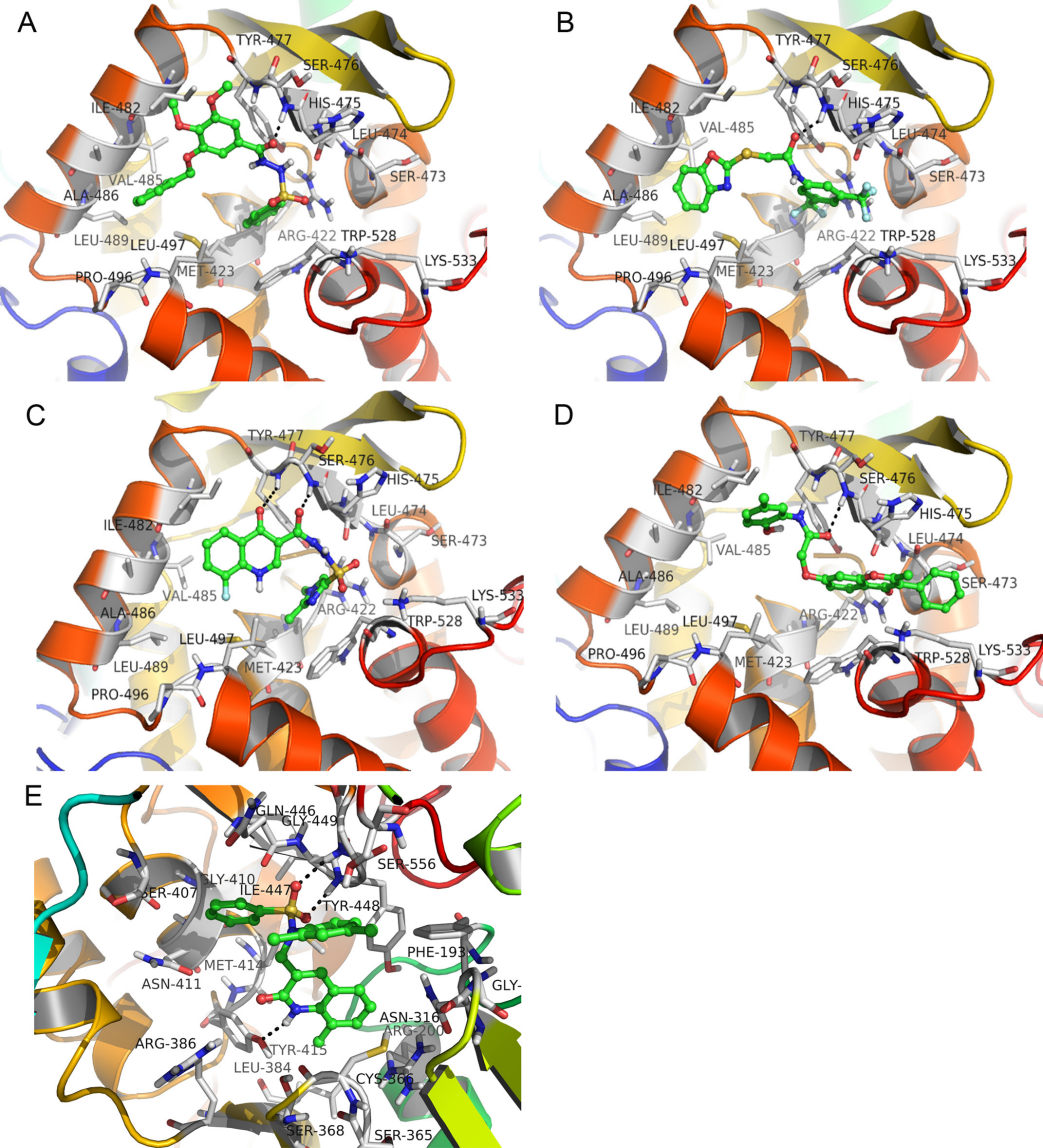
The binding poses of compound N1 –N5 in the respective binding pocket of NS5B polymerase. (A) N1; (B) N2; (C) N3; (D) N4; (E) N5. Potential hydrogen bonding interaction are shown as dashed lines.

The possible binding modes of N1 –N5 in their respective active sites of the NS5B polymerase are shown in Fig 6. We can see from Fig 6 that N1 –N4 bind to the thumb II region of the NS5B polymerase and participate in hydrogen bonding to the backbone amine of Ser476. From Fig 6A and 6B, we can see that the benzene rings of compound N1 and benzoxazole ring and trifluoromethyl group of compound N2 are directed toward the hydrophobic region. From Fig 6C, we can see that the carbonyl group from quinolinone of compound N3 forms a hydrogen bond with Tyr477. Again the fluorophenyl and dimethylimidazolyl groups are directed toward the hydrophobic region. From Fig 6D, we can see that the carbonyl group from chromanone of N4 forms two hydrogen bonds with Leu474 and Arg422. However, a lack of useful hydrophobic interactions makes the compound N4 showing weak binding affinity to NS5B polymerase with K_D_ value of 123.1 μM. Compound N5 binds to the palm region of NS5B polymerase. From Fig 6E, we can see that sulfuric acid of N5 forms two hydrogen bonds with Tyr448 and Gly449, respectively. The proposed binding mode of compound N5 suggests that the amide NH atom forms an important hydrogen bond to Tyr415. The good binding modes of N1 –N5 with NS5B polymerase provide a foundation for their anti-HCV bioactivity. And a table to compare the obtained results with different papers was attached in supporting information (see S16 Table) [25,93,98]. Our results along with the results in these papers (compared in S16 Table) give evidence that an in silico modeling method could be useful for future drug design.

## Conclusions

A virtual screening process including RB-VS, PB-VS and DB-VS was applied to identify the novel NS5B polymerase inhibitors. In this investigation, we applied the three virtual screening methods according to the criterion from simpleness to complexity. RB-VS, chiefly characterized by its rapid and simple computations, was used as the first filter. PB-VS and DB-VS were applied to screen a small subset of compound database after RB-VS because these two methods were time-consuming. The models used for the RB-VS and PB-VS were first established and validated. The RF Model III with 16 descriptors was used in the RB-VS stage. Six e-pharmacophore models from different crystal structures of the NS5B polymerase with ligands binding at the palm I, thumb I and thumb II regions were used in the PB-VS stage. The Glide SP and XP docking protocols with default parameters were used in the DB-VS stage. This multistage approach was then applied to screen a large chemical library including 441,574 compounds from the InterBioscreen database. From the final hits, we selected 5 compounds for further anti-HCV activity and cellular cytotoxicity assay, and all 5 compounds displayed certain inhibition against HCV with EC_50_ values ranging from 1.61 to 21.28 μM. The cytotoxicity testing showed that no compounds displayed cellular cytotoxicity (CC_50_ > 100 μM) except compound N2, which displayed weak cytotoxicity with a CC_50_ value of 51.3 μM. The hit compound N2 has a best antiviral activity against the HCV virus, with a selective index of 32.1. All 5 compounds showed inhibitory potency against NS5B polymerase with IC_50_ value of 2.01–23.84 μM. These compounds belong to novel and diverse chemotypes and could be further optimized and developed to be potent and highly active NS5B polymerase inhibitors.

## Supporting Information

S1 Fig Structures of the six co-crystalized ligands with their PDB IDs, resolutions and affinity values. (TIF)

S2 Fig Comparison of out-of-bag, training, and independent test set error rates for Random Forest on HCV NS5B data, as the number trees increases. The plot indicates that the OOB error rate tracks the test error rate fairly well once the number of trees is sufficiently large. The plot also illustrates the lack of overfitting once the training error reaches zero.
(TIF)

S3 Fig The Inhibitory activity against HCV of the compound N2 at a concentration of 12.5 μM. (TIF)

S4 Fig Dose-reponse curves of N1-N5. (TIF)

S1 File The synthesis and characterization of 5 compounds. (ZIP)

S1 Table Structures of the 772 compounds (in SMILE format) used for the training of the RF model together with their experimental bioactivities. (DOC)

S2 Table Structures of the 141 compounds (in SMILE format) used for the validation set of the RF model together with their experimental bioactivities. (DOC)

S3 Table Structures of the 116 compounds (in SMILE format) used for the validation of the evaluation of performances of the e-pharmacophore models together with their experimental bioactivities. (DOC)

S4 Table Important descriptors used in the RF model and their importance values. (DOC)

S5 Table Results of RF model validation by three datasets. (DOC)

S6 Table Results of the chosen randomly data set RF model and chosen by scaffolds RF model validation by independent test sets. (DOC)

S7 Table Validation of e-pharmacophore 3HHK models. (DOC)

S8 Table Validation of e-pharmacophore 3SKA models. (DOC)

S9 Table Validation of e-pharmacophore 4DRU models. (DOC)

S10 Table Validation of e-pharmacophore 2GIR models. (DOC)

S11 Table Validation of e-pharmacophore 3PHE models. (DOC)

S12 Table ROC AUCs and enrichment factors (EF) obtained for the set-noW, set-2W and set-3W proteins. (DOC)

S13 Table Evaluation results of the performance of various VS methods by screening a validation set. (DOC)

S14 Table The number of compounds from NCI database and time consumption after glide SP docking, e-pharmacophore, random Forest, multistage virtual screening and data fusion methods. (DOC)

S15 Table Values of pK_D_, pIC_50_ and logP calculated using the program QikProp for compounds N1-N5. (DOC)

S16 Table Compare the obtained results with different papers. (DOC)
