# Supplementary figures and images for: Discovery of Novel Hepatitis C Virus NS5B Polymerase Inhibitors by Combining Random Forest, Multiple e-Pharmacophore Modeling and Docking
Source: PLoS One. 2016 Feb 4;11(2):e0148181. doi: 10.1371/journal.pone.0148181 (PMC4742222; doi:10.1371/journal.pone.0148181)

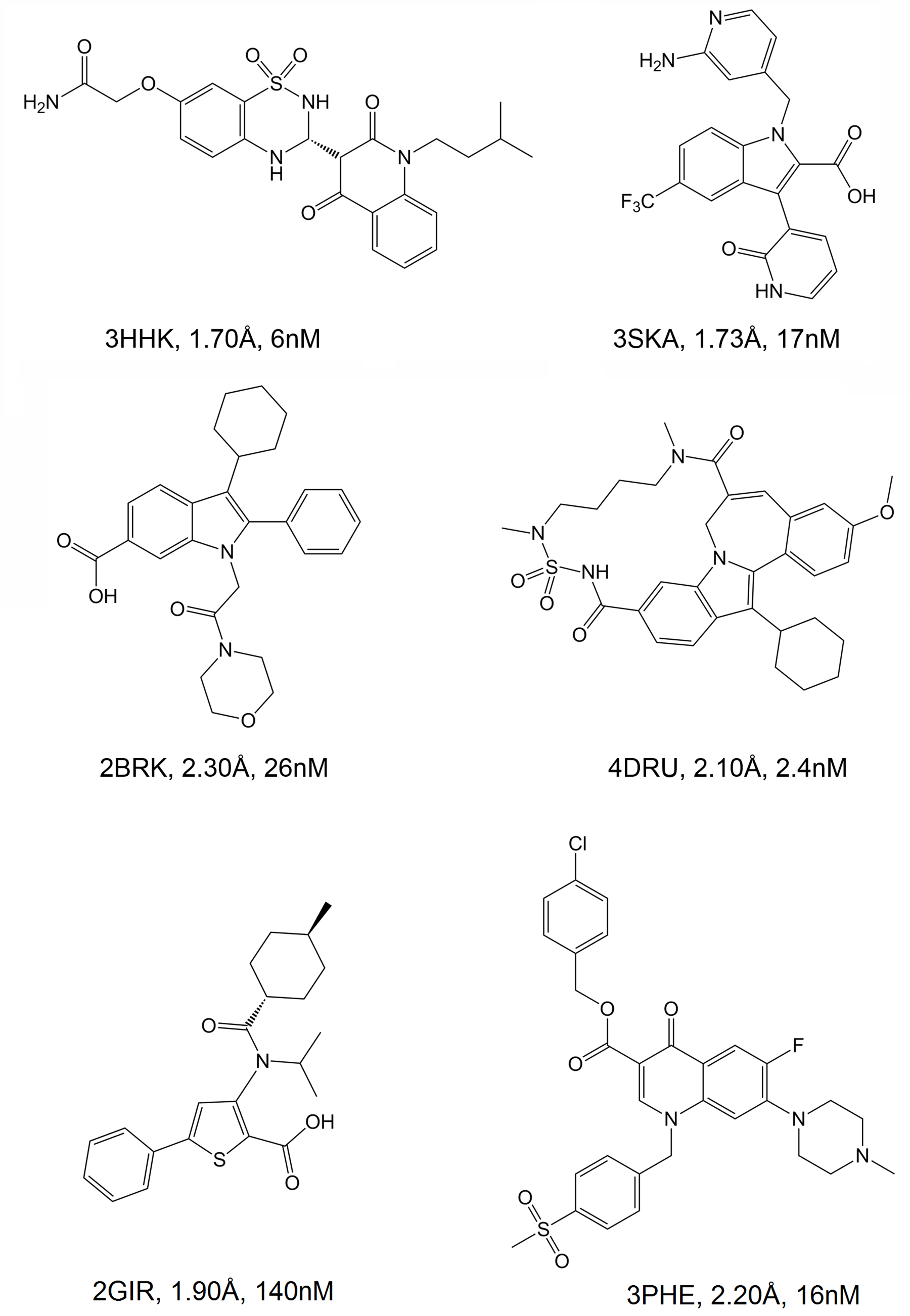

Supplement: S1 Fig — (TIF) [file pone.0148181.s001.tif]

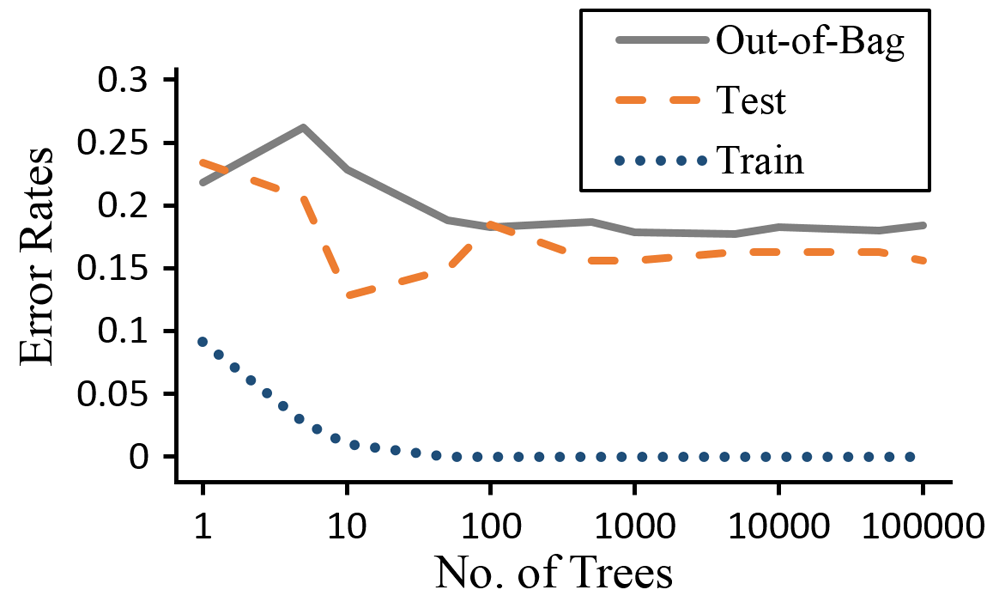

Supplement: S2 Fig — The plot indicates that the OOB error rate tracks the test error rate fairly well once the number of trees is sufficiently large. The plot also illustrates the lack of overfitting once the training error reaches zero. (TIF) [file pone.0148181.s002.tif]

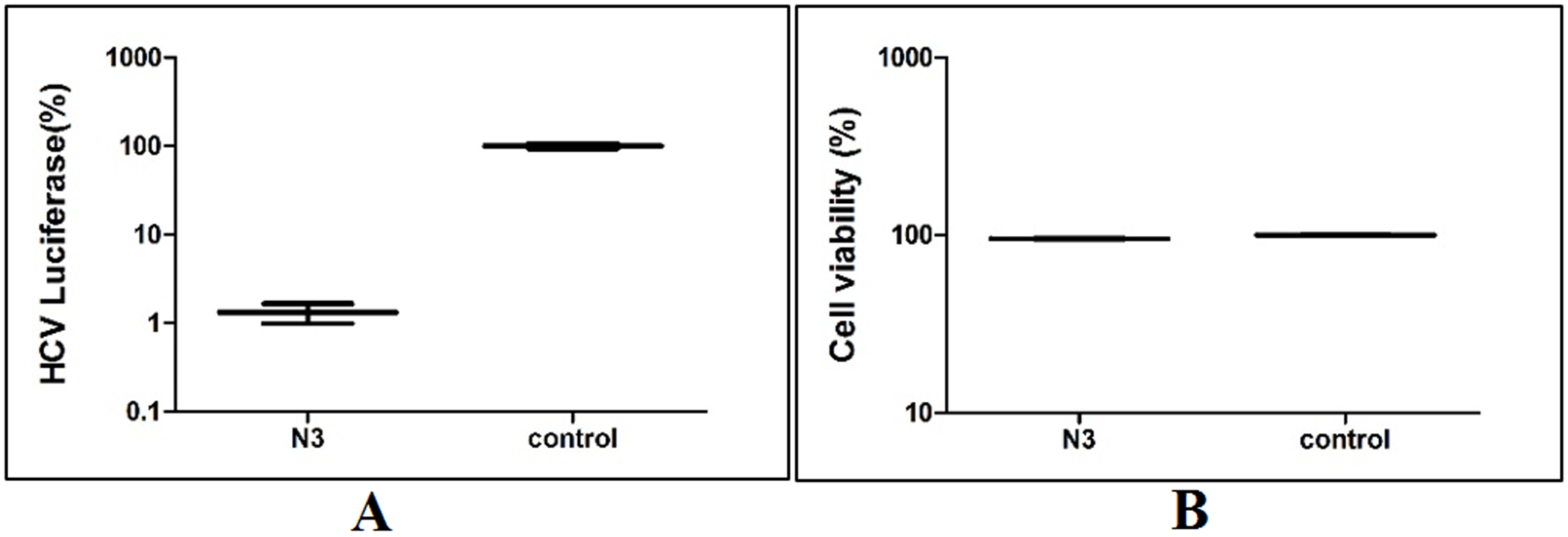

Supplement: S3 Fig — (TIF) [file pone.0148181.s003.tif]

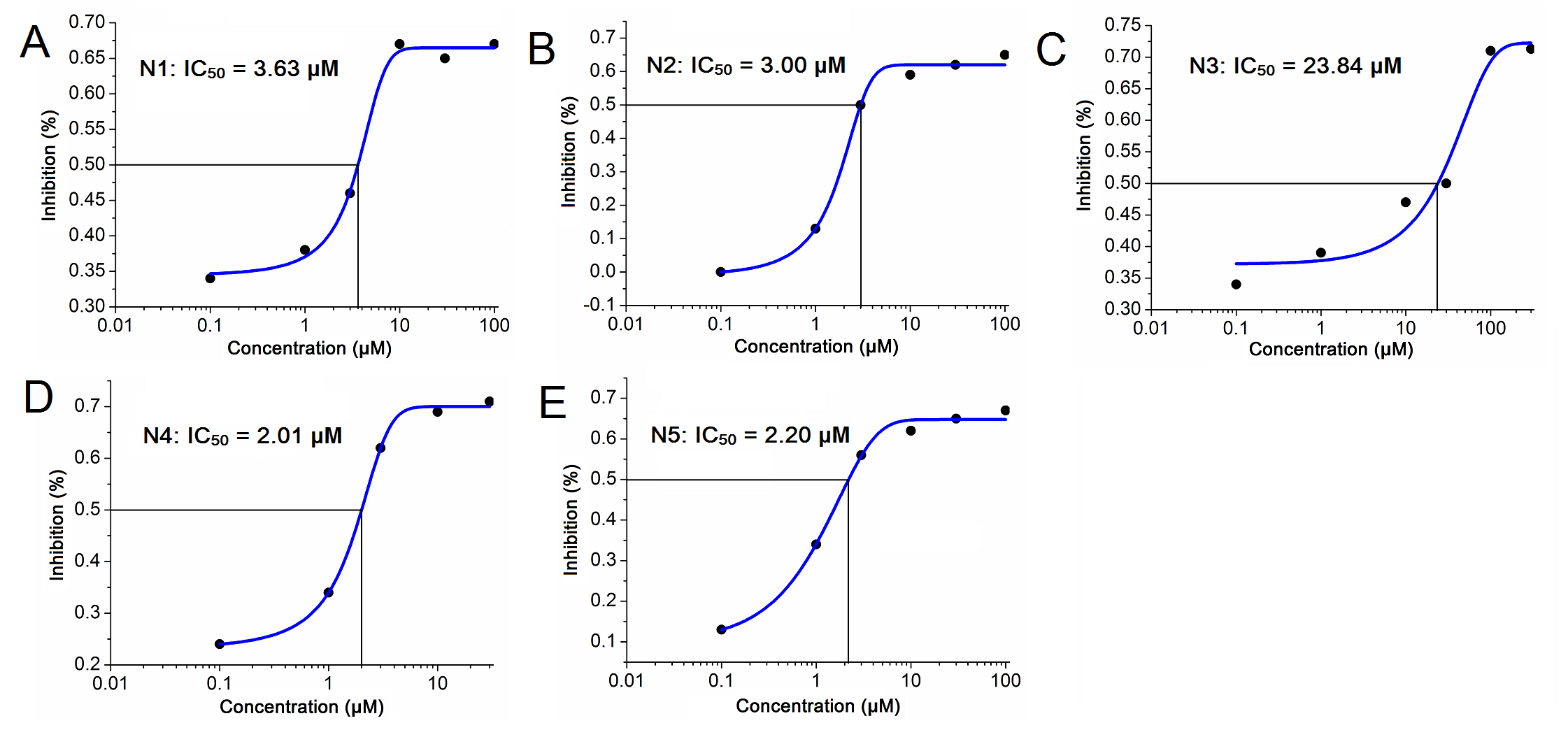

Supplement: S4 Fig — (TIF) [file pone.0148181.s004.tif]
