# Supplementary material for: Discovery of Novel Hepatitis C Virus NS5B Polymerase Inhibitors by Combining Random Forest, Multiple e-Pharmacophore Modeling and Docking
Source: PLoS One. 2016 Feb 4;11(2):e0148181. doi: 10.1371/journal.pone.0148181 (PMC4742222; doi:10.1371/journal.pone.0148181)
Supplement: S1 File — (ZIP) [file pone.0148181.s005.zip › N5 Final Report.doc]

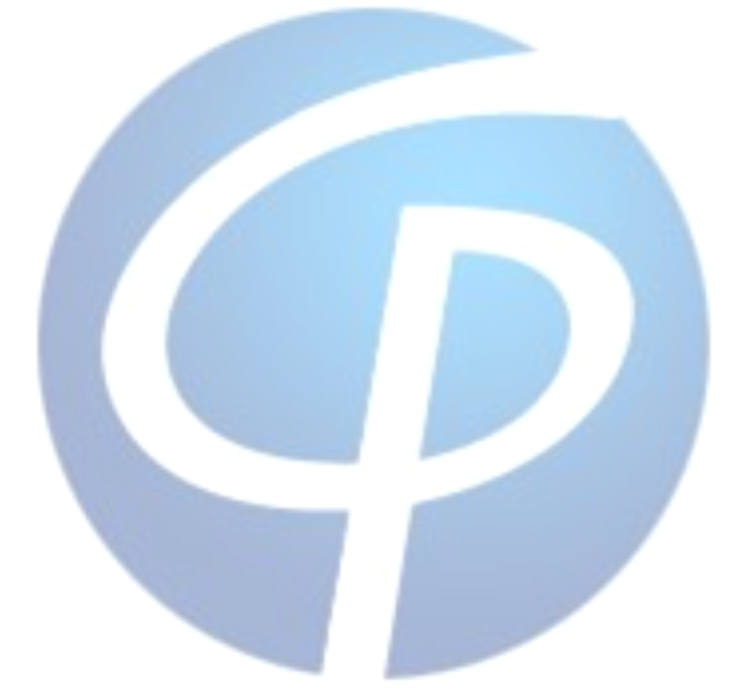


PracticaChem

**Project Report**

Code:N5

Project Leader: Li Wang

Project member: Jingjing Wang

1. **Delivery Information**

| Compound ID | Structure | Amount | Spectra | Delivery Date |
| --- | --- | --- | --- | --- |
| N5 |  | 20mg | NMR:  PN296-31 (20140624) |  |

# 2.Goal

To synthesize 20mg of **N5**

**3.Synthetic Route**


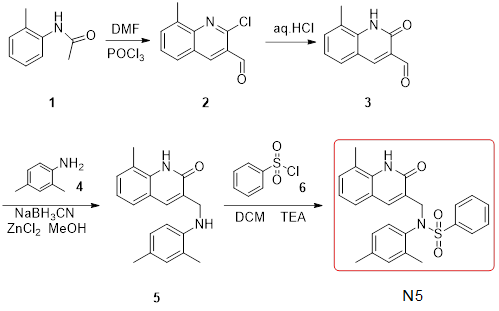
**4. Experimental and discussion**

**Compound 2**

**Experimental:PN296-26**

POCl_3_(36.44g ,234.6mmol) was added to DMF（6.5mL,83.79mmol） at 0℃ dropwised. then stirred for 30min. Compound **1**（5g ,33.51mmol）was added to the solution and refluxed at 75℃ for 16hrs. The reaction solution was poured into 250mL ice-water, stirred for 30min, filtered to get yellow solid, column with PE、PE/EA(50/1、20/1) get product 2.87g(yield, 41.65%).

**Compound 3**

**Experimental： PN296-28**

**2** (1.87g,9.09mmol) dissolved in 6N HCl (50 mL) then refluxed at 100℃ for 2h，monitored by TLC. The reaction was cooled to rt, adjust pH~9 with K2CO3, filtered and the solid was washed with water for 3 times, dried to give product 1.57g used in next step directly.

**Compound 5**

**Experimental: PN296-30**

Compound **3**（0.5g ,2.67mmol） suspend in MEOH（30mL）, **4**（0.66mL,5.34mmol），ZNCl_2_ （728mg ,5.34mmol）and NaBH_3_CN（335.7mg ,5.34mmol）was added, the reaction solution was stirred at rt for 2hrs, monitored by TLC. Concentrated to removed MeOH，EA and 1N NaOH、H_2_O added, aqueous layer was extracted with EA, combined EA, washed with water, Brine, dried with Na_2_SO_4_, concentrated and column with PE、PE/EA(10/1 、5/1 、4/1、 3/1、 2/1) to get product 570mg(**yield**：72.99%).

**Compound N5**

**
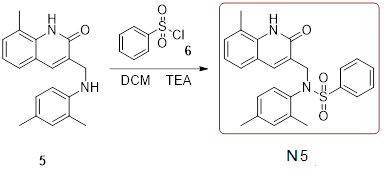
**

**Experimental: PN296-31**

Compound **5**(370mg , 1.27mol) dissolved in DCM（20mL）, TEA(0.53mL ,3.80mmol)，**6**（0.25mL ,1.90mmol） in DCM（10mL）was added to the solution, stirred at rt for 1.5hrs, monitored by TLC, DCM was added and washed with water, aq. NaHCO_3_, Brine, dried with Na_2_SO_4,_ column with PE、PE/EA(10/1、 5/1 、4/1 、3/1、 2/1) get product 460mg(yield：64.04%).

# 5. Spectrum

**TLC analysis:**


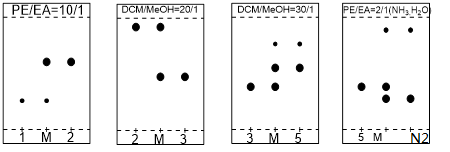


# H NMR

2-chloro-8-methylquinoline-3-carbaldehyde

N-(2,4-dimethylphenyl)-N-((8-methyl-2-oxo-1,2-dihydroquinolin-3-yl)methyl)benzenesulfonamide
