# Supplementary material for: Discovery of Novel Hepatitis C Virus NS5B Polymerase Inhibitors by Combining Random Forest, Multiple e-Pharmacophore Modeling and Docking
Source: PLoS One. 2016 Feb 4;11(2):e0148181. doi: 10.1371/journal.pone.0148181 (PMC4742222; doi:10.1371/journal.pone.0148181)
Supplement: S1 Table — (DOC) [file pone.0148181.s006.doc]

**S1 Table. Structures of the 772 compounds (in SMILE format) used for the training of the RF model together with their experimental bioactivities (in nM).**

| No. | Structure | Activity IC_50_ (nM) | Reference (DOI) |
| --- | --- | --- | --- |
| 1 | C[C@@H]1CC[C@H](CC1)c2ncccc2-c3cc(sc3C(=O)O)-c4ccccc4 | 0.022 | 10.1016/j.bmcl.2010.06.008 |
| 2 | C1C[C@@H](C)CC[C@H]1C(=O)N(C(C)C)c2cc(sc2C(=O)O)-c3ccccc3 | 0.028 | 10.1016/j.bmcl.2010.06.008 |
| 3 | C[C@@H]1CC[C@H](CC1)c2ccccc2-c3cc(sc3C(=O)O)-c4ccccc4 | 0.042 | 10.1016/j.bmcl.2010.06.008 |
| 4 | C[C@@H]1CC[C@H](CC1)n2nncc2-c3cc(sc3C(=O)O)-c4ccccc4 | 0.1 | 10.1016/j.bmcl.2010.06.008 |
| 5 | Cc1ccc(cc1)-n2nncc2-c3cc(sc3C(=O)O)-c4ccccc4 | 0.11 | 10.1016/j.bmcl.2010.06.008 |
| 6 | c1cccc(c12)n(NC3CCC3)c(=O)c(c2O)C(NS4(=O)=O)=Nc(c45)ccc(c5)NS(=O)(=O)N | 0.4 | 10.1016/j.bmcl.2006.04.015 |
| 7 | O=S(=O)(C)Nc(cc1)cc(c12)S(=O)(=O)C=C(N2)c(c3O)c(=O)n(c(c34)cccc4)Cc5ccc(F)cc5 | 2 | 10.1016/j.bmcl.2009.05.004 |
| 8 | Cc1ccc(cc1)-c2nocc2-c3cc(sc3C(=O)O)-c4ccccc4 | 2.5 | 10.1016/j.bmcl.2010.06.008 |
| 9 | c1ccnc(c12)n(CCC(C)C)c(=O)c(c2O)C(=NS3(=O)=O)Nc(c34)ccc(c4)NS(=O)(=O)N | 2.8 | 10.1016/j.bmcl.2006.04.015 |
| 10 | CCC[C@@](CC(=O)O)(OCC1)C(=C12)N=C3[C@@H]2C(C#N)=CC(=C3C)OC[C@H](C)n4cccn4 | 3 | 10.1016/j.bmcl.2011.04.052 |
| 11 | O=c1[nH]cccc1-c(c(c23)cc(C)cc2)c(C(=O)O)n3Cc4cc([N+](=O)[O-])ccc4F | 3 | 10.1021/jm201322r |
| 12 | O=c1[nH]cccc1-c2c(C(=O)NS(=O)(=O)C)n(c3ccc(c4c23)cco4)Cc5ccccc5Cl | 3 | 10.1016/j.bmcl.2013.01.024 |
| 13 | NS(=O)(=O)NCc(ccc1)c(c12)S(=O)(=O)N=C2C3=C(O)[C@H](C(C)(C)C)N(C3=O)Cc4ccc(F)cc4 | 3 | 10.1016/j.bmcl.2009.08.022 |
| 14 | c1coc2c1c(F)cc(c23)n(c(C(=O)O)c3-c4c(=O)[nH]ccc4)Cc(c(F)c5)cc(c56)c(=O)cc[nH]6 | 3 | 10.1016/j.bmcl.2013.11.007 |
| 15 | Cc1ccc(cc1)-c2cc(=O)[nH]nc2-c3cc(sc3C(=O)O)-c4ccccc4 | 3.3 | 10.1016/j.bmcl.2010.06.008 |
| 16 | O=c1[nH]cccc1-c(c(c23)cc(CC)cc2)c(C(=O)NS(=O)(=O)C4CC4)n3Cc5cc([N+](=O)[O-])ccc5F | 4 | 10.1021/jm201322r |
| 17 | [O-][N+](=O)c1ccc(F)c(c1)Cn(c(c23)cccc3)c(C(=O)NS(=O)(=O)C4CC4)c2-c5ccc[nH]c5=O | 4 | 10.1021/jm201322r |
| 18 | [O-][N+](=O)c1ccc(F)c(c1)Cn(c(c23)ccc(c3)C(F)(F)F)c(C(=O)NS(=O)(=O)C4CC4)c2-c5ccc[nH]c5=O | 4 | 10.1021/jm201322r |
| 19 | CS(=O)(=O)NCc(ccc1)c(c12)S(=O)(=O)N=C2C3=C(O)[C@H](C(C)(C)C)N(C3=O)Cc4ccc(F)c(c4)C | 4 | 10.1016/j.bmcl.2009.08.022 |
| 20 | c1coc2c1c(F)cc(c23)n(Cc(cc(n4)N)c(c45)cccc5)c(C(=O)O)c3-c6c(=O)[nH]ccc6 | 4 | 10.1016/j.bmcl.2013.11.007 |
| 21 | CCC[C@@](CC(=O)O)(OCC1)C(=C12)N=C3[C@@H]2C(C#N)=CC(=C3C)OCCn4cccn4 | 5 | 10.1016/j.bmcl.2011.04.052 |
| 22 | Fc1ccc(F)cc1Cn(c(c23)cc(F)c(c2)CC)c(C(=O)NS(=O)(=O)C4CC4)c3-c5ccc[nH]c5=O | 5 | 10.1021/jm201258k |
| 23 | [O-][N+](=O)c1ccc(F)c(c1)Cn(c(c23)ccc(F)c3)c(C(=O)NS(=O)(=O)C4CC4)c2-c5ccc[nH]c5=O | 5 | 10.1021/jm201322r |
| 24 | O=S(=O)(C)N(C)Cc(ccc1)c(c12)S(=O)(=O)N=C2C3=C(O)[C@H](C(C)(C)C)N(C3=O)Cc4ccc(F)c(c4)C | 5 | 10.1016/j.bmcl.2009.08.022 |
| 25 | CC(C)CC[C@H](n(c12)ccc2)C(=O)C(=C1O)C(=NS3(=O)=O)Nc(c34)ccc(c4)NS(=O)(=O)C | 5 | 10.1016/j.bmcl.2009.05.022 |
| 26 | CC(C)CCn1c(=O)c(c(O)n(c12)ccn2)C(=NS3(=O)=O)Nc(c34)ccc(c4)NS(=O)(=O)C | 5 | 10.1016/j.bmcl.2009.05.022 |
| 27 | CC(C)CCn1c(=O)c(c(O)n(c12)ncc2)C(=NS3(=O)=O)Nc(c34)ccc(c4)NS(=O)(=O)C | 5 | 10.1016/j.bmcl.2009.05.022 |
| 28 | c1ccccc1Cn2c(=O)c(c(O)n(c23)ncc3)C(=NS4(=O)=O)Nc(c45)ccc(c5)NS(=O)(=O)C | 5 | 10.1016/j.bmcl.2009.05.022 |
| 29 | O=S(=O)(C)Nc(cc1)cc(c12)S(=O)(=O)N=C(N2)c(c(O)n(c34)cccc3)c(=O)c4CCCCCC | 5 | 10.1016/j.bmcl.2009.05.021 |
| 30 | CC(C)S(=O)(=O)Nc(cc1)cc(c12)S(=O)(=O)N=C(N2)c(c(=O)c3CCC(C)C)c(O)n(c34)cccc4 | 5 | 10.1016/j.bmcl.2009.05.021 |
| 31 | O=S(=O)(C)Nc(cc1)cc(c12)S(=O)(=O)N=C(N2)c(c(=O)c3CCC(C)C)c(O)n(c34)CCCC4 | 5 | 10.1016/j.bmcl.2009.05.021 |
| 32 | O=S(=O)(C)Nc(cc1)cc(c12)S(=O)(=O)N=C(N2)c(c(=O)c3CCC(C)C)c(O)n(c34)cc(C)cc4 | 5 | 10.1016/j.bmcl.2009.05.021 |
| 33 | N#CCOc(c1)ccc(c12)NC(=NS2(=O)=O)[C@H](C3=O)C(=O)N(CCC(C)C)c(c34)ccc(F)c4 | 5 | 10.1016/j.bmcl.2009.05.091 |
| 34 | c1c(O)ccc(c12)NC(=NS2(=O)=O)[C@H](C3=O)C(=O)N(c(c34)ccc(F)c4)CCC5CC5 | 5 | 10.1016/j.bmcl.2009.05.091 |
| 35 | N#CCOc(c1)ccc(c12)NC(=NS2(=O)=O)[C@H](C3=O)C(=O)N(c(c34)ccc(F)c4)CCC5CC5 | 5 | 10.1016/j.bmcl.2009.05.091 |
| 36 | CNC(=O)COc(c1)ccc(c12)NC(=NS2(=O)=O)[C@H](C3=O)C(=O)N(c(c34)ccc(F)c4)CCC5CC5 | 5 | 10.1016/j.bmcl.2009.05.091 |
| 37 | CC(C)CCN(C1=O)[C@@H](C(C)(C)C)C(O)=C1C(=CS2(=O)=O)Nc(c23)ccc(c3)NS(=O)(=O)C | 5 | 10.1016/j.bmcl.2009.08.023 |
| 38 | c1coc2c1c(F)cc(c23)n(Cc(cc4)cc(c45)[nH]cn5)c(C(=O)O)c3-c6c(=O)[nH]ccc6 | 5 | 10.1016/j.bmcl.2013.11.007 |
| 39 | c1ccccc1-c(sc2C(=O)O)cc2-c3conc3-c4ccc(F)cc4 | 5.7 | 10.1016/j.bmcl.2010.06.008 |
| 40 | O=C(O)c(c1)ccc(c12)c(C3CCCCC3)c(n2CC(=O)N(C)C)-c4ccc(cc4)OCc5ccccc5 | 6 | 10.1021/jm049122i |
| 41 | O=c1[nH]cccc1-c(c(c23)cc(CC)cc2)c(C(=O)NS(=O)(=O)CC)n3Cc4cc(F)ccc4F | 6 | 10.1021/jm201258k |
| 42 | O=c1[nH]cccc1-c(c(c23)cc(C)cc2)c(C(=O)NS(=O)(=O)C4CC4)n3Cc5cc(F)ccc5F | 6 | 10.1021/jm201322r |
| 43 | O=c1[nH]cccc1-c(c(c23)cc(C(C)(C)C)cc2)c(C(=O)NS(=O)(=O)C4CC4)n3Cc5cc([N+](=O)[O-])ccc5F | 6 | 10.1021/jm201322r |
| 44 | o1ccc(c1c23)ccc3n(Cc(c(C)c4)ccc4C)c(C(=O)NS(=O)(=O)C)c2-c5ccc[nH]c5=O | 6 | 10.1016/j.bmcl.2013.01.024 |
| 45 | c1cc(C(=O)N)cc(c12)S(=O)(=O)N=C(N2)c(c3O)c(=O)n(CCC(C)C)c(c34)cccc4 | 6 | 10.1016/j.bmcl.2009.05.091 |
| 46 | c1cc(OC)cc(c12)nc(Cl)c(c2)Cn(c(c34)cc(F)c(c3)C)c(C(=O)O)c4-c5c(=O)[nH]ccc5 | 6 | 10.1016/j.bmcl.2013.10.060 |
| 47 | c1coc2c1c(F)cc(c23)n(Cc(c(F)c4)cc(c45)nc[nH]5)c(C(=O)O)c3-c6c(=O)[nH]ccc6 | 6 | 10.1016/j.bmcl.2013.11.007 |
| 48 | O=S(=O)(C)Nc(c1)ccc(c12)NC(=NS2(=O)=O)C(=C3O)C(=O)[C@@](C)(CCC(C)C)c(c34)cccc4 | 7 | 10.1016/j.bmcl.2004.12.030 |
| 49 | O=C(O)c(c1)ccc(c12)c(C3CCCCC3)c(-c4ccc(Cl)cc4)n2CC(=O)N(CC5)CCN5S(=O)(=O)C | 7 | 10.1021/jm050056+ |
| 50 | c1ccnc(c12)n(CCC(C)C)c(=O)c(c2O)C(=NS3(=O)=O)Nc(c34)ccc(c4)NS(=O)(=O)c5ccccc5 | 7 | 10.1016/j.bmcl.2006.04.022 |
| 51 | NC(=O)c1ccc(F)c(c1)Cn(c(c23)ccc(c3)C(F)(F)F)c(C(=O)O)c2-c4ccc[nH]c4=O | 7 | 10.1021/jm201258k |
| 52 | Fc1ccc(F)cc1Cn(c(c23)cc(C(F)(F)F)c(C)c2)c(C(=O)NS(=O)(=O)C4CC4)c3-c5ccc[nH]c5=O | 7 | 10.1021/jm201258k |
| 53 | O=c1[nH]cccc1-c(c(c23)cc(C)cc2)c(C(=O)NS(=O)(=O)C4CC4)n3Cc5cc([N+](=O)[O-])ccc5Cl | 7 | 10.1021/jm201322r |
| 54 | N#CCOc(c1)ccc(c12)NC(=NS2(=O)=O)[C@H](C3=O)C(=O)N(CCC(C)C)c(c34)cccc4 | 7 | 10.1016/j.bmcl.2009.05.091 |
| 55 | O=S(=O)(C)Nc(cc1)cc(c12)S(=O)(=O)C=C(N2)c(c3O)c(=O)n(c(c34)ccc(F)c4)Cc5ccccc5 | 7 | 10.1016/j.bmcl.2009.05.004 |
| 56 | c1cc(C)ccc1COC(=O)c(c(=O)c(c23)cc(OC)c(c2)OC)cn3Cc4ccc(C(F)(F)F)cc4F | 8 | 10.1016/j.bmcl.2010.11.068 |
| 57 | O=c1[nH]cccc1-c(c(c23)cc(C)cc2)c(C(=O)O)n3Cc4cc(F)ccc4F | 8 | 10.1021/jm201258k |
| 58 | O=c1[nH]cccc1-c2c(C(=O)NS(=O)(=O)C(C)C)n(c3ccc(c4c23)cco4)Cc5cc(F)ccc5F | 8 | 10.1016/j.bmcl.2013.01.024 |
| 59 | c1ccccc1CN(C2=O)[C@@H](C(C)(C)C)C(O)=C2C(=CS3(=O)=O)Nc(c34)ccc(c4)NS(=O)(=O)C | 8 | 10.1016/j.bmcl.2009.08.023 |
| 60 | O=S(=O)(C)Nc(cc1)cc(c12)S(=O)(=O)C=C(N2)c(c3O)c(=O)n(c(c34)c(F)ccc4)Cc5ccc(F)cc5 | 8 | 10.1016/j.bmcl.2009.05.004 |
| 61 | O=C(O)c(c1)ccc(c12)c(C3CCCCC3)c(-c4ccc(cc4)OC)n2CC(=O)N5CCC(CC5)N(CC)CC | 9 | 10.1021/jm050056+ |
| 62 | c1ccccc1-c2cc(c(s2)C(=O)O)N(C(C)C)C(=O)[C@@H]3CC[C@@H](C)CC3 | 9 | 10.1021/jm900517t |
| 63 | O=c1[nH]cccc1-c2c(C(=O)O)n(c3ccc(c4c23)scn4)Cc5cc(F)ccc5F | 9 | 10.1016/j.bmcl.2013.01.024 |
| 64 | O=S(=O)(C)Nc(c1)ccc(c12)NC(=NS2(=O)=O)C(=C3O)C(=O)C(CCCC)(CCCC)c(c34)cccc4 | 10 | 10.1016/j.bmcl.2008.06.043 |
| 65 | c1ccnc(c12)n(CCC(C)C)c(=O)c(c2O)C(=NS3(=O)=O)Nc(c34)ccc(c4)NS(=O)(=O)NCCN | 10 | 10.1016/j.bmcl.2006.04.015 |
| 66 | c1ccnc(c12)n(CCC(C)C)c(=O)c(c2O)C(=NS3(=O)=O)Nc(c34)ccc(c4)NS(=O)(=O)NCc5ccc(cc5)OC | 10 | 10.1016/j.bmcl.2006.04.015 |
| 67 | C1CC[C@@H]([C@H]12)N(CCC(C)C)C(=O)C(=C2O)C(N3)=NS(=O)(=O)c(c34)cc(cc4)NS(=O)(=O)C | 10 | 10.1016/j.bmcl.2008.11.048 |
| 68 | O=c1[nH]cccc1-c2c(C(=O)NS(=O)(=O)CC)n(c(c23)ccc(c3)C)Cc4cc(F)ccc4F | 10 | 10.1021/jm201258k |
| 69 | O=S(=O)(C)N(C)Cc(ccc1)c(c12)S(=O)(=O)N=C2C3=C(O)[C@H](C(C)(C)C)N(C3=O)Cc4ccc(F)c(Cl)c4 | 10 | 10.1016/j.bmcl.2009.08.022 |
| 70 | c1cccc(c12)NC(=NS2(=O)=O)[C@H](C3=O)C(=O)N(c(c34)ccc(F)c4)CCC5CC5 | 10 | 10.1016/j.bmcl.2009.05.091 |
| 71 | O=S(=O)(C)Nc(cc1)cc(c12)S(=O)(=O)C=C(N2)c(c3O)c(=O)n(c(c34)ccc(Cl)c4)Cc5ccc(F)cc5 | 10 | 10.1016/j.bmcl.2009.05.004 |
| 72 | O=S(=O)(C)Nc(cc1)cc(c12)S(=O)(=O)C=C(N2)c(c3O)c(=O)n(c(c34)cc(F)cc4)Cc5ccc(F)cc5 | 10 | 10.1016/j.bmcl.2009.05.004 |
| 73 | O=S(=O)(C)Nc(cc1)cc(c12)S(=O)(=O)C=C(N2)c(c3O)c(=O)n(c(c34)ccc(F)c4)CCC(C)(C)OC | 10 | 10.1016/j.bmcl.2009.05.004 |
| 74 | c1ccsc1-c2nn(CCC(C)C)c(=O)c(c2O)C(=N3)NS(=O)(=O)c(c34)cc(cc4)NS(=O)(=O)C | 10 | 10.1016/j.bmcl.2008.02.072 |
| 75 | c1ncsc1-c2nn(CCC(C)C)c(=O)c(c2O)C(=N3)NS(=O)(=O)c(c34)cc(cc4)NS(=O)(=O)C | 10 | 10.1016/j.bmcl.2008.02.072 |
| 76 | O=S(=O)(C)Nc(cc1)cc(c12)S(=O)(=O)NC(=N2)c(c3O)c(=O)n(CCC(C)C)nc3-c4ccsc4 | 10 | 10.1016/j.bmcl.2008.02.072 |
| 77 | c1ccsc1-c2nn(CC3CCC3)c(=O)c(c2O)C(=N4)NS(=O)(=O)c(c45)cc(cc5)NS(=O)(=O)C | 10 | 10.1016/j.bmcl.2008.02.072 |
| 78 | c1ccsc1-c2nn(CC3CCCC3)c(=O)c(c2O)C(=N4)NS(=O)(=O)c(c45)cc(cc5)NS(=O)(=O)C | 10 | 10.1016/j.bmcl.2008.02.072 |
| 79 | c1ccsc1-c2nn(Cc(cc3)ccc3F)c(=O)c(c2O)C(=N4)NS(=O)(=O)c(c45)cc(cc5)NS(=O)(=O)C | 10 | 10.1016/j.bmcl.2008.02.072 |
| 80 | C1COCCN1C(=O)Cn2c(-c3ccc(C)cc3)c(c(c24)ccc(c4)C(=O)O)C5CCCCC5 | 11 | 10.1021/jm049122i |
| 81 | c1cc(Cl)ccc1-c(n2CC(=O)N(C)C(C)C)c(c(c23)ccc(c3)C(=O)O)C4CCCCC4 | 11 | 10.1021/jm050056+ |
| 82 | OC(=O)c(c1)ccc(c12)c(C3CCCCC3)c4n2C[C@H]5[C@H](c6c4cccc6)N(CC5)CCCN7CCCCC7 | 11 | 10.1021/jm0610245 |
| 83 | c1ccsc1-c2nn(CC[C@H](C)CC)c(=O)c(c2O)C(=N3)NS(=O)(=O)c(c34)cc(cc4)NS(=O)(=O)C | 11 | 10.1016/j.bmcl.2008.02.072 |
| 84 | C1CCCCC1c2c(-c(cc3)ccc3F)cnc(n24)c(cn4)C(=O)N[C@H](C(=O)O)Cc5c[nH]c(c56)ccc(c6)O | 11 | 10.1016/j.bmcl.2009.09.087 |
| 85 | O=C(O)c(c1)ccc(c12)c(C3CCCCC3)c(-c4ccc(Cl)cc4)n2CC(=O)N5CCN(C)CC5 | 12 | 10.1021/jm050056+ |
| 86 | C1CCCCC1n(c(c23)ccc(c2)C(=O)O)c(n3)-c4ccc(cc4F)OCc5cc(C(=O)NC)ccc5-c6ccc(Cl)cc6 | 12 | 10.1021/jm060269e |
| 87 | c1cccc(c12)NC(=NS2(=O)=O)c(n(Cc(ccn3)cc3N)c(c45)ccc(Cl)c5)c4-c6ccc[nH]c6=O | 12 | 10.1016/j.bmcl.2011.10.041 |
| 88 | CN(C)C(=O)COc(c1)ccc(c12)NC(=NS2(=O)=O)[C@H](C3=O)C(=O)N(c(c34)ccc(F)c4)CCC5CC5 | 12 | 10.1016/j.bmcl.2009.05.091 |
| 89 | O=C(N)[C@H](C)Oc(c1)ccc(c12)NC(=NS2(=O)=O)[C@H](C3=O)C(=O)N(c(c34)ccc(F)c4)CCC5CC5 | 12 | 10.1016/j.bmcl.2009.05.091 |
| 90 | c1ccsc1-c2nn(Cc(cc3Cl)ccc3F)c(=O)c(c2O)C(=N4)NS(=O)(=O)c(c45)cc(cc5)NS(=O)(=O)C | 12 | 10.1016/j.bmcl.2008.02.072 |
| 91 | O=S(=O)(C)Nc(c1)ccc(c12)NC(=NS2(=O)=O)C(=C3O)C(=O)[C@](C)(c(c34)cccc4)CC5CCCCC5 | 13 | 10.1016/j.bmcl.2004.12.030 |
| 92 | CN(C)[C@H](C1)CN[C@H]1C(=O)Cn2c(-c3ccc(Cl)cc3)c(c(c24)ccc(c4)C(=O)O)C5CCCCC5 | 13 | 10.1021/jm050056+ |
| 93 | Fc1cc(F)ccc1CN(C2=O)[C@@H](C(C)(C)C)C(O)=C2C(=CS3(=O)=O)Nc(c34)ccc(c4)NS(=O)(=O)C | 13 | 10.1016/j.bmcl.2009.08.023 |
| 94 | c1ccnc(c12)n(CCC(C)C)c(=O)c(c2O)C(NS3(=O)=O)=Nc(c34)ccc(c4)NS(=O)(=O)NC | 14 | 10.1016/j.bmcl.2006.04.015 |
| 95 | C1CCCCC1n(c(c23)ccc(c2)C(=O)O)c(n3)-c4ccc(cc4F)OCc5cc(NC(=O)C)ccc5-c6ccc(Cl)cc6 | 14 | 10.1021/jm060269e |
| 96 | Cc1cc(C)nc(n12)nc(n2)SC(C(O3)=O)=C(O)C[C@]3(C4CCCC4)CCc5c(OC)cc(c(Cl)c5)OC | 14 | 10.1128/AAC.01008-06 |
| 97 | CN1CCN(CC1)c(c2)c(F)cc(c23)c(=O)c(cn3Cc4ccc(C(F)(F)F)cc4F)-c5noc(n5)Cc6ccccc6 | 14 | 10.1016/j.bmcl.2011.11.013 |
| 98 | O=c1[nH]cccc1-c(c(c23)cc(C)cc2)c(C(=O)NS(=O)(=O)C4CC4)n3Cc5c(F)c(F)cc(c5F)S(=O)(=O)C | 14 | 10.1021/jm201322r |
| 99 | OC(=O)/C=C/c1ccc(cc1)NC(=O)C2(CCCC2)NC(=O)c(cc3)cc(c34)n5c(c6c(OCC5)nccc6)c4C7CCCCC7 | 14 | 10.1016/j.bmcl.2012.02.063 |
| 100 | c1cocc1-c(n2)n(C3CCCCC3)c(c24)ccc(c4)C(=O)N[C@H](C(=O)O)Cc5c[nH]c(c56)ccc(c6)C7N=NN=N7 | 15 | 10.1016/j.bmcl.2003.12.032 |
| 101 | O=S(=O)(C)Nc(c1)ccc(c12)NC(=NS2(=O)=O)C(=C3O)C(=O)[C@@](C)(CCCC)c(c34)cccc4 | 15 | 10.1016/j.bmcl.2004.12.030 |
| 102 | c1scc(c12)c(=O)n(c(=O)[nH]2)-c3c(C(=O)NS(=O)(=O)C)n(c(c34)ccc(c4)C(F)(F)F)Cc5ccccc5F | 15 | 10.1016/j.bmcl.2011.10.041 |
| 103 | C1CCCCC1n(c(c23)ccc(c2)C(=O)O)c(n3)-c4ccc(cc4F)OCc5cc(N(C)C(=O)C)ccc5-c6ccc(Cl)cc6 | 16 | 10.1021/jm060269e |
| 104 | CN1CCN(CC1)c(c2)c(F)cc(c23)c(=O)c(cn3Cc4ccc(cc4)S(=O)(=O)C)C(=O)OCc5ccc(Cl)cc5 | 16 | 10.1016/j.bmcl.2010.11.068 |
| 105 | Cc1ccc(c(C)c1)Cn(c(c23)ccc(c3)C(F)(F)F)c(C(=O)O)c2-c4ccc[nH]c4=O | 16 | 10.1021/jm201258k |
| 106 | O=S(=O)(C)Nc(cc1)cc(c12)S(=O)(=O)C=C(N2)c(c3O)c(=O)n(c(c34)ccc(F)c4)Cc5cc(Cl)c(F)cc5 | 16 | 10.1016/j.bmcl.2009.05.004 |
| 107 | O=S(=O)(C)Nc(c1)ccc(c12)NC(=NS2(=O)=O)C(=C3O)C(=O)[C@@](CC)(CCC(C)C)c(c34)cccc4 | 17 | 10.1016/j.bmcl.2004.12.030 |
| 108 | OC(=O)c(c1)ccc(c12)c(C3CCCCC3)c4n2C[C@H]5[C@@H](N(CC5)CC(=O)N(C)C)c6c4cccc6 | 17 | 10.1021/jm0610245 |
| 109 | c1ccccc1-c(sc2C(=O)O)cc2-c3cnnn3-c4ccccc4 | 17 | 10.1016/j.bmcl.2010.06.008 |
| 110 | O=C(O)c(s1)cc(c12)cc(cc2)NC(=O)C3(CCN(C)CC3)NC(=O)c(cc4)cc(c45)n(C)c(-c6ccccn6)c5C7CCCCC7 | 17 | 10.1016/j.bmcl.2011.04.082 |
| 111 | FC(F)(F)c(c1)ccc(c12)n(Cc(ccn3)cc3N)c(C(=O)O)c2-c4ccc[nH]c4=O | 17 | 10.1021/jm201258k |
| 112 | O=C(O)c(c1)ccc(c12)c(C3CCCCC3)c(C4=CCCCC4)n2CC(=O)N5CCC(CC5)N(C)C | 18 | 10.1021/jm050056+ |
| 113 | c1cccc(c12)NC(=NS2(=O)=O)C3=C(O)[C@H](C(C)(C)C)N(C3=O)CCC(C)C | 18 | 10.1016/j.bmcl.2006.01.034 |
| 114 | c1ccnc(c12)n(CCC(C)C)c(=O)c(c2O)C(=NS3(=O)=O)Nc(c34)ccc(c4)NS(=O)(=O)Nc5ccc(cc5)C(=O)N | 18 | 10.1016/j.bmcl.2006.04.015 |
| 115 | C1CCCCC1n(c(c23)ccc(c2)C(=O)O)c(n3)-c4ccc(cc4)OCc5cc(C(=O)C)ccc5-c6ccc(Cl)cc6 | 18 | 10.1021/jm060269e |
| 116 | C1CCCCC1n(c(c23)ccc(c2)C(=O)O)c(n3)-c4ccc(cc4)OCc5cc(C(=O)NC)ccc5-c6ccc(Cl)cc6 | 18 | 10.1021/jm060269e |
| 117 | s1cccc1-c2nn(Cc3ccccc3)c(=O)c(c2O)C(N4)=CS(=O)(=O)c(c45)cc(cc5)NS(=O)(=O)C | 18 | 10.1016/j.bmcl.2008.07.014 |
| 118 | c1ccsc1-c2nn(CCC3CCCC3)c(=O)c(c2O)C(=N4)NS(=O)(=O)c(c45)cc(cc5)NS(=O)(=O)C | 18 | 10.1016/j.bmcl.2008.02.072 |
| 119 | C1CCCCC1n(c(c23)ccc(c2)C(=O)O)c(n3)-c4ccc(cc4F)OCc5cc(N(C(=O)C)CC)ccc5-c6ccc(Cl)cc6 | 19 | 10.1021/jm060269e |
| 120 | OC(=O)c(c1)ccc(c12)c(C3CCCCC3)c4n2CCN(C)c5c4cccc5 | 19 | 10.1021/jm0610245 |
| 121 | c1cocc1-c(n2)n(C3CCCCC3)c(c24)ccc(c4)C(=O)N[C@H](C(=O)O)Cc5c[nH]c(c56)ccc(c6)C(=O)O | 20 | 10.1016/j.bmcl.2003.12.032 |
| 122 | OC(=O)c(c1)ccc(c12)c(C3CCCCC3)c4n2C[C@H]5[C@@H](N(CC5)CCN(C)C)c6c4cccc6 | 20 | 10.1021/jm0610245 |
| 123 | c1c(F)cccc1CN([C@H]([C@@H]23)CCC3)C(=O)C(=C2O)C(N4)=NS(=O)(=O)c(c45)cc(cc5)NS(=O)(=O)C | 20 | 10.1016/j.bmcl.2008.11.048 |
| 124 | C1COCCN1c(c(F)c2)cc(c23)n(Cc4ccc(Cl)cc4)cc(c3=O)C(=O)OCc5ccc(Cl)cc5 | 20 | 10.1016/j.bmcl.2010.11.068 |
| 125 | OC(=O)/C=C/c1ccc(cc1)OC(=O)C2(CCCC2)NC(=O)c(cc3)cc(c34)n5c(c6c(NC(=O)C5)cccc6)c4C7CCCCC7 | 20 | 10.1016/j.bmcl.2011.03.067 |
| 126 | CCC[C@@](CC(=O)O)(OCC1)C(=C12)N=C3[C@@H]2C(C#N)=CC(=C3C)OCc4ccccn4 | 20 | 10.1016/j.bmcl.2011.04.052 |
| 127 | C1CCCCC1c2c(-c3cnccn3)n(C)c(c24)cc(cc4)C(=O)NC(C)(C)C(=O)Nc5ccc(cc5)/C=C/C(=O)O | 20 | 10.1016/j.bmcl.2011.04.059 |
| 128 | c1c(C)ccc(c12)nc(Cl)c(c2)Cn(c(c34)cc(F)c(c3)C)c(C(=O)O)c4-c5c(=O)[nH]ccc5 | 20 | 10.1016/j.bmcl.2013.10.060 |
| 129 | OC(=O)c(c1)ccc(c12)c(C3CCCCC3)c4n2C[C@H]5[C@H](c6c4ccc(c6)OC)N(CC5)C(=O)OCCN(C)C | 21 | 10.1021/jm0610245 |
| 130 | OC(=O)c(c1)ccc(c12)c(C3CCCCC3)c4n2C[C@H]5[C@H](N(CC5)CCN(C)C)c6c4ccc(c6)OC | 21 | 10.1021/jm0610245 |
| 131 | O=C(O)\C=C\c(cc1)ccc1NC(=O)[C@]2(CCN(C)C2)NC(=O)c(cc3)cc(c34)n(C)c(-c5ccccn5)c4C6CCCCC6 | 21 | 10.1016/j.bmcl.2011.04.082 |
| 132 | c1ccc(O)c(c12)NC3=C(C(=O)CC(C3)(C)C)[C@@H](N2C(=O)c4[nH]ncn4)c(c5F)cccc5OCc6ccccc6 | 21 | 10.1016/j.bmcl.2009.03.035 |
| 133 | C1CCCCC1n(c(c23)ccc(c2)C(=O)O)c(n3)-c4ccc(cc4)OCc5cc(NC(=O)C)ccc5-c6ccc(Cl)cc6 | 22 | 10.1021/jm060269e |
| 134 | O=S(=O)(C)Nc(cc1)cc(c12)S(=O)(=O)C=C(N2)c(c3O)c(=O)n(c(c34)ccc(F)c4)Cc5cccnc5 | 22 | 10.1016/j.bmcl.2009.05.004 |
| 135 | C=CCc(c1O)cn(CCC(C)C)c(=O)c1C(=NS2(=O)=O)Nc(c23)ccc(c3)NS(=O)(=O)C | 24 | 10.1016/j.bmcl.2004.12.030 |
| 136 | C1CCCCC1n(c(c23)ccc(c2)C(=O)O)c(n3)-c4ccc(cc4)OCc5cc(C(=O)O)ccc5-c6ccc(Cl)cc6 | 24 | 10.1021/jm060269e |
| 137 | O=c1[nH]cccc1-c2c(n(Cc(ccn3)cc3N)c(c24)ccc(Cl)c4)C(=O)NS(=O)(=O)c5ccc(O)cc5 | 24 | 10.1016/j.bmcl.2011.10.041 |
| 138 | CN1CCN(CC1)c(c2)c(F)cc(c23)c(=O)c(cn3Cc4ccc(C(F)(F)F)cc4)-c5noc(n5)Cc6ccccc6 | 24 | 10.1016/j.bmcl.2011.11.013 |
| 139 | OC(=O)/C=C/c1ccc(cc1)NC(=O)C2(CCCC2)NC(=O)c(cc3)cc(c34)n5c(c6c(cccn6)NC(=O)C5)c4C7CCCCC7 | 24 | 10.1016/j.bmcl.2012.02.063 |
| 140 | C1CCCN1c(c2O)nn(CCC(C)C)c(=O)c2C(N3)=NS(=O)(=O)c(c34)cc(cc4)OS(=O)(=O)C | 25 | 10.1016/j.bmcl.2008.08.094 |
| 141 | CS(=O)(=O)Nc(c1)ccc(c12)NC(=NS2(=O)=O)C(=C(O)C[C@@H]3CC)C(=O)N3Cc(cc4)ccc4F | 25 | 10.1016/j.bmcl.2009.09.051 |
| 142 | C1COCCN1C(=O)Cn2c(-c3ccccc3)c(c(c24)ccc(c4)C(=O)O)C5CCCCC5 | 26 | 10.1021/jm049122i |
| 143 | OC(=O)c(c1)ccc(c12)c(C3CCCCC3)c4n2CCN(c5c4cccc5)CCc6ccccc6 | 26 | 10.1021/jm0610245 |
| 144 | O=c1[nH]cccc1-c2c(n(Cc(ccn3)cc3N)c(c24)ccc(Cl)c4)C(=O)NS(=O)(=O)c5cc(F)cc(F)c5 | 26 | 10.1016/j.bmcl.2011.10.041 |
| 145 | C1CCCCC1c(c(cc2)c3cc24)c5n3[C@@H](C=Cc6c5ccc(c6)OC)C(=O)N(C)CCCCCN(C)S(=O)(=O)NC4=O | 26 | 10.1002/anie.201200110 |
| 146 | C1CCCN1NS(=O)(=O)Nc(c2)ccc(c23)NC(=NS3(=O)=O)c(c4O)c(=O)n(CCC(C)C)c(c45)nccc5 | 27 | 10.1016/j.bmcl.2006.04.015 |
| 147 | CC(C)CCn1c(=O)c(c(O)c(n12)cc(F)c2)C(N3)=NS(=O)(=O)c(c34)cc(cc4)NS(=O)(=O)C | 27 | 10.1016/j.bmcl.2008.04.066 |
| 148 | O=C(O)c(s1)c(C)c(c12)cc(cc2)NC(=O)C3(CCN(C)CC3)NC(=O)c(cc4)cc(c45)n(C)c(-c6ccccn6)c5C7CCCCC7 | 27 | 10.1016/j.bmcl.2011.04.082 |
| 149 | c1ccsc1-c2nn(CCC(C)C)c(=O)c(c2O)C(=N3)NS(=O)(=O)c(c34)cc(cc4)NS(=O)(=O)C5CC5 | 27 | 10.1016/j.bmcl.2008.02.072 |
| 150 | c1ccccc1-c(n2C)c(c(c23)ccc(c3)C(=O)O)C4CCCCC4 | 28 | 10.1021/jm049122i |
| 151 | OC(=O)c(c1)ccc(c12)c(C3CCCCC3)c4n2C[C@H]5[C@H](C[C@H](C5)N(C)C)c6c4cccc6 | 30 | 10.1021/jm0610245 |
| 152 | OC(=O)/C=C/c1ccc(cc1)NC(=O)C2(CCCC2)NC(=O)c(cc3)cc(c34)n5c(c6c(CCC5)nccc6)c4C7CCCCC7 | 30 | 10.1016/j.bmcl.2012.02.063 |
| 153 | O=c1[nH]cccc1-c2c(C(=O)O)n(c3ccc(c4c23)cco4)Cc5cc(F)ccc5F | 31 | 10.1016/j.bmcl.2013.01.024 |
| 154 | c1cccc(c12)c(O)c(c(F)c2CCC(C)C)C(=NS3(=O)=O)Nc(c34)ccc(c4)NS(=O)(=O)C | 31 | 10.1016/j.bmcl.2009.05.063 |
| 155 | O=S(=O)(C)Nc(c1)ccc(c12)NC(=NS2(=O)=O)C(=C3O)C(=O)[C@](C)(CC=C(C)C)c(c34)cccc4 | 32 | 10.1016/j.bmcl.2004.12.030 |
| 156 | c1cccc(c12)c(O)c(c(=O)n2CCC(C)C)C(N3)=NS(=O)(=O)c(c34)cccc4 | 32 | 10.1021/jm050855s |
| 157 | c1c(N)ccc(c12)n(CCC3CC3)c(=O)c(c2O)C(=NS4(=O)=O)Nc(c45)cccc5 | 32 | 10.1021/jm050855s |
| 158 | Clc1cccc(c12)n(CCC(C)C)c(=O)c(c2O)C(=NS3(=O)=O)Nc(c34)cccc4 | 33 | 10.1021/jm050855s |
| 159 | O=c1[nH]cccc1-c2c(C(=O)NS(=O)(=O)C)n(c(c23)ccc(Cl)c3)Cc4ccccc4F | 33 | 10.1016/j.bmcl.2011.10.041 |
| 160 | O1CC(=O)NCCCCCN(C)S(=O)(=O)NC(=O)c(cc2)cc(n3C)c2c(c3c(c14)cccc4)C5CCCCC5 | 33 | 10.1016/j.bmcl.2012.03.097 |
| 161 | C1CCCCC1c2c(-c3ccccn3)n(C)c(c24)cc(cc4)C(=O)NC(C)(C)C(=O)Nc5ccc(cc5)NC(=O)C(=O)O | 34 | 10.1016/j.bmcl.2011.04.082 |
| 162 | Cc1cc(F)ccc1S(=O)(=O)NC(=O)c(c2-c3ccc[nH]c3=O)n(c(c24)ccc(Cl)c4)Cc5ccnc(N)c5 | 34 | 10.1016/j.bmcl.2011.10.041 |
| 163 | O=c1[nH]cccc1-c2c(n(Cc(ccn3)cc3N)c(c24)ccc(Cl)c4)C(=O)NS(=O)(=O)c5cccc(c5)S(=O)(=O)N | 34 | 10.1016/j.bmcl.2011.10.041 |
| 164 | O=c1[nH]cccc1-c2c(C(=O)O)n(c3ccc(c4c23)occ4)Cc5cc(F)ccc5F | 34 | 10.1016/j.bmcl.2013.01.024 |
| 165 | s1cccc1-c(c2O)nn(CCC3CC3)c(=O)c2C(N4)=NS(=O)(=O)c(c45)cc(cc5)OCC(=O)N | 35 | 10.1016/j.bmcl.2008.01.007 |
| 166 | O=C(O)c1nc(nc(O)c1O)-c2c(ccs2)NC(=O)NCc3c(Cl)cccc3 | 36 | 10.1021/jm051064t |
| 167 | c1ccccc1Cn2c(-c3ccccc3)c(c(c24)ccc(c4)C(=O)O)C5CCCCC5 | 37 | 10.1021/jm049122i |
| 168 | c1cccc(c12)c(O)c(c(OC)c2CCC(C)C)C(=NS3(=O)=O)Nc(c34)ccc(c4)NS(=O)(=O)C | 37 | 10.1016/j.bmcl.2009.05.063 |
| 169 | s1cncc1-c(c2O)nn(CCC(C)C)c(=O)c2C(N3)=NS(=O)(=O)c(c34)cc(cc4)OCC(=O)N | 39 | 10.1016/j.bmcl.2008.01.007 |
| 170 | C1CCCC[C@@H]([C@H]12)N(CCC(C)(C)C)C(=O)C(=C2O)C(N3)=NS(=O)(=O)c(c34)cc(cc4)NS(=O)(=O)C | 39 | 10.1016/j.bmcl.2008.11.048 |
| 171 | O=S(=O)(C)Nc(c1)ccc(c12)NC(=NS2(=O)=O)C(=C3O)C(=O)[C@@](C)(CC4CC4)c(c35)cccc5 | 40 | 10.1016/j.bmcl.2004.12.030 |
| 172 | c1ccc(Cl)cc1-c(n2CC(=O)N(C)C)c(c(c23)ccc(c3)C(=O)O)C4CCCCC4 | 40 | 10.1021/jm049122i |
| 173 | c1ccccc1C(=O)N/C(C(=O)O)=C\c2ccc(cc2)Oc3c(I)cccc3 | 40 | 10.1016/j.bmcl.2005.03.066 |
| 174 | C1CCC[C@@H]([C@@H]12)N(CCC(C)C)C(=O)C(=C2O)C(N3)=NS(=O)(=O)c(c34)cc(cc4)NS(=O)(=O)C | 40 | 10.1016/j.bmcl.2008.11.048 |
| 175 | CCC[C@](CC(=O)O)(OCC1)C(=C12)N=C3[C@@H]2C(C#N)=CC(=C3C)OCCOC | 40 | 10.1016/j.bmcl.2011.04.052 |
| 176 | O=C(O)\C=C\c(cc1)ccc1NC(=O)C(C)(C)NC(=O)c(cc2)cc(c23)nc(-c4cocc4)n3C5CCCCC5 | 40 | 10.1016/j.bmcl.2011.04.059 |
| 177 | c1ccc(O)c(c12)NC3=C(C(=O)CC(C3)(C)C)[C@@H](N2C(=O)C)c(c(Cl)c4)ccc4OCc5ccccc5 | 40 | 10.1016/j.bmcl.2009.03.035 |
| 178 | CC(C)C[C@@]1(C(=O)O)C[C@H](C(=O)NS(=O)(=O)C)[C@H](c2nccs2)N1C(=O)c3ccc(cc3)C(C)(C)C | 40 | 10.1016/j.bmcl.2007.01.034 |
| 179 | c1cccc(c12)n(C[C@@H]3C[C@H]3C)c(=O)c(c2O)C(=NS4(=O)=O)Nc(c45)cccc5 | 41 | 10.1021/jm050855s |
| 180 | C1CCCCC1c2c(-c3cncnc3)n(C)c(c24)cc(cc4)C(=O)NC(C)(C)C(=O)Nc5ccc(cc5)/C=C/C(=O)O | 41 | 10.1016/j.bmcl.2011.04.059 |
| 181 | c1cc(Cl)ccc1Cn2cc(c(=O)c(c23)cc(OC)c(c3)OC)-c4noc(n4)Cc5ccccc5 | 41 | 10.1016/j.bmcl.2011.11.013 |
| 182 | c1cc(F)ccc1CN([C@H]([C@H]23)CCCCC3)C(=O)C(=C2O)C(N4)=NS(=O)(=O)c(c45)cc(cc5)NS(=O)(=O)C | 42 | 10.1016/j.bmcl.2008.11.048 |
| 183 | n1cc(F)ccc1CN(C2=O)[C@@H](C(C)(C)C)C(O)=C2C(=CS3(=O)=O)Nc(c34)ccc(c4)NS(=O)(=O)C | 42 | 10.1016/j.bmcl.2009.08.023 |
| 184 | CS(=O)(=O)Nc(c1)ccc(c12)NC(=NS2(=O)=O)C(=C(O)C[C@@H]3C(C)(C)C)C(=O)N3Cc(cc4)ccc4F | 42 | 10.1016/j.bmcl.2009.09.051 |
| 185 | n1nn[nH]c1-c(cn2)c(n23)ncc(c3C4CCCCC4)-c(cc5)ccc5OCc(cc6)ccc6C(=O)O | 42 | 10.1016/j.bmcl.2009.09.087 |
| 186 | O=S1(=O)CCN(CC1)C(=O)Cn2c(-c3ccccc3)c(c(c24)ccc(c4)C(=O)O)C5CCCCC5 | 43 | 10.1021/jm050056+ |
| 187 | c1c(Cl)ccc(c12)n(CCC(C)C)c(=O)c(c2O)C(=NS3(=O)=O)Nc(c34)cccc4 | 43 | 10.1021/jm050855s |
| 188 | C1CCC[C@@H]([C@@H]12)N(CCC(C)(C)C)C(=O)C(=C2O)C(N3)=NS(=O)(=O)c(c34)cc(cc4)NS(=O)(=O)C | 43 | 10.1016/j.bmcl.2008.11.048 |
| 189 | O=c1[nH]cccc1-c2c(n(Cc(ccn3)cc3N)c(c24)ccc(Cl)c4)C(=O)NS(=O)(=O)c5cccc(Cl)c5 | 43 | 10.1016/j.bmcl.2011.10.041 |
| 190 | O=C(O)c(c1)n(C)c(c12)ccc(c2)NC(=O)[C@]3(CCCNC3)NC(=O)c(cc4)cc(c45)n(C)c(-c6ccccn6)c5C7CCCCC7 | 44 | 10.1016/j.bmcl.2011.04.082 |
| 191 | O=C(O)C(=O)/C=C(O)/c1cc(ccc1)OCc2cc(Cl)cc(Cl)c2C#N | 45 | 10.1021/jm0342109 |
| 192 | C1CCCCN1NS(=O)(=O)Nc(c2)ccc(c23)NC(=NS3(=O)=O)c(c4O)c(=O)n(CCC(C)C)c(c45)nccc5 | 45 | 10.1016/j.bmcl.2006.04.015 |
| 193 | CC(C)(C)CCN([C@H]1C(C)(C)C)C(=O)C(=C1O)C2=NS(=O)(=O)c(c23)c(ccc3)CCS(=O)(=O)C | 45 | 10.1016/j.bmcl.2008.05.083 |
| 194 | c1ccccc1CN([C@@H]([C@@H]23)CCC3)C(=O)C(=C2O)C(N4)=NS(=O)(=O)c(c45)cc(cc5)NS(=O)(=O)C | 46 | 10.1016/j.bmcl.2008.11.048 |
| 195 | c1ccccc1-c([nH]2)c(c(c23)ccc(c3)C(=O)O)C4CCCCC4 | 48 | 10.1016/j.bmcl.2006.05.012 |
| 196 | c1cccc(c12)c(OC)c(cc2CCC(C)C)C(=NS3(=O)=O)Nc(c34)ccc(c4)NS(=O)(=O)C | 48 | 10.1016/j.bmcl.2009.05.063 |
| 197 | O=C(O)c(c1)ccc(c12)c(C3CCCCC3)c(C=C)n2CC(=O)N4CCC(CC4)N(C)C | 49 | 10.1021/jm050056+ |
| 198 | Clc1cc(Cl)c(Cl)cc1S(=O)(=O)Nc(ccc2)cc2\C(O)=C\C(=O)C(=O)O | 49 | 10.1021/jm0504454 |
| 199 | CS(=O)(=O)Nc(c1)ccc(c12)NC(=NS2(=O)=O)C(=C3O)C(=O)N(C4CCC4)C[C@@]3(C)CCCC | 49 | 10.1016/j.bmcl.2009.09.051 |
| 200 | c1cocc1-c(n2)n(C3CCCCC3)c(c24)ccc(c4)C(=O)N[C@H](C(=O)O)Cc5c[nH]c(c56)ccc(c6)O | 50 | 10.1016/j.bmcl.2003.12.032 |
| 201 | c1cccc(CC)c1C(=O)N/C(C(=O)O)=C\c2ccc(cc2)Oc3c(Br)cccc3 | 50 | 10.1016/j.bmcl.2005.03.106 |
| 202 | C1CC(C)(C)Cc(c12)sc(c2C(=O)OCC)NC(=O)NS(=O)(=O)c3ccnn3C | 50 | 10.1016/j.bmcl.2005.09.047 |
| 203 | C1CCCCC1n(c(c23)ccc(c2)C(=O)O)c(n3)-c4ccc(cc4)OCc5ccccc5-c6ccc(Cl)cc6 | 50 | 10.1021/jm060269e |
| 204 | c1ccc(O)c(c12)NC3=C(C(=O)CC(C3)(C)C)[C@@H](N2C(=O)C)c(c4Cl)cccc4OCc5ccccc5 | 50 | 10.1016/j.bmcl.2009.03.035 |
| 205 | c1ccnc(c12)n(CCC(C)C)c(=O)c(c2O)C(=NS3(=O)=O)Nc(c34)ccc(c4)OCCN | 51 | 10.1016/j.bmcl.2006.04.022 |
| 206 | c1ccc(O)c(c12)NC3=C(C(=O)CC(C3)(C)C)[C@@H](N2C(=O)c4ccccn4)c(c5F)cccc5OCc6ccccc6 | 51 | 10.1016/j.bmcl.2009.03.035 |
| 207 | Nc1cc(ccn1)Cn(c(c23)ccc(Cl)c3)c(C(=O)O)c2-c4ccc[nH]c4=O | 53 | 10.1021/jm201322r |
| 208 | COc(cc1)cc(c12)OCC(=O)N(C)CCCCN(C)S(=O)(=O)NC(=O)c3ccc4c(c3)n(C)c2c4C5CCCCC5 | 54 | 10.1016/j.bmcl.2012.03.097 |
| 209 | c1ccnc(c12)n(CCC(C)C)c(=O)c(c2O)C(=NS3(=O)=O)Nc(c34)ccc(c4)NS(=O)(=O)NCc5ccccc5OC | 57 | 10.1016/j.bmcl.2006.04.015 |
| 210 | c1cc(C#N)cc(c12)S(=O)(=O)N=C(N2)c(c3O)c(=O)n(CCC(C)C)c(c34)cccc4 | 57 | 10.1016/j.bmcl.2009.05.091 |
| 211 | c1ccccc1-c(n(CC(=O)N(C)C)c(c23)cc(s3)C(=O)O)c2C4CCCCC4 | 58 | 10.1016/j.bmcl.2006.05.012 |
| 212 | c1cccc(c12)NC(=NS2(=O)=O)C3=C(O)[C@H](C(C)(C)C)N(C3=O)Cc4ccc(F)cc4 | 59 | 10.1016/j.bmcl.2006.01.034 |
| 213 | c1cocc1-c(n2)n(C3CCCCC3)c(c24)ccc(c4)C(=O)N[C@H](C(=O)O)Cc5c[nH]c(c56)ccc(c6)NC(=O)C | 60 | 10.1016/j.bmcl.2003.12.032 |
| 214 | C1CC(C)(C)Cc(c12)sc(c2C(=O)OCC)NC(=O)NS(=O)(=O)c3ccc(F)cc3 | 60 | 10.1016/j.bmcl.2005.09.047 |
| 215 | CC(C)(C)CCn1c(=O)c(c(O)c(n12)ccc2)C(N3)=NS(=O)(=O)c(c34)cc(cc4)N(C)S(=O)(=O)C | 60 | 10.1016/j.bmcl.2008.04.066 |
| 216 | O=C(O)C(=N1)C=C(C12)CC(=CC=2)NC(=O)[C@H](C)NC(=O)c(cc3)cc(c34)nc(-c5cocc5)n4C6CCCCC6 | 60 | 10.1016/j.bmcl.2009.10.136 |
| 217 | Cc(c1)cccc1COC(=O)c(c(=O)c(c23)cc(OC)c(c2)OC)cn3Cc4ccc(C(F)(F)F)cc4F | 60 | 10.1016/j.bmcl.2010.11.068 |
| 218 | Fc1cc(F)cc(F)c1Cn2cnc(=O)c(c23)cc(cc3)Oc4c(C(F)(F)F)nccc4 | 60 | 10.1016/j.bmcl.2013.05.037 |
| 219 | c1cccc(c12)nc(Cl)c(c2Cl)Cn(c(c34)cc(F)c(c3)C)c(C(=O)O)c4-c5c(=O)[nH]ccc5 | 60 | 10.1016/j.bmcl.2013.10.060 |
| 220 | CS(=O)(=O)Nc(c1)ccc(c12)NC(=NS2(=O)=O)C(=C3O)C(=O)N(C4CCCC4)C[C@@]3(C)CCC(C)C | 61 | 10.1016/j.bmcl.2009.09.051 |
| 221 | s1cccc1-c(c2O)nn(CCC(C)C)c(=O)c2C(N3)=NS(=O)(=O)c(c34)cc(cc4)O[C@H](C)C(=O)N | 62 | 10.1016/j.bmcl.2008.01.007 |
| 222 | C1CC1CNC(=O)Cn2c(-c3ccccc3)c(c(c24)ccc(c4)C(=O)O)C5CCCCC5 | 63 | 10.1021/jm050056+ |
| 223 | C1CCCCC1n(c(c23)ccc(c2)C(=O)O)c(n3)-c4ccc(cc4)OCc5cc(N(C)C)ccc5-c6ccc(Cl)cc6 | 63 | 10.1021/jm060269e |
| 224 | CC(C)(C)C#Cc(sc1C(=O)O)cc1N(C(=O)[C@@H](CC2)CC[C@H]2C)N(C)C3CCN(C)CC3 | 63 | 10.1016/j.bmcl.2012.05.025 |
| 225 | OCCNc1ccc(c2c13)C(=O)N(C(=O)c2ccc3)c4cccc(Br)c4 | 64 | 10.1021/jm900517t |
| 226 | C1CCCCC1c2c(-c3ccccn3)n(C)c(c24)cc(cc4)C(=O)NC5(CCNC5)C(=O)Nc6ccc(cc6)-c7cncnc7 | 64 | 10.1016/j.bmcl.2011.04.082 |
| 227 | c1cccc(c12)n(CC[C@@H](C)CC)c(=O)c(c2O)C(=NS3(=O)=O)Nc(c34)cccc4 | 65 | 10.1021/jm050855s |
| 228 | C1CCCCC1c2c(-c3ccccn3)n(C)c(c24)cc(cc4)C(=O)NC5(CCOCC5)C(=O)Nc6ccc(cc6)/C=C/C(=O)O | 68 | 10.1016/j.bmcl.2011.04.082 |
| 229 | O=S(=O)(C)NCc(cc1)cc(c12)S(=O)(=O)C=C(N2)c(c3O)c(=O)n(c(c34)ccc(F)c4)Cc5ccc(F)cc5 | 68 | 10.1016/j.bmcl.2009.05.004 |
| 230 | N#CCOc(ccc1)c(c12)S(=O)(=O)N=C2C(=C3O)C(=O)N([C@H]3C(C)(C)C)Cc4cc(Cl)c(F)cc4 | 70 | 10.1016/j.bmcl.2008.05.083 |
| 231 | OC(=O)c(cc1)cc(c12)n3c(c4c(NC(=O)C3)cccc4)c2C5CCCCC5 | 70 | 10.1016/j.bmcl.2011.03.067 |
| 232 | c1ccc(O)c(c12)NC3=C(C(=O)CC(C3)(C)C)[C@@H](N2C(=O)c4cccc(n4)C)c(c5F)cccc5OCc6ccccc6 | 70 | 10.1016/j.bmcl.2009.03.035 |
| 233 | CN(C)[C@H](C1)CN[C@H]1C(=O)Cn2c(-c3ccccc3)c(c(c24)ccc(c4)C(=O)O)C5CCCCC5 | 71 | 10.1021/jm050056+ |
| 234 | C1COCCN1c(c2O)nn(CCC(C)C)c(=O)c2C(N3)=NS(=O)(=O)c(c34)cc(cc4)OS(=O)(=O)C | 73 | 10.1016/j.bmcl.2008.08.094 |
| 235 | c1cccc(c12)NC(=NS2(=O)=O)c(c3O)c(=O)n(c(c34)cccc4)NCc(cc5Br)ccc5 | 75 | 10.1016/j.bmcl.2005.01.071 |
| 236 | c1cc(C(=O)O)c(O)c(c12)n(C)c(-c3ccoc3)c2C4CCCC4 | 75 | 10.1016/j.bmcl.2006.07.074 |
| 237 | c1ccc(O)c(c12)NC3=C(C(=O)CC(C3)(C)C)[C@@H](N2C(=O)c4ccco4)c(c5F)cccc5OCc6ccccc6 | 75 | 10.1016/j.bmcl.2009.03.035 |
| 238 | c1ccsc1-c2nn(CCC(C)C)c(=O)c(c2O)C(=N3)NS(=O)(=O)c(c34)cc(cc4)S(=O)(=O)N | 76 | 10.1016/j.bmcl.2008.02.072 |
| 239 | CC(C)N(C)Cc(ccc1)cc1-c(n2CC(=O)N(C)C)c(c(c23)ccc(c3)C(=O)O)C4CCCCC4 | 78 | 10.1021/jm050056+ |
| 240 | c1cc(C(=O)O)cc(c12)sc(-c3ccoc3)c2C4CCCC4 | 78 | 10.1016/j.bmcl.2006.07.074 |
| 241 | O=c1[nH]cccc1-c(c(c23)cc(cc2)OCC)c(C(=O)O)n3Cc4cc(F)ccc4F | 79 | 10.1021/jm201258k |
| 242 | O=c1c(O)c(C(=O)O)nc(n1C)-c2sccc2NC(=O)Nc3ccccc3Cl | 80 | 10.1021/jm051064t |
| 243 | c1cc(C(=O)O)nc(c12)n(C)c(-c3ccccc3)c2C4CCCCC4 | 80 | 10.1016/j.bmcl.2006.07.074 |
| 244 | C=1C=C(O)CC(C12)=C(C=N2)C[C@@H](c3cscn3)NC(=O)c(cc4)cc(c45)nc(-c6cccs6)n5C7CCCCC7 | 80 | 10.1016/j.bmcl.2010.02.003 |
| 245 | C=1C=C(O)CC(C12)=C(C=N2)C[C@@H](c3cscn3)NC(=O)c(cc4)cc(c45)nc(n5C6CCCCC6)C(=O)c(cc7)cc(c78)nc(-c9cocc9)n8C1CCCC1 | 80 | 10.1016/j.bmcl.2010.02.003 |
| 246 | C1C[C@H](C)CC[C@H]1C(=O)N(C(C)C)c2c(C(=O)O)cc(cc2)Oc3c(C(F)(F)F)cccn3 | 80 | 10.1016/j.bmcl.2013.09.102 |
| 247 | O=[N+]([O-])c(c1)cccc1Cn(c(c23)cccc2)c(=O)c(c3O)C(=NS4(=O)=O)Nc(c45)cccc5 | 81 | 10.1021/jm050855s |
| 248 | c1cc(C(=O)O)cc(c12)oc(-c3ccoc3)c2C4CCCC4 | 81 | 10.1016/j.bmcl.2006.07.074 |
| 249 | c1cccc(c12)NC(=NS2(=O)=O)c(c(O)c(c34)cccn4)c(=O)n3Cc5ccccc5 | 83 | 10.1016/j.bmcl.2005.01.071 |
| 250 | c1cc(C(=O)O)ccc1Oc(cc2)cc(C(=O)O)c2NS(=O)(=O)c(cc3)ccc3C | 85 | 10.1016/j.bmcl.2013.09.102 |
| 251 | c1ccc(O)c(c12)NC3=C(C(=O)CC(C3)(C)C)[C@@H](N2C(=O)c4ccncc4)c(c5F)cccc5OCc6ccccc6 | 86 | 10.1016/j.bmcl.2009.03.035 |
| 252 | O1CC(=O)NCCCCCN(C)S(=O)(=O)NC(=O)c(cc2)cc(n3C)c2c(c3c(c14)cc(F)cc4)C5CCCCC5 | 88 | 10.1016/j.bmcl.2012.03.097 |
| 253 | C[C@@H]1CC[C@H](CC1)C(=O)N(N(C)C)c2cc(C#CC(C)(C)C)sc2C(=O)O | 88 | 10.1016/j.bmcl.2012.05.025 |
| 254 | c1ccc(F)cc1C(=O)N/C(C(=O)O)=C\c2ccc(cc2)Oc3c(Br)cccc3 | 90 | 10.1016/j.bmcl.2005.03.106 |
| 255 | c1ccc(F)c(c12)n(CCC3CC3)c(=O)c(c2O)C(=NS4(=O)=O)Nc(c45)cccc5 | 90 | 10.1021/jm050855s |
| 256 | O=C(O)c(o1)cc(c12)cc(cc2)NC(=O)[C@H](C)NC(=O)c(cc3)cc(c34)nc(-c5cocc5)n4C6CCCCC6 | 90 | 10.1016/j.bmcl.2009.10.136 |
| 257 | c1ccccc1COc2ccc(cc2)-c(c3C4CCCCC4)cnc(n35)c(cn5)C(=O)N[C@H](C(=O)O)Cc(c[nH]6)c(c67)cccc7 | 90 | 10.1016/j.bmcl.2009.09.087 |
| 258 | C1CCCCC1c(c(n23)ccc(c2)C(=O)O)c(c3)-c4ccccc4 | 94 | 10.1016/j.bmcl.2006.05.012 |
| 259 | CS(=O)(=O)Nc(cc1)cc(c12)S(=O)(=O)N=C2C(=C3O)C(=O)N([C@H]3C(C)(C)C)Cc4cc(Cl)c(F)cc4 | 94 | 10.1016/j.bmcl.2008.05.083 |
| 260 | c1ccccc1C(c2ccccc2)Oc(cc3F)ccc3-c(n4)n(c(c45)ccc(c5)C(=O)O)C6CCCCC6 | 96 | 10.1016/j.bmcl.2006.01.032 |
| 261 | c1ccccc1Cn(c(c23)nccc2)c(=O)c(c3O)C(NS4(=O)=O)=Nc(c45)c(C)ccc5 | 96 | 10.1016/j.bmcl.2006.04.022 |
| 262 | c1cccc(c12)NC(=NS2(=O)=O)c(c3O)c(=O)n(CCCC)c(c34)cccc4 | 100 | 10.1016/j.bmcl.2004.12.030 |
| 263 | C1C[C@@H](C2)C[C@H]([C@H]12)C(=O)N/C(C(=O)O)=C\c3ccc(cc3)Oc4c(Br)cccc4 | 100 | 10.1016/j.bmcl.2005.03.106 |
| 264 | C1CCCC1CCC(=O)NC(\C(=O)O)=C/c2ccc(cc2)Oc3ccccc3 | 100 | 10.1016/j.bmcl.2005.03.106 |
| 265 | C1CCCCC1n(c(c23)ccc(c2)C(=O)O)c(n3)-c4ccc(cc4)OCc5cc(Cl)ccc5-c6ccc(cc6)OC | 100 | 10.1021/jm060269e |
| 266 | C1CCCCC1c2c(-c3cocc3)n(C)c(c24)cc(cc4)C(=O)N[C@@H](c5cscn5)CC(=CN=6)[C@H](C67)C=C(O)C=C7 | 100 | 10.1016/j.bmcl.2009.10.136 |
| 267 | c1cccc(c12)n(CCCC(F)(F)F)c(=O)c(c2O)C(=NS3(=O)=O)Nc(c34)cccc4 | 106 | 10.1021/jm050855s |
| 268 | C1CCCCC1c2c(-c3cocc3)n(C)c(c24)cc(cc4)C(=O)NC(C)(C)C(=O)Nc5ccc(cc5)/C=C/C(=O)O | 106 | 10.1016/j.bmcl.2011.04.059 |
| 269 | O=S(=O)(C)N(C)c(cc1)cc(c12)S(=O)(=O)C=C(N2)c(c3O)c(=O)n(c(c34)ccc(F)c4)Cc5ccc(F)cc5 | 106 | 10.1016/j.bmcl.2009.05.004 |
| 270 | C1CCCCC1c2c(-c3cccnc3)n(C)c(c24)cc(cc4)C(=O)NC(C)(C)C(=O)Nc5ccc(cc5)/C=C/C(=O)O | 109 | 10.1016/j.bmcl.2011.04.059 |
| 271 | CCC[C@@](CC(=O)O)(OCC1)C(=C12)N=C3[C@@H]2C(C#N)=CC(=C3C)OCC | 110 | 10.1016/j.bmcl.2011.04.052 |
| 272 | C1CCCCC1c2c(-c3ccncc3)n(C)c(c24)cc(cc4)C(=O)NC(C)(C)C(=O)Nc5ccc(cc5)/C=C/C(=O)O | 110 | 10.1016/j.bmcl.2011.04.059 |
| 273 | O=C(C1)NCCCCN(C)S(=O)(=O)NC(=O)c(c2)ccc(c2n13)c(c3-c4cocc4)C5CCCCC5 | 110 | 10.1016/j.bmcl.2012.03.097 |
| 274 | O=C1NS(=O)(=O)N(C)CCOCCN(C)C(=O)COc(cccc2)c2c(n(C)c(c3)c4ccc13)c4C5CCCCC5 | 110 | 10.1016/j.bmcl.2012.03.097 |
| 275 | Fc1cc(F)cc(F)c1Cn2cnc(=O)c(c23)cc(cc3)Oc4c(C(F)(F)F)ccnc4 | 110 | 10.1016/j.bmcl.2013.05.037 |
| 276 | O=C(O)CC[C@H](C(=O)O)NC(=O)c1c(Br)ccc(c12)c(C(F)(F)F)c(cc2)OC | 120 | 10.1016/j.bmcl.2004.06.013 |
| 277 | c1cc(Br)ccc1C(=O)N/C(C(=O)O)=C\c2ccc(cc2)Oc3c(Br)cccc3 | 120 | 10.1016/j.bmcl.2005.03.106 |
| 278 | c1ccccc1-c([nH]c(c23)sc(c3)C(=O)O)c2C4CCCCC4 | 120 | 10.1016/j.bmcl.2006.05.012 |
| 279 | OC(=O)c(c1)ccc(c12)c(C3CCCCC3)c4n2CCN(C(=O)C)c5c4cccc5 | 120 | 10.1021/jm0610245 |
| 280 | c1cc(F)cnc1CN([C@@H]([C@@H]23)CCC3)C(=O)C(=C2O)C(N4)=NS(=O)(=O)c(c45)cc(cc5)NS(=O)(=O)C | 120 | 10.1016/j.bmcl.2008.11.048 |
| 281 | OC(=O)/C=C\c1ccc(cc1)NC(=O)C2(CCC2)NC(=O)c(cc3)cc(c34)nc(-c5ccsc5)n4C6CCCCC6 | 120 | 10.1016/j.bmcl.2010.02.003 |
| 282 | c1ccc(O)c(c12)NC3=C(C(=O)CC(C3)(C)C)[C@@H](N2C(=O)C)c(c(Cl)c4)ccc4Oc5c(F)cccc5Br | 120 | 10.1016/j.bmcl.2009.03.035 |
| 283 | Fc1cc(F)ccc1Cn2cnc(=O)c(c23)cc(cc3)Oc4c(C(F)(F)F)nccc4 | 120 | 10.1016/j.bmcl.2013.05.037 |
| 284 | c1ccnc(c12)n(CCC(C)C)c(=O)c(c2O)C(=NS3(=O)=O)Nc(c34)ccc(c4)NS(=O)(=O)N(C)C(=O)OCc5ccccc5 | 121 | 10.1016/j.bmcl.2006.04.015 |
| 285 | c1ccc(O)c(c12)NC3=C(C(=O)CC(C3)(C)C)[C@@H](N2C(=O)C(C)C)c(c4F)cccc4OCc5ccccc5 | 123 | 10.1016/j.bmcl.2009.03.035 |
| 286 | C1CCCCC1c2c(-c3ccccn3)n(C)c(c24)cc(cc4)C(=O)NC(C)(C)C(=O)Nc5ccc(cc5)NC(=O)C(=O)N | 124 | 10.1016/j.bmcl.2011.04.082 |
| 287 | c1cccc(c12)NC(=NS2(=O)=O)c(c3O)c(=O)n(c(c34)cccc4)NCc5sccc5 | 125 | 10.1016/j.bmcl.2005.01.071 |
| 288 | c1ccnc(c12)n(CCC(C)C)c(=O)c(c2O)C(=NS3(=O)=O)Nc(c34)ccc(c4)NS(=O)(=O)Nc5ccccc5C(=O)OC | 129 | 10.1016/j.bmcl.2006.04.015 |
| 289 | n1c(N)nc(O)c(c12)ncn2[C@@H]([C@@]3(O)C)O[C@@H]([C@H]3O)CO | 130 | 10.1021/jm030424e |
| 290 | COc1ccc(cc1Cl)CC[C@@]2(C3CCCC3)OC(O)=C(C(=O)C2)Sc4ccc(O)cc4 | 130 | 10.1016/j.bmcl.2006.06.065 |
| 291 | c1cc(F)ccc1Cn2c(=O)c(c(O)c(n23)ccc3)C(N4)=NS(=O)(=O)c(c45)cc(cc5)[C@@H](S6(=O)=O)CCC6 | 130 | 10.1016/j.bmcl.2008.04.066 |
| 292 | c1cc(F)c(Cl)cc1CN2C(=O)C(=C(O)C23CC3)C4=NS(=O)(=O)c(c45)c(ccc5)CNS(=O)(=O)C | 130 | 10.1016/j.bmcl.2008.05.083 |
| 293 | c1cnccc1-c(n2CC(=O)N(C)C)c(c(c23)ccc(c3)C(=O)O)C4CCCCC4 | 134 | 10.1021/jm050056+ |
| 294 | CC(C)(C)C#Cc(sc1C(=O)O)cc1N(C(=O)[C@@H](CC2)CC[C@H]2C)N(C)C3CCNCC3 | 138 | 10.1016/j.bmcl.2012.05.025 |
| 295 | Clc1ccc(Cl)c(c12)sc3c2CCO[C@]3(CCC)CC(=O)O | 140 | 10.1016/j.bmcl.2005.08.114 |
| 296 | OC(=O)c(c1)ccc(c12)c(C3CCCCC3)c4n2CCc5c4cccc5 | 140 | 10.1021/jm0610245 |
| 297 | c1ccc(O)c(c12)NC3=C(C(=O)CC(C3)(C)C)[C@@H](N2C(=O)c4nc(C)sc4)c(c5F)cccc5OCc6ccccc6 | 143 | 10.1016/j.bmcl.2009.03.035 |
| 298 | c1cccc(c12)NC(=NS2(=O)=O)c(c3O)c(=O)n(c(c34)cccc4)NCC5CC5 | 145 | 10.1016/j.bmcl.2005.01.071 |
| 299 | c1cccc(c12)NC(=NS2(=O)=O)C3=C(O)[C@H](C(C)C)N(C3=O)CCC(C)C | 147 | 10.1016/j.bmcl.2006.01.034 |
| 300 | c1cccc(c12)NC(=NS2(=O)=O)c(c3O)c(=O)n(c(c34)cccc4)NCC5CCCC5 | 150 | 10.1016/j.bmcl.2005.01.071 |
| 301 | c1cccc(c12)n(C/C=C/C)c(=O)c(c2O)C(=NS3(=O)=O)Nc(c34)cccc4 | 150 | 10.1021/jm050855s |
| 302 | C1CCCCC1c(c(c23)ccc(n2)C(=O)O)c([nH]3)-c4ccccc4 | 150 | 10.1016/j.bmcl.2006.05.012 |
| 303 | s1cccc1-c(c2O)nn(Cc3ccccc3)c(=O)c2C(N4)=NS(=O)(=O)c(c45)cc(cc5)OCC(=O)N | 150 | 10.1016/j.bmcl.2008.01.007 |
| 304 | CC[C@H](C)[C@@](CC(=O)O)(OCC1)C(=C12)N=C3[C@@H]2C(Cl)=CC=C3Cl | 150 | 10.1016/j.bmcl.2010.03.002 |
| 305 | CS(=O)(=O)Nc(c1)ccc(c12)NC(=NS2(=O)=O)C(=C3O)C(=O)N(C4CC4)C[C@@]3(C)CCC(C)(C)C | 150 | 10.1016/j.bmcl.2009.09.051 |
| 306 | C1CCCCC1c2c(-c3ccc(cn3)C(F)(F)F)n(C)c(c24)cc(cc4)C(=O)NC(C)(C)C(=O)Nc5ccc(cc5)/C=C/C(=O)O | 157 | 10.1016/j.bmcl.2011.04.059 |
| 307 | c1cocc1-c(n2)n(C3CCCCC3)c(c24)ccc(c4)C(=O)N[C@@H](C(=O)O)Cc5cn(C)c(c56)ccc(c6)O | 160 | 10.1016/j.bmcl.2003.12.032 |
| 308 | N#Cc1ccc(C)c(c12)sc3c2CCO[C@]3(CCC)CC(=O)O | 170 | 10.1016/j.bmcl.2005.08.114 |
| 309 | O=S(=O)(C)Nc(cc1)cc(c12)S(=O)(=O)NC(=N2)c(c3O)c(=O)n(CCC(C)C)nc3CCC(F)(F)F | 170 | 10.1016/j.bmcl.2008.02.072 |
| 310 | O=C(O)\C=C\c(cc1)ccc1NC(=O)C(C)(C)NC(=O)C(=CC=2)CC(C23)=NC(c4cocc4)=C3C5CCCCC5 | 172 | 10.1016/j.bmcl.2011.04.059 |
| 311 | c1cc(Br)cc(c12)S(=O)(=O)N=C(N2)c(c3O)c(=O)n(CCC(C)C)c(c34)cccc4 | 173 | 10.1016/j.bmcl.2009.05.091 |
| 312 | C1CCCCC1c2c(-c3ccc(N)nc3)n(C)c(c24)cc(cc4)C(=O)NC(C)(C)C(=O)Nc5ccc(cc5)/C=C/C(=O)O | 177 | 10.1016/j.bmcl.2011.04.059 |
| 313 | c1cc(C(=O)O)cc(c12)c(C)c(-c3ccccc3)n2C4CCCCC4 | 180 | 10.1016/j.bmcl.2006.07.074 |
| 314 | C1CCN([C@@]12C)N(CCC(C)(C)C)C(=O)C(=C2O)C(N3)=NS(=O)(=O)c(c34)cc(cc4)NS(=O)(=O)C | 180 | 10.1016/j.bmcl.2008.08.017 |
| 315 | C1CCCCC1c2c(-c(cc3)ccc3OC)n(c4c25)CC(=O)NCCCCN(C)S(=O)(=O)NC(=O)c(c4)cc5 | 180 | 10.1016/j.bmcl.2012.03.097 |
| 316 | c1ccnc(c12)n(CCC(C)C)c(=O)c(c2O)C(=NS3(=O)=O)Nc(c34)ccc(c4)NS(=O)(=O)NC(=O)OCCCl | 189 | 10.1016/j.bmcl.2006.04.015 |
| 317 | C1C[C@H](C)CC[C@H]1C(=O)N(C(C)C)c2c(C(=O)O)cc(cc2)Oc3ccccc3C(F)(F)F | 190 | 10.1016/j.bmcl.2013.09.102 |
| 318 | n1nn[nH]c1-c(cn2)c(n23)ncc(c3C4CCCCC4)-c(cc5)ccc5OCc(cc6)ccc6C(=O)OC | 190 | 10.1016/j.bmcl.2009.09.087 |
| 319 | c1ccccc1C(=O)N/C(C(=O)O)=C\c2ccc(cc2)Oc3c(Cl)cccc3C | 200 | 10.1016/j.bmcl.2005.03.066 |
| 320 | C1CC(C)(C)Cc(c12)sc(c2C(=O)OCC)NC(=O)NS(=O)(=O)N3CCc(c34)cccc4 | 200 | 10.1016/j.bmcl.2005.09.047 |
| 321 | c1c(Cl)c(Cl)ccc1/C=C2\SC(=S)N(C2=O)NS(=O)(=O)c3ccccc3 | 200 | 10.1021/jm050859x |
| 322 | c1cc(Cl)c(Cl)cc1/C=C2\SC(=S)N(C2=O)NS(=O)(=O)c3sccc3 | 200 | 10.1021/jm050859x |
| 323 | c1cc(Cl)c(Cl)cc1/C=C2\SC(=S)N([C@@H]2O)NS(=O)(=O)c3ccccc3F | 200 | 10.1021/jm050859x |
| 324 | c1c(F)c(F)cc(F)c1/C=C2\SC(=S)N(C2=O)NS(=O)(=O)c3ccccc3 | 200 | 10.1021/jm050859x |
| 325 | C1C[C@H](C)CC[C@H]1C(=O)N(C(C)C)c2c(C(=O)O)cc(cc2)Oc3ccccc3C(F)F | 200 | 10.1016/j.bmcl.2013.09.102 |
| 326 | Fc1cccc(F)c1Cn2cnc(=O)c(c23)cc(cc3)Oc4ncccc4C(F)(F)F | 200 | 10.1016/j.bmcl.2013.05.037 |
| 327 | n1nn[nH]c1-c(cn2)c(n23)ncc(c3C4CCCCC4)-c(cc5)ccc5Oc(c6)cccc6S(=O)(=O)C | 200 | 10.1016/j.bmcl.2009.09.087 |
| 328 | O=C(O)c(n1)c(O)c(O)nc1-c2sccn2 | 210 | 10.1021/jm051064t |
| 329 | C1CCCC1Oc(cc2)cc(C(=O)O)c2NS(=O)(=O)c(cc3)ccc3C | 210 | 10.1016/j.bmcl.2013.09.102 |
| 330 | c1cccc(c12)NC(=NS2(=O)=O)c(c3O)c(=O)n(c(c34)cccc4)NCC5CCCCCC5 | 217 | 10.1016/j.bmcl.2005.01.071 |
| 331 | c1ccccc1Oc(cc2)cc(C(=O)O)c2NS(=O)(=O)c(cc3)ccc3C | 220 | 10.1016/j.bmcl.2013.09.102 |
| 332 | OC(=O)/C=C/c1ccc(cc1)NC(=O)C2(CCCC2)NC(=O)c(cc3)cc(c34)n5c(C=6[C@H](N=CN6)CCC5)c4C7CCCCC7 | 228 | 10.1016/j.bmcl.2012.02.063 |
| 333 | c1ccccc1Cn(c(c23)nccc2)c(=O)c(c3O)C(NS4(=O)=O)=Nc(c45)ccc(c5)C | 230 | 10.1016/j.bmcl.2006.04.022 |
| 334 | c1ccccc1-c(n(CC(=O)N(C)C)c(c23)sc(c3)C(=O)O)c2C4CCCCC4 | 230 | 10.1016/j.bmcl.2006.05.012 |
| 335 | c1cc(C(=O)O)cc(c12)[nH]c(-c3ccccn3)c2C4CCCCC4 | 230 | 10.1016/j.bmcl.2006.07.074 |
| 336 | C=1C=C(O)CC(C12)=C(C=N2)C[C@@H](c3cscn3)NC(=O)c(cc4)cc(c45)nc(-c6ccccc6)n5C7CCCCC7 | 230 | 10.1016/j.bmcl.2010.02.003 |
| 337 | Fc1cc(F)ccc1Cn2cnc(=O)c(c23)cc(cc3)Oc4c(C(F)(F)F)c(ccn4)OC | 230 | 10.1016/j.bmcl.2013.05.037 |
| 338 | CC(C)C[C@@]1(C(=O)O)C[C@H](C(=O)NS(=O)(=O)C)[C@H](c2cccs2)N1C(=O)c3ccc(C(F)(F)F)cc3 | 230 | 10.1016/j.bmcl.2007.01.034 |
| 339 | c1cccc(c12)C(CCC)(CCC)C(=O)C(=C2O)C(=NS3(=O)=O)Nc(c34)ccc(c4)OCC(=O)N | 240 | 10.1016/j.bmcl.2008.06.043 |
| 340 | O=C(O)[C@@H]1CSCN1S(=O)(=O)c2c(O)c(Cl)cc(Cl)c2 | 240 | 10.1021/jm060168g |
| 341 | CC(C)(C)CCN([C@H]1C(C)(C)C)C(=O)C(=C1O)C2=CS(=O)(=O)c(c23)c(ccc3)CNS(=O)(=O)C | 240 | 10.1016/j.bmcl.2008.05.083 |
| 342 | Fc1cc(F)ccc1Cn2cnc(=O)c(c23)cc(c(F)c3)Oc4ncccc4C(F)(F)F | 240 | 10.1016/j.bmcl.2013.05.037 |
| 343 | c1ccccc1-c2nn(CCC(C)C)c(=O)c(c2O)C(N3)=CS(=O)(=O)c(c34)cc(cc4)NS(=O)(=O)C | 250 | 10.1016/j.bmcl.2008.07.014 |
| 344 | Nc(n1)sc(c12)CCCn3c2c(C4CCCCC4)c5c3cc(cc5)C(=O)NC6(CCCC6)C(=O)Nc7ccc(cc7)/C=C/C(=O)O | 250 | 10.1016/j.bmcl.2012.02.063 |
| 345 | C1=CC(O)=C[C@@H](C12)C(=CN=2)C[C@@H](C(=O)O)NC(=O)c(cc3)cc(c34)nc(-c5occc5)n4C6CCCCC6 | 270 | 10.1016/j.bmcl.2010.02.003 |
| 346 | Oc1ccn(CCC(C)C)c(=O)c1C(=NS2(=O)=O)Nc(c23)ccc(c3)NS(=O)(=O)C | 274 | 10.1016/j.bmcl.2004.12.030 |
| 347 | Oc1c(C(=O)C)cn(CCC(C)C)c(=O)c1C(=NS2(=O)=O)Nc(c23)ccc(c3)NS(=O)(=O)C | 280 | 10.1016/j.bmcl.2004.12.030 |
| 348 | O=C(O)c(n1)c(O)c(O)nc1-c2sccc2NC(=O)Cc3c([nH]c(c34)cccc4)-c5ccccc5 | 280 | 10.1021/jm051064t |
| 349 | COc1ccc(cc1Cl)CC[C@@]2(C3CCCC3)OC(O)=C(C(=O)C2)Sc4n(C)nnn4 | 280 | 10.1016/j.bmcl.2006.06.065 |
| 350 | OC(=O)/C=C/c1ccc(cc1)NC(=O)CNC(=O)c(cc2)cc(c23)nc(-c4cocc4)n3C5CCCCC5 | 280 | 10.1016/j.bmcl.2009.10.136 |
| 351 | O=S(=O)(C)Nc(cc1)cc(c12)S(=O)(=O)NC(=N2)c(c3O)c(=O)n(CCC(C)C)nc3CCC(C)C | 280 | 10.1016/j.bmcl.2008.02.072 |
| 352 | O=C(C)Nc(cc1)ccc1Oc(cc2)cc(C(=O)O)c2NS(=O)(=O)c(cc3)ccc3C | 290 | 10.1016/j.bmcl.2013.09.102 |
| 353 | c1ccsc1-c2nn(CCC(C)C)c(=O)c(c2O)C(=N3)NS(=O)(=O)c(c34)cc(cc4)N5CCCS5(=O)=O | 290 | 10.1016/j.bmcl.2008.02.072 |
| 354 | C1CCCCC1c2c(-c(cc3)ccc3F)cnc(n24)c(cn4)C(=O)N[C@H](C(=O)O)C(C)C | 290 | 10.1016/j.bmcl.2009.09.087 |
| 355 | C1CCCCC1c2c(-c(cc3Cl)ccc3C)n(C)c(c24)cc(cc4)C(=O)NC(C)(C)C(=O)Nc5ccc(cc5)/C=C/C(=O)O | 298 | 10.1016/j.bmcl.2011.04.059 |
| 356 | O=C(O)c(c1)ccc(c12)n(C3CCCCC3)c(n2)-c4ccc(cc4)OCc5ccccc5-c6ccccc6 | 300 | 10.1016/j.bmcl.2006.01.032 |
| 357 | CCC[C@](CC(=O)O)(OCC1)C(=C12)N=C3[C@@H]2C(C#N)=CC=C3C | 300 | 10.1016/j.bmcl.2010.03.002 |
| 358 | Fc1cc(F)cc(c1)Nc2c(C#N)c(ns2)O | 300 | 10.1016/j.bmcl.2006.10.002 |
| 359 | s1nc(O)c(C#N)c1Nc(c2)c(ccc2Br)OCc3ccccc3 | 300 | 10.1016/j.bmcl.2006.10.002 |
| 360 | c1ccccc1C(=O)N/C(C(=O)O)=C\c2ccc(cc2)Oc3c(N)cccc3Cl | 310 | 10.1016/j.bmcl.2005.03.066 |
| 361 | Brc1cc(F)c(cc1)S(=O)(=O)Nc2c(C(=O)O)ccc(c2)Oc3ccccc3 | 310 | 10.1016/j.bmcl.2013.09.102 |
| 362 | C1CCCCC1c2c(-c3ccccn3)n(C)c(c24)cc(cc4)C(=O)NC(C)(C)C(=O)Nc5ccc(cc5)-c6oc(cc6)C(=O)N | 330 | 10.1016/j.bmcl.2011.04.082 |
| 363 | CC(C)C[C@@]1(C(=O)O)C[C@H](C(=O)N)[C@H](c2cccs2)N1C(=O)c3ccc(cc3)C(C)(C)C | 330 | 10.1016/j.bmcl.2007.01.034 |
| 364 | c1ccncc1-c(n2CC(=O)N(C)C)c(c(c23)ccc(c3)C(=O)O)C4CCCCC4 | 332 | 10.1021/jm050056+ |
| 365 | c1cc(O)ccc1-c(c2)n(c(c23)ccc(c3)C(=O)O)C4CCCCC4 | 340 | 10.1021/jm049122i |
| 366 | C1CCCCC1c2c(-c3ccccc3)n(C)c(c24)cc(cc4)C(=O)NC(C)(C)C(=O)Nc5ccc(cc5)/C=C/C(=O)O | 345 | 10.1016/j.bmcl.2011.04.059 |
| 367 | C1CCCCC1n(c(c23)ccc(c2)C(=O)O)c(n3)-c4ccc(cc4Cl)OCc5ccccc5-c6ccccc6 | 350 | 10.1021/jm060269e |
| 368 | C1CCC1[C@](CC(=O)O)(OCC2)C(=C23)N=C4[C@@H]3C(Cl)=CC=C4Cl | 350 | 10.1016/j.bmcl.2010.03.002 |
| 369 | C1CCCCC1Cn(c(c23)cccc2)c(=O)c(c3O)C(=NS4(=O)=O)Nc(c45)cccc5 | 367 | 10.1021/jm050855s |
| 370 | FC(F)(F)c1ccc(cc1)CCC(=O)NC(\C(=O)O)=C/c2ccc(cc2)Oc3c(Br)cccc3 | 370 | 10.1016/j.bmcl.2005.03.106 |
| 371 | O=C(O)C(=O)/C=C(O)/c1c(cccc1)OCCCC#N | 380 | 10.1021/jm0504454 |
| 372 | O=C(O)c(c1)ccc(c12)n(C3CCCCC3)c(n2)-c4ccc(cc4)OCc5cccc(c5)-c6ccccc6 | 390 | 10.1016/j.bmcl.2006.01.032 |
| 373 | c1cc(C(=O)O)ccc1NC(=O)[C@H](C)NC(=O)c(cc2)cc(c23)nc(-c4cocc4)n3C5CCCCC5 | 390 | 10.1016/j.bmcl.2009.10.136 |
| 374 | c1cc(C(=O)O)cc(c12)n3c(c4c(OCC3)cccn4)c2C5CCCCC5 | 390 | 10.1039/c1ob05525a |
| 375 | c1cocc1-c(n2)n(C3CCCCC3)c(c24)ccc(c4)C(=O)N[C@H](C(=O)O)Cc5c[nH]c(c56)ccc(c6)OC | 400 | 10.1016/j.bmcl.2003.12.032 |
| 376 | s1ccnc1[C@@H]([C@H](C2)C(=O)O)N([C@@]2(C(=O)O)CC(C)C)C(=O)c3ccc(C(F)(F)F)cc3 | 400 | 10.1016/j.bmcl.2005.01.076 |
| 377 | C1CC(C)(C)Cc(c12)sc(c2C(=O)OCC)NC(=O)NS(=O)(=O)N3CCN(C)CC3 | 400 | 10.1016/j.bmcl.2005.09.047 |
| 378 | N#CC1=CC=C(C)[C@H]2N=C(C3=C12)[C@](OCC3)(CC(=O)O)C4CCC4 | 400 | 10.1016/j.bmcl.2010.03.002 |
| 379 | n1cccc(c12)cc(cc2)-c(c3C4CCCCC4)n(C)c(c35)cc(cc5)C(=O)NC(C)(C)C(=O)Nc6ccc(cc6)/C=C/C(=O)O | 400 | 10.1016/j.bmcl.2011.04.059 |
| 380 | O=c1[nH]cccc1-c2c(C(=O)NS(=O)(=O)C(C)C)n(c(c23)ccc(Cl)c3)Cc4ccccc4F | 400 | 10.1016/j.bmcl.2011.10.041 |
| 381 | n1nn[nH]c1-c(cn2)c(n23)ncc(c3C4CCCCC4)-c(cc5)ccc5Oc(c6)cccc6OC | 400 | 10.1016/j.bmcl.2009.09.087 |
| 382 | s1cccc1-c2nn(CCC(C)C)c(=O)c(c2O)C(N3)=NS(=O)(=O)c(c34)cc(cc4)NS(=O)(=O)C | <10 | 10.1016/j.bmcl.2008.04.066 |
| 383 | CC(C)CCn1c(=O)c(c(O)c(n12)ccc2)C(=N3)NS(=O)(=O)c(c34)cc(cc4)NS(=O)(=O)C | <10 | 10.1016/j.bmcl.2008.04.066 |
| 384 | CC(C)(C)CCn1c(=O)c(c(O)c(n12)ccc2)C(N3)=NS(=O)(=O)c(c34)cc(cc4)NS(=O)(=O)C | <10 | 10.1016/j.bmcl.2008.04.066 |
| 385 | s1cccc1-c2nn(CCC(C)C)c(=O)c(c2O)C(=N3)CS(=O)(=O)c(c34)cc(cc4)NS(=O)(=O)C | <10 | 10.1016/j.bmcl.2008.07.014 |
| 386 | CS(=O)(=O)Nc(cc1)cc(c12)S(=O)(=O)N=C(N2)C(=C3O)C(=O)N(N([C@H]34)CCC4)Cc5ccc(F)cc5 | <10 | 10.1016/j.bmcl.2008.08.017 |
| 387 | C1CC[C@H]([C@H]12)N(CCC(C)(C)C)C(=O)C(=C2O)C(N3)=NS(=O)(=O)c(c34)cc(cc4)NS(=O)(=O)C | <10 | 10.1016/j.bmcl.2008.11.048 |
| 388 | c1cc(F)ccc1CN([C@H]([C@H]23)CCC3)C(=O)C(=C2O)C(N4)=NS(=O)(=O)c(c45)cc(cc5)NS(=O)(=O)C | <10 | 10.1016/j.bmcl.2008.11.048 |
| 389 | c1c(Br)cccc1N(C(=O)c2ccc3)C(=O)c(c2c34)ccc4NC5CCN(CC5)Cc6ccccc6 | <14 | 10.1021/jm900517t |
| 390 | n1c(N)nc(O)c(c12)ncn2[C@@H]3O[C@H](CO)C[C@H]3O | 600 | 10.1021/jm030424e |
| 391 | Fc1ccc(cc1)CCC(=O)NC(\C(=O)O)=C/c2ccc(cc2)Oc3c(Br)cccc3 | 600 | 10.1016/j.bmcl.2005.03.106 |
| 392 | Nc(c1)nccc1Cn(c(c23)ccc(Cl)c3)c(C(=O)NS(=O)(=O)CC)c2-c4ccc[nH]c4=O | 600 | 10.1016/j.bmcl.2011.10.041 |
| 393 | O=C(O)c(n1)c(O)c(O)nc1-c2sccc2NC(=O)NCCc3ccccc3 | 620 | 10.1021/jm051064t |
| 394 | c1cc(C(F)(F)F)ccc1CN([C@H]([C@H]23)CCC3)C(=O)C(=C2O)C(N4)=NS(=O)(=O)c(c45)cc(cc5)NS(=O)(=O)C | 620 | 10.1016/j.bmcl.2008.11.048 |
| 395 | C1CCCCC1c2c(-c(cc3C)cnc3N)n(C)c(c24)cc(cc4)C(=O)NC(C)(C)C(=O)Nc5ccc(cc5)/C=C/C(=O)O | 628 | 10.1016/j.bmcl.2011.04.059 |
| 396 | c1cccc(c12)NC(=NS2(=O)=O)c(c3O)c(=O)n(NC(C)C)c(c34)cccc4 | 640 | 10.1016/j.bmcl.2005.01.071 |
| 397 | c1cccc(Cl)c1-c2sc(cc2)/C=C(C(=O)O)\NC(=O)c3ccccc3 | 660 | 10.1016/j.bmcl.2005.03.066 |
| 398 | OC(=O)c(cc1)cc(c12)n3c(c4c(OCC3)cccc4)c2C5CCCCC5 | 690 | 10.1016/j.bmcl.2011.03.067 |
| 399 | Cc(n1)sc(c12)ccc(c2)-c(c3C4CCCCC4)n(C)c(c35)cc(cc5)C(=O)NC(C)(C)C(=O)Nc6ccc(cc6)/C=C/C(=O)O | 690 | 10.1016/j.bmcl.2011.04.059 |
| 400 | c1c(F)ccc(c12)n(CC(=O)C(C)(C)C)c(=O)c(c2O)C(=NS3(=O)=O)Nc(c34)cccc4 | 696 | 10.1021/jm050855s |
| 401 | c1cocc1-c(n2)n(C3CCCCC3)c(c24)ccc(c4)C(=O)N[C@@H](C(=O)O)Cc5c[nH]c(c56)ccc(c6)O | 700 | 10.1016/j.bmcl.2003.12.032 |
| 402 | c1ccsc1[C@@H]([C@H](C2)C(=O)O)N([C@@]2(C(=O)O)CC(C)C)C(=O)c3cccc(c34)cccc4 | 700 | 10.1016/j.bmcl.2005.01.076 |
| 403 | c1ccccc1[C@@H]([C@H](C2)C(=O)O)N([C@@]2(C(=O)O)CC(C)C)C(=O)c3ccc(C(F)(F)F)cc3 | 700 | 10.1016/j.bmcl.2005.01.076 |
| 404 | CCC[C@](C(=O)O)(OCC1)C(=C12)N=C3[C@@H]2C(C#N)=CC=C3C | 700 | 10.1016/j.bmcl.2010.03.002 |
| 405 | C1CCCCC1N([C@@H]([C@@H]23)CCC3)C(=O)C(=C2O)C(N4)=NS(=O)(=O)c(c45)cc(cc5)NS(=O)(=O)C | 730 | 10.1016/j.bmcl.2008.11.048 |
| 406 | Cn1cccc1-c(n2)n(C3CCCCC3)c(c24)ccc(c4)C(=O)N[C@@H](C(=O)O)CC(=CN=5)[C@H](C56)C=C(O)C=C6 | 730 | 10.1016/j.bmcl.2010.02.003 |
| 407 | C1CCCCC1c2c(-c3ccccn3)n(C)c(c24)cc(cc4)C(=O)NC5(CCC5)C(=O)Nc6ccc(cc6)-c7cnc(N)nc7 | 745 | 10.1016/j.bmcl.2011.04.082 |
| 408 | N#CCCCCn(c(c12)cccc1)c(=O)c(c2O)C(=NS3(=O)=O)Nc(c34)cccc4 | 750 | 10.1021/jm050855s |
| 409 | c1ccc(F)cc1C(c2cccc(F)c2)Oc(cc3)ccc3-c(n4)n(c(c45)ccc(c5)C(=O)O)C6CCCCC6 | 760 | 10.1016/j.bmcl.2006.01.032 |
| 410 | CCCC[C@@](CC(=O)O)(OCC1)C(=C12)N=C3[C@@H]2C(Cl)=CC=C3Cl | 760 | 10.1016/j.bmcl.2010.03.002 |
| 411 | O=C(O)[C@@H]1CCCN1S(=O)(=O)c2c(O)c(Cl)cc(Cl)c2 | 770 | 10.1021/jm060168g |
| 412 | C1CC[C@H]([C@H]12)N(C(C)C)C(=O)C(=C2O)C(N3)=NS(=O)(=O)c(c34)cc(cc4)NS(=O)(=O)C | 780 | 10.1016/j.bmcl.2008.11.048 |
| 413 | O=C(O)c1c(O)c(=O)cc(o1)COCC | 800 | 10.1016/j.bmcl.2004.03.087 |
| 414 | c1ccoc1[C@@H]([C@H](C2)C(=O)O)N([C@@]2(C(=O)O)CC(C)C)C(=O)c3ccc(C(F)(F)F)cc3 | 800 | 10.1016/j.bmcl.2005.01.076 |
| 415 | c1ccccc1C(=O)NC(\C(=O)NOC)=C/c2ccc(cc2)Oc3c(Br)cccc3 | 800 | 10.1016/j.bmcl.2005.03.106 |
| 416 | c1cc(Cl)c(Cl)cc1/C=C2\SC(=S)N([C@@H]2O)NS(=O)(=O)c3cccc(c3)OC | 800 | 10.1021/jm050859x |
| 417 | c1cc(F)ccc1C(c2ccc(F)cc2)Oc(cc3)ccc3-c(n4)n(c(c45)ccc(c5)C(=O)O)C6CCCCC6 | 800 | 10.1016/j.bmcl.2006.01.032 |
| 418 | c1cc(Br)ccc1CC(=O)N/C(C(=O)O)=C\c2ccc(cc2)Oc3c(Br)cccc3 | 820 | 10.1016/j.bmcl.2005.03.106 |
| 419 | O=C(O)CCCC(=O)NC(\C(=O)O)=C/c1ccc(cc1)Oc2c(I)cccc2 | 830 | 10.1016/j.bmcl.2005.03.106 |
| 420 | C1CCCC1CC(=O)N/C(C(=O)O)=C\c2ccc(cc2)Oc3c(Br)cccc3 | 850 | 10.1016/j.bmcl.2005.03.106 |
| 421 | COc1ccc(cc1F)CC[C@]2(OC(=O)CC(=O)C2)C3CCCC3 | 890 | 10.1016/j.bmcl.2006.06.065 |
| 422 | c1cc(Cl)ccc1[C@@H]([C@H](C2)C(=O)O)N([C@@]2(C(=O)O)CC(C)C)C(=O)c3ccc(C(F)(F)F)cc3 | 900 | 10.1016/j.bmcl.2005.01.076 |
| 423 | C1CCCc(c12)sc(c2C(=O)OCC)NC(=O)NS(=O)(=O)c3c(C)cccc3 | 900 | 10.1016/j.bmcl.2005.09.047 |
| 424 | c1cc(C#N)ccc1/C=C2\SC(=S)N(C2=O)NS(=O)(=O)c3ccccc3 | 900 | 10.1021/jm050859x |
| 425 | O=C(O)C[C@@H](C(=O)O)NC(=O)c1c(Br)ccc(c12)c(C(F)(F)F)c(cc2)OC | 910 | 10.1016/j.bmcl.2004.06.013 |
| 426 | c1c(C)c(N)cc(C(C)(C)C)c1SC(C(=O)C2)=C(O)O[C@@]2(C3CCCC3)CCc4ccc(O)cc4 | 930 | 10.1016/j.bmcl.2006.06.065 |
| 427 | C1CCCCC1n(c(c23)ccc(c2)C(=O)O)c(n3)-c4ccc(cc4C(F)(F)F)OCc5ccccc5-c6ccccc6 | 930 | 10.1021/jm060269e |
| 428 | c1cc(Cl)ccc1CC(=O)N/C(C(=O)O)=C\c2ccc(cc2)Oc3c(Br)cccc3 | 940 | 10.1016/j.bmcl.2005.03.106 |
| 429 | O=C(O)C(=O)/C=C(O)/c1cc(ccc1)OCc(ccs2)c2C(=O)O | 950 | 10.1021/jm0504454 |
| 430 | C1CC[C@H]([C@H]12)N(CC)C(=O)C(=C2O)C(N3)=NS(=O)(=O)c(c34)cc(cc4)NS(=O)(=O)C | 950 | 10.1016/j.bmcl.2008.11.048 |
| 431 | O=C(O)c(c1)ccc(c12)n(C3CCCCC3)c(n2)-c4ccc(cc4)OCC(c5ccccc5)c6ccccc6 | 980 | 10.1016/j.bmcl.2006.01.032 |
| 432 | CC(C)CCn1c(=O)c(c(O)c(n12)ccc2)C(N3)=NS(=O)(=O)c(c34)cccc4 | 980 | 10.1016/j.bmcl.2008.04.066 |
| 433 | O=C(O)c1nc(nc(O)c1O)-c2cc(ccc2)OCc3ccccc3 | 1000 | 10.1021/jm0494669 |
| 434 | c1cc(C)ccc1[C@@H]([C@H](C2)C(=O)O)N([C@@]2(C(=O)O)CC(C)C)C(=O)c3ccc(C(F)(F)F)cc3 | 1000 | 10.1016/j.bmcl.2005.01.076 |
| 435 | FC(F)(F)c1ccc(cc1)CC(=O)N/C(C(=O)O)=C\c2ccc(cc2)Oc3c(Br)cccc3 | 1000 | 10.1016/j.bmcl.2005.03.106 |
| 436 | O=C(O)C(=O)/C=C(O)/c1cc(O)ccc1 | 1000 | 10.1021/jm0504454 |
| 437 | C1CCCc(c12)sc(c2C(=O)OCC)NC(=O)NS(=O)(=O)c3ccc(Cl)cc3 | 1000 | 10.1016/j.bmcl.2005.09.047 |
| 438 | C1CCCc(c12)sc(c2C(=O)OCC)NC(=O)NS(=O)(=O)c3ccc(C)cc3 | 1000 | 10.1016/j.bmcl.2005.09.047 |
| 439 | c1cc(F)ccc1/C=C2\SC(=S)N(C2=O)NS(=O)(=O)c3ccccc3 | 1000 | 10.1021/jm050859x |
| 440 | O=C(O)c(c1)ccc(c12)n(C3CCCCC3)c(n2)-c4ccc(cc4)OCCCCCc5ccccc5 | 1000 | 10.1016/j.bmcl.2006.01.032 |
| 441 | Cc1cccnc1-c(c2C3CCCCC3)n(C)c(c24)cc(cc4)C(=O)NC(C)(C)C(=O)Nc5ccc(cc5)/C=C/C(=O)O | 1000 | 10.1016/j.bmcl.2011.04.059 |
| 442 | O=C(O)c(c1)ccc(c12)n(CCC(C)C)c(=O)c(c2O)C(=NS3(=O)=O)Nc(c34)cccc4 | 1035 | 10.1021/jm050855s |
| 443 | c1ccncc1C(c2cccnc2)Oc(cc3F)ccc3-c(n4)n(c(c45)ccc(c5)C(=O)O)C6CCCCC6 | 1100 | 10.1016/j.bmcl.2006.01.032 |
| 444 | C1CCCCC1n(c(c23)ccc(c2)C(=O)O)c(n3)-c4ccc(cc4OC)OCc5ccccc5-c6ccccc6 | 1100 | 10.1021/jm060269e |
| 445 | s1cccc1-c(c2O)nn(CCC(C)C)c(=O)c2C(N3)=NS(=O)(=O)c(c34)cc(cc4)NC(=O)C | 1100 | 10.1016/j.bmcl.2008.01.007 |
| 446 | CCC[C@@](CC(=O)O)(OCC1)C(=C12)N=C3[C@@H]2C(C#N)=CC(=C3C)OCCN4CCCC4 | 1100 | 10.1016/j.bmcl.2011.04.052 |
| 447 | c1ccccc1Cn(c(c23)nccc2)c(=O)c(c3O)C(=NS4(=O)=O)Nc(c45)c(Br)ccc5 | 1170 | 10.1016/j.bmcl.2006.04.022 |
| 448 | c1ccnc(c12)n(CCC(C)C)c(=O)c(c2O)C(=NS3(=O)=O)Nc(c34)c(OC)ccc4 | 1170 | 10.1016/j.bmcl.2006.04.022 |
| 449 | c1cc(C)ccc1C(c2ccc(C)cc2)Oc(cc3)ccc3-c(n4)n(c(c45)ccc(c5)C(=O)O)C6CCCCC6 | 1200 | 10.1016/j.bmcl.2006.01.032 |
| 450 | Clc1ccc(Cl)c(c12)[nH]c3c2CC[C@]3(C(=O)O)CC=C | 1200 | 10.1016/j.bmcl.2006.01.105 |
| 451 | c1ccccc1CN([C@H]2C(C)C)C(=O)C(=C2O)C3=NS(=O)(=O)c(c34)c(ccc4)OCCC(=O)N | 1200 | 10.1016/j.bmcl.2008.05.083 |
| 452 | c1cccc(c1C(F)(F)F)Oc(cc2)ccc2/C=C(C(=O)O)\NC(=O)c3ccccc3 | 1300 | 10.1016/j.bmcl.2005.03.066 |
| 453 | CCC[C@@](CC(=O)O)(OCC1)C(=C12)N=C3[C@@H]2C(C#N)=CC(=C3C)C(=O)NC | 1300 | 10.1016/j.bmcl.2011.04.052 |
| 454 | c1cccc(c12)n(CCCCO)c(=O)c(c2O)C(=NS3(=O)=O)Nc(c34)cccc4 | 1301 | 10.1021/jm050855s |
| 455 | O=C(O)CC[C@H](C(=O)O)NC(=O)c1c(F)ccc(c12)c(C(F)(F)F)c(cc2)OC | 1400 | 10.1016/j.bmcl.2004.06.013 |
| 456 | c1cccc(C)c1-c2sc(cc2)/C=C(C(=O)O)\NC(=O)c3ccccc3 | 1400 | 10.1016/j.bmcl.2005.03.066 |
| 457 | N#C[C@@H]1CCCN1S(=O)(=O)c2c(O)c(Cl)cc(Cl)c2 | 1400 | 10.1021/jm060168g |
| 458 | O=c1[nH]cccc1-c(c(c23)cc(S(=O)(=O)C)cc2)c(C(=O)O)n3Cc4ccnc(N)c4 | 1400 | 10.1021/jm201258k |
| 459 | c1cccc(c12)NC(=NS2(=O)=O)c(c3O)c(=O)n(c(c34)cccc4)NCc(sc5Cl)cc5 | 1450 | 10.1016/j.bmcl.2005.01.071 |
| 460 | c1cccc(c12)NC(=NS2(=O)=O)c(c3O)c(=O)n(c(c34)cccc4)N[C@H](C)c5ccccc5 | 1490 | 10.1016/j.bmcl.2005.01.071 |
| 461 | c1cccc(c12)NC(=NS2(=O)=O)c(c(O)c(c34)cccn4)c(=O)n3CCc5ccccc5 | 1490 | 10.1016/j.bmcl.2005.01.071 |
| 462 | Clc1ccc(Cl)c(c12)oc3c2CCO[C@@]3(CCC)CC(=O)O | 1490 | 10.1016/j.bmcl.2005.08.114 |
| 463 | c1cocc1-c(n2)n(C3CCCCC3)c(c24)ccc(c4)C(=O)NCCc5c[nH]c(c56)ccc(c6)O | 1500 | 10.1016/j.bmcl.2003.12.032 |
| 464 | CCC[C@@H](C)Nc1ncnc(c12)nc(-c3ccc(F)cc3)c(n2)-c4ccc(F)cc4 | 1500 | 10.1016/j.bmcl.2004.11.028 |
| 465 | c1ccccc1C(=O)N/C(C(=O)O)=C\c(cc2)sc2-c3ccccc3 | 1500 | 10.1016/j.bmcl.2005.03.066 |
| 466 | c1cc(Cl)ccc1/C=C2\SC(=S)N(C2=O)NS(=O)(=O)c3ccccc3 | 1500 | 10.1021/jm050859x |
| 467 | O=C(O)c(c1)ccc(c12)n(C3CCCC3)c(n2)-c4ccc(cc4)OCc5ccc(cc5)C(C)(C)C | 1500 | 10.1016/j.bmcl.2006.01.032 |
| 468 | N#Cc1c(F)cc(C)c(c12)[nH]c3c2CC[C@@]3(C(=O)O)CCCC | 1500 | 10.1016/j.bmcl.2006.01.105 |
| 469 | CC(C)(C)CCN([C@H]1C(C)(C)C)C(=O)C(=C1O)C2=NS(=O)(=O)c(c23)c(ccc3)OCC | 1500 | 10.1016/j.bmcl.2008.05.083 |
| 470 | O=C(O)Cc(cc1)ccc1NC(=O)[C@H](C)NC(=O)c(cc2)cc(c23)nc(-c4cocc4)n3C5CCCCC5 | 1520 | 10.1016/j.bmcl.2009.10.136 |
| 471 | c1cccc(c12)n(CCC)c(=O)c(c2O)C(=NS3(=O)=O)Nc(c34)cccc4 | 1550 | 10.1021/jm050855s |
| 472 | n1c(N)nc(O)c(c12)ncn2[C@H]3[C@H](OC)[C@H](O)[C@H](O3)CO | 1600 | 10.1021/jm030424e |
| 473 | O=C(O)c(c1)ccc(c12)n(C3CCCC3)c(n2)-c4ccc(cc4)OCc5cccc(Cl)c5 | 1600 | 10.1016/j.bmcl.2006.01.032 |
| 474 | s1cccc1-c(c2O)nn(CCC(C)C)c(=O)c2C(N3)=NS(=O)(=O)c(c34)cc(cc4)OC(C)(C)C(=O)N | 1600 | 10.1016/j.bmcl.2008.01.007 |
| 475 | N#CCOc(ccc1)c(c12)S(=O)(=O)N=C2C(=C3O)C(=O)N([C@H]3C(C)C)Cc4ccccc4 | 1600 | 10.1016/j.bmcl.2008.05.083 |
| 476 | c1ccc(OCC)c(c12)S(=O)(=O)N=C2C(=C3O)C(=O)N([C@H]3C(C)(C)C)Cc4cc(Cl)ccc4 | 1600 | 10.1016/j.bmcl.2008.05.083 |
| 477 | s1cccc1-c2nn(CCC(C)C)c(=O)c(c2O)C(N3)=CS(=O)(=O)c(c34)cc(cc4)N(C)S(=O)(=O)C | 1600 | 10.1016/j.bmcl.2008.07.014 |
| 478 | C1CCCCC1c2c(c(c3)n4C(C)C)ccc3C(=O)NS(=O)(=O)N(C)CCOCCN(C)C(=O)COc(c5c24)cccc5 | 1660 | 10.1016/j.bmcl.2012.03.097 |
| 479 | c1c(F)ccc(F)c1/C=C2\SC(=S)N(C2=O)NS(=O)(=O)c3ccccc3 | 1700 | 10.1021/jm050859x |
| 480 | O=C(O)c(c1)ccc(c12)n(C3CCCC3)c(n2)-c4ccc(cc4)OCc5cc(Cl)cc(Cl)c5 | 1700 | 10.1016/j.bmcl.2006.01.032 |
| 481 | O=C(O)[C@H]1CSCN1S(=O)(=O)c2c(O)c(Cl)cc(Cl)c2 | 1700 | 10.1021/jm060168g |
| 482 | OCCNc1ccc(c2c13)C(=O)N(C(=O)c2ccc3)c4ccccc4 | 1700 | 10.1021/jm900517t |
| 483 | CC/C=C/[C@@](CC(=O)O)(OCC1)C(=C12)N=C3[C@@H]2C(Cl)=CC=C3Cl | 1700 | 10.1016/j.bmcl.2010.03.002 |
| 484 | O=c1[nH]cccc1-c2c(C(=O)O)n(c(c23)ccc4c3scn4)Cc5ccc(F)cc5F | 1700 | 10.1016/j.bmcl.2013.01.024 |
| 485 | COCCOc(c1)ccc(c12)n(CCC(C)C)c(=O)c(c2O)C(=NS3(=O)=O)Nc(c34)cccc4 | 1741 | 10.1021/jm050855s |
| 486 | c1ccn(C)c1-c(n2)n(c(c23)ccc(c3)C(=O)O)C4CCCCC4 | 1800 | 10.1016/j.bmcl.2003.10.023 |
| 487 | c1ccccc1C(=O)N/C(C(=O)O)=C\c2ccc(cc2)Oc3c(SC)cccc3 | 1800 | 10.1016/j.bmcl.2005.03.066 |
| 488 | C1CCCCCC1n(c(c23)ccc(c2)C(=O)O)c(n3)-c4ccc(cc4)OCc5ccccc5 | 1800 | 10.1016/j.bmcl.2006.01.032 |
| 489 | c1cc(Cl)ccc1Cn2cc(c(=O)c(c23)cc(OC)cc3)-c4oc(cn4)Cc5ccccc5 | 1800 | 10.1016/j.bmcl.2011.11.013 |
| 490 | c1cccnc1-c(n2)n(C3CCCCC3)c(c24)ccc(c4)C(=O)N[C@H](C(=O)O)Cc5c[nH]c(c56)cccc6 | 1900 | 10.1016/j.bmcl.2003.12.032 |
| 491 | O[C@]1(C)[C@H](O)[C@@H](CO)O[C@H]1n(cn2)c(c23)ncnc3N | 1900 | 10.1021/jm030424e |
| 492 | c1ccc(O)cc1NC(=O)/C(NC(=O)c2ccccc2)=C/c3ccc(cc3)Oc4c(Br)cccc4 | 1900 | 10.1016/j.bmcl.2005.03.106 |
| 493 | c1cc(F)c(F)c(F)c1/C=C2\SC(=S)N(C2=O)NS(=O)(=O)c3ccccc3 | 1900 | 10.1021/jm050859x |
| 494 | C1CC1C[C@](CC(=O)O)(OCC2)C(=C23)N=C4[C@@H]3C(Cl)=CC=C4Cl | 1900 | 10.1016/j.bmcl.2010.03.002 |
| 495 | O1CC(=O)NCCCCN(C)S(=O)(=O)NC(=O)c(cc2)cc(n3C)c2c(c3c(c14)cccc4)C5CCCCC5 | 1900 | 10.1016/j.bmcl.2012.03.097 |
| 496 | c1cccnc1-c(n2)n(C3CCCCC3)c(c24)ccc(c4)C(=O)N[C@H](C(=O)O)c5cc(OC)c(cc5)OC | 2000 | 10.1016/j.bmcl.2003.12.032 |
| 497 | OCC(=O)NC(\C(=O)O)=C/c1ccc(cc1)Oc2c(I)cccc2 | 2000 | 10.1016/j.bmcl.2005.03.106 |
| 498 | C[C@@H]1CCCc(c12)sc(c2C(=O)OCC)NC(=O)NS(=O)(=O)c3ccc(C)cc3 | 2000 | 10.1016/j.bmcl.2005.09.047 |
| 499 | c1cc([N+](=O)[O-])ccc1/C=C2\SC(=S)N(C2=O)NS(=O)(=O)c3ccccc3 | 2000 | 10.1021/jm050859x |
| 500 | CS(=O)(=O)NN(C1=O)C(=S)S/C1=C\c2cc(Cl)c(Cl)cc2 | 2000 | 10.1021/jm050859x |
| 501 | C1CCC[C@@H](C(=O)O)N1S(=O)(=O)c2c(O)c(Cl)cc(Cl)c2 | 2000 | 10.1021/jm060168g |
| 502 | c1cccc(c12)n(C(=O)OCC)c(=O)c(c2O)C(=NS3(=O)=O)Nc(c34)cccc4 | 2036 | 10.1021/jm050855s |
| 503 | C1CCC1Nc2ncnc(c23)nc(-c4ccc(F)cc4)c(n3)-c5ccc(F)cc5 | 2100 | 10.1016/j.bmcl.2004.11.028 |
| 504 | O=C(O)[C@@H]1CCCN1S(=O)(=O)c2c(O)c(Cl)cc(F)c2 | 2100 | 10.1021/jm060168g |
| 505 | s1cccc1-c(c2O)nn(CCC(C)C)c(=O)c2C(N3)=NS(=O)(=O)c(c34)cc(cc4)-c5conc5 | 2100 | 10.1016/j.bmcl.2008.01.007 |
| 506 | c1cocc1-c(n2)n(C3CCCCC3)c(c24)ccc(c4)C(=O)N[C@H](C(=O)O)Cc5c[nH]c(c56)ccc(c6)C | 2200 | 10.1016/j.bmcl.2003.12.032 |
| 507 | COc(c1)c(OC)cc(c12)c(=O)c(C(=O)c3ccc(C)cc3)cn2CCCc4ccccc4 | 2200 | 10.1016/j.bmcl.2010.11.068 |
| 508 | c1cccc(c12)n(CC)c(=O)c(c2O)C(=NS3(=O)=O)Nc(c34)cccc4 | 2220 | 10.1021/jm050855s |
| 509 | OC(=O)c(n1)c(O)c(O)nc1-c2ccccc2 | 2300 | 10.1021/jm051064t |
| 510 | O=C(O)C(=O)/C=C(O)/c1cc(ccc1)OCCCC#N | 2500 | 10.1021/jm0342109 |
| 511 | c1cccc(c12)n(CC[S@](=O)C)c(=O)c(c2O)C(=NS3(=O)=O)Nc(c34)cccc4 | 2537 | 10.1021/jm050855s |
| 512 | O[C@@H]1[C@@H](CO)O[C@H]([C@@]1(O)C)n2cnc(c23)c(N)nc(n3)N | 2600 | 10.1021/jm030424e |
| 513 | c1cccnc1Cn(c(c23)cccc2)c(=O)c(c3O)C(=NS4(=O)=O)Nc(c45)cccc5 | 2694 | 10.1021/jm050855s |
| 514 | O=c1[nH]cccc1-c2c(S(=O)(=O)N)n(c(c23)ccc(Cl)c3)Cc4ccc(F)cc4F | 2700 | 10.1016/j.bmcl.2011.10.041 |
| 515 | C1CCCCC1N(c2sc(cn2)C(=O)O)C(=O)c3ccc(cc3)Oc4cc(Cl)ccc4 | 2800 | 10.1016/j.bmcl.2004.10.024 |
| 516 | C1CCCc(c12)sc(c2C(=O)OCC)NC(=O)NS(=O)(=O)c3ccc([N+](=O)[O-])cc3 | 2800 | 10.1016/j.bmcl.2005.09.047 |
| 517 | Fc1ccccc1Cn(c(c23)ccc(Cl)c3)c(C(=O)NS(=O)(=O)CCCCl)c2-c4ccc[nH]c4=O | 2800 | 10.1016/j.bmcl.2011.10.041 |
| 518 | N#Cc1ccc(C)c(c12)sc3c2CCO[C@@]3(CCC)CC(=O)O | 2850 | 10.1016/j.bmcl.2005.08.114 |
| 519 | C#CCOC(=O)N(CC(=O)O)C(=O)c1c(Br)ccc(c12)c(C(F)(F)F)c(cc2)OC | 2900 | 10.1016/j.bmcl.2004.06.013 |
| 520 | c1cc(F)ccc1-c(n2)c(-c3ccc(F)cc3)nc(c24)ncnc4NCc5occc5 | 2900 | 10.1016/j.bmcl.2004.11.028 |
| 521 | s1cccc1[C@@H]2N(CCC(C)(C)C)C(=O)C(=C2O)C3=NS(=O)(=O)c(c34)c(ccc4)OCC | 2900 | 10.1016/j.bmcl.2008.05.083 |
| 522 | o1cc(C)c(c12)ccc3c2c(-c4ccc[nH]c4=O)c(C(=O)O)n3Cc5cc(F)ccc5F | 2900 | 10.1016/j.bmcl.2013.01.024 |
| 523 | c1cccnc1-c(n2)n(C3CCCCC3)c(c24)ccc(c4)C(=O)N[C@H](C)c5cc(OC)c(cc5)OC | 3000 | 10.1016/j.bmcl.2003.12.032 |
| 524 | c1cc(Br)ccc1-c(n2)c(-c3ccc(Br)cc3)nc(c24)ncnc4O | 3000 | 10.1016/j.bmcl.2004.11.028 |
| 525 | c1cccc(c12)ccc(c2)/C=C3\SC(=S)N(C3=O)NS(=O)(=O)c4ccccc4 | 3000 | 10.1021/jm050859x |
| 526 | CCOC(=O)N(CC(=O)O)C(=O)c1c(Br)ccc(c12)c(C(F)(F)F)c(cc2)OC | 3100 | 10.1016/j.bmcl.2004.06.013 |
| 527 | c1ccccc1-c(n2)n(c(c23)ccc(c3)C(=O)O)C4CCCCC4 | 3100 | 10.1016/j.bmcl.2006.07.074 |
| 528 | c1ccsc1[C@@H]([C@H](C2)C(=O)O)N([C@@]2(C(=O)O)CC(C)C)C(=O)c(cc3)ccc3[N+]([O-])=O | 3200 | 10.1016/j.bmcl.2005.01.076 |
| 529 | O=C(O)c(c1)ccc(c12)n(C3CCCC3)c(n2)-c4ccc(cc4)OCc5ccccc5 | 3200 | 10.1016/j.bmcl.2006.01.032 |
| 530 | Clc1ccc(Cl)c(c12)[nH]c3c2CCC[C@@]3(C(=O)O)CCC | 3200 | 10.1016/j.bmcl.2006.01.105 |
| 531 | c1ccccc1CN([C@H]2C(C)C)C(=O)C(=C2O)C3=NS(=O)(=O)c(c34)c(ccc4)OCC(=O)N5CCOCC5 | 3200 | 10.1016/j.bmcl.2008.05.083 |
| 532 | CC[C@H](C)[C@](CC(=O)O)(OCC1)C(=C12)N=C3[C@@H]2C(C#N)=CC=C3C | 3200 | 10.1016/j.bmcl.2010.03.002 |
| 533 | C1CCCC1[C@](CC(=O)O)(OCC2)C(=C23)N=C4[C@@H]3C(Cl)=CC=C4Cl | 3300 | 10.1016/j.bmcl.2010.03.002 |
| 534 | C1C[C@@H](C(C)(C)C)CC[C@H]1N(c2sc(cn2)C(=O)O)C(=O)c3ccc(cc3)Oc4ccccc4 | 3400 | 10.1016/j.bmcl.2004.10.024 |
| 535 | C1CCCCC1N(c2sc(cn2)C(=O)O)C(=O)c3ccc(cc3)Oc(cc4OC)ccc4OC | 3500 | 10.1016/j.bmcl.2004.10.024 |
| 536 | c1cccc(c12)NC(=NS2(=O)=O)C3=C(O)[C@@H](N(C3=O)CCC(C)C)Cc4ccccc4 | 3567 | 10.1016/j.bmcl.2006.01.034 |
| 537 | c1ccoc1-c(n2)n(c(c23)ccc(c3)C(=O)O)C4CCCCC4 | 3700 | 10.1016/j.bmcl.2003.10.023 |
| 538 | O=C(O)C(=O)CC(=O)c1c(OCCC)cccc1 | 3900 | 10.1021/jm0342109 |
| 539 | c1ccccc1C(=O)N/C(C(=O)O)=C\c(cc2)oc2-c3cc(F)ccc3 | 3900 | 10.1016/j.bmcl.2005.03.066 |
| 540 | c1ccccc1Cn(c(c23)nccc2)c(=O)c(c3O)C(=NS4(=O)=O)Nc(c45)cc(cc5)OC | 3900 | 10.1016/j.bmcl.2006.04.022 |
| 541 | c1ccccc1COc(c2)ccc(c23)n(CCC(C)C)c(=O)c(c3O)C(=NS4(=O)=O)Nc(c45)cccc5 | 3950 | 10.1021/jm050855s |
| 542 | c1cocc1-c(n2)n(C3CCCCC3)c(c24)ccc(c4)C(=O)N[C@H](C(=O)O)Cc5ccccc5 | 4000 | 10.1016/j.bmcl.2003.12.032 |
| 543 | CCOC(=O)c1c(O)c(=O)cc(o1)C(=O)OCC | 4000 | 10.1016/j.bmcl.2004.03.087 |
| 544 | C1CCCCC1N(c2sc(cn2)C(=O)O)C(=O)c3ccc(cc3)OCc4c(F)cccc4 | 4000 | 10.1016/j.bmcl.2004.10.024 |
| 545 | c1cccc(OC(F)(F)F)c1-c2oc(cc2)/C=C(C(=O)O)\NC(=O)c3ccccc3 | 4000 | 10.1016/j.bmcl.2005.03.066 |
| 546 | O=C(O)C(=O)/C=C(O)/c1cscc1 | 4000 | 10.1021/jm0504454 |
| 547 | C1CCCc(c12)sc(c2C(=O)OCC)NC(=O)c3c(C(=O)O)nccn3 | 4000 | 10.1016/j.bmcl.2005.09.047 |
| 548 | c1ccc(OCC)c(c12)S(=O)(=O)N=C2C(=C3O)C(=O)N(CCC(C)(C)C)[C@H]3C4CCCCC4 | 4000 | 10.1016/j.bmcl.2008.05.083 |
| 549 | O=C(O)c(c1)ccc(c12)n(C3CCCC3)c(n2)-c4ccc(cc4)OCc5ccncc5 | 4200 | 10.1016/j.bmcl.2006.01.032 |
| 550 | c1ccccc1C(=O)Nc(c2)ccc(c23)n(CCC(C)C)c(=O)c(c3O)C(=NS4(=O)=O)Nc(c45)cccc5 | 4235 | 10.1021/jm050855s |
| 551 | CC(C)c1ccc(cc1)CC[C@]2(OC(=O)CC(=O)C2)C3CCCC3 | 4300 | 10.1016/j.bmcl.2006.06.065 |
| 552 | c1cc(Cl)ccc1Cn2cc(c(=O)c(c23)cc(OC)c(c3)OC)C4=N[C@H](C)[C@H](O4)c5ccccc5 | 4300 | 10.1016/j.bmcl.2011.11.013 |
| 553 | c1cscc1C(=O)CC(=O)C(=O)O | 4500 | 10.1021/jm0342109 |
| 554 | c1ccccc1C(=O)N/C(C(=O)O)=C\c(cc2)oc2-c3ccc(Cl)cc3 | 4600 | 10.1016/j.bmcl.2005.03.066 |
| 555 | O=C(O)C(=O)/C=C(O)/c1cn(cc1)Cc2cc(C#N)ccc2 | 4600 | 10.1021/jm0504454 |
| 556 | O[C@@H](C)C(=O)N/C(C(=O)O)=C\c1ccc(cc1)Oc2c(I)cccc2 | 4800 | 10.1016/j.bmcl.2005.03.106 |
| 557 | C1CCCN1S(=O)(=O)c2c(O)c(Cl)cc(Cl)c2 | 4800 | 10.1021/jm060168g |
| 558 | CCCOC(=O)N(CC(=O)O)C(=O)c1c(Br)ccc(c12)c(C(F)(F)F)c(cc2)OC | 4900 | 10.1016/j.bmcl.2004.06.013 |
| 559 | c1cc(C)ccc1C(=O)CC(=O)C(=O)O | 5000 | 10.1021/jm0342109 |
| 560 | O=C(O)CN(C(=O)OC)C(=O)c1c(Cl)ccc(c12)c(C(F)(F)F)c(cc2)OC | 5000 | 10.1016/j.bmcl.2004.06.013 |
| 561 | Clc1cccc(c1)CC[C@]2(OC(=O)CC(=O)C2)C3CCCC3 | 5000 | 10.1016/j.bmcl.2006.06.065 |
| 562 | CCc(co1)c(c12)ccc3c2c(-c4ccc[nH]c4=O)c(C(=O)O)n3Cc5cc(F)ccc5F | 5000 | 10.1016/j.bmcl.2013.01.024 |
| 563 | O=C(O)CN(C)C(=O)c1c(Cl)ccc(c12)c(C(F)(F)F)c(cc2)OC | 5100 | 10.1016/j.bmcl.2004.06.013 |
| 564 | C1CCCCC1N(c2sc(cn2)C(=O)O)C(=O)c3ccc(cc3)OCc(c4C(F)(F)F)cccc4 | 5100 | 10.1016/j.bmcl.2004.10.024 |
| 565 | CC(C)CCN([C@H]1C(C)(C)C)C(=O)C(=C1O)C2=NS(=O)(=O)c(c23)c(ccc3)OCC | 5100 | 10.1016/j.bmcl.2008.05.083 |
| 566 | C1CCCCC1N(c2sc(cn2)C(=O)O)C(=O)c3ccc(cc3)Oc(ccc4)cc4OC(F)(F)F | 5200 | 10.1016/j.bmcl.2004.10.024 |
| 567 | c1cccc(Cl)c1-c2sc(nc2)/C=C(C(=O)O)\NC(=O)c3ccccc3 | 5200 | 10.1016/j.bmcl.2005.03.066 |
| 568 | s1cccc1-c(c2O)nn(CCC(C)C)c(=O)c2C(N3)=NS(=O)(=O)c(c34)cc(cc4)C5=CCCC5=O | 5300 | 10.1016/j.bmcl.2008.01.007 |
| 569 | C1C[C@H](C)CC[C@@H]1N(c2nc(cs2)C(=O)O)C(=O)c3ccc(cc3)Oc4ccccc4 | 5400 | 10.1016/j.bmcl.2004.10.024 |
| 570 | c1cccc(C(F)(F)F)c1-c2oc(cc2)/C=C(C(=O)O)\NC(=O)c3ccccc3 | 5600 | 10.1016/j.bmcl.2005.03.066 |
| 571 | FC(F)(F)c1ccc(Cl)c(c12)[nH]c3c2CCC[C@@]3(C(=O)O)CCC | 5800 | 10.1016/j.bmcl.2006.01.105 |
| 572 | O=C(O)[C@@H]1CCCN1S(=O)(=O)c2c(N)cc(C)c(Cl)c2 | 5950 | 10.1021/jm060168g |
| 573 | n1cc(C(=O)O)sc1N([C@@H](CC2(C)C)C[C@H](C2)C)C(=O)c3ccc(cc3)Oc4ccccc4 | 6000 | 10.1016/j.bmcl.2004.10.024 |
| 574 | c1cc(Cl)c(Cl)cc1/C=C2\SC(=S)N([C@@H]2O)NS(=O)(=O)c3ccc(cc3)OC | 6000 | 10.1021/jm050859x |
| 575 | N#Cc1c(F)cc(C)c(c12)[nH]c3c2CC[C@]3(C(=O)O)CC=C | 6300 | 10.1016/j.bmcl.2006.01.105 |
| 576 | s1cccc1-c(c2O)nn(CCC(C)C)c(=O)c2C(N3)=NS(=O)(=O)c(c34)cc(cc4)O[C@H](C)C(=O)OCC | 6300 | 10.1016/j.bmcl.2008.01.007 |
| 577 | O=C(O)C[C@](CC)(OCC1)C(=C12)N=C3[C@@H]2C(Cl)=CC=C3Cl | 6300 | 10.1016/j.bmcl.2010.03.002 |
| 578 | c1cscc1-c(n2)n(c(c23)ccc(c3)C(=O)O)C4CCCCC4 | 6330 | 10.1016/j.bmcl.2003.10.023 |
| 579 | CC(C)(C)COC(=O)N(CC(=O)O)C(=O)c1c(Br)ccc(c12)c(C(F)(F)F)c(cc2)OC | 6400 | 10.1016/j.bmcl.2004.06.013 |
| 580 | COc1ccc(cc1C(C)(C)C)N2CCC(=O)NC2=O | 6400 | 10.1016/j.bmcl.2012.04.017 |
| 581 | COc1cc(NC(=O)C)c(cc1C(C)(C)C)N2CCC(=O)NC2=O | 6500 | 10.1016/j.bmcl.2012.04.017 |
| 582 | CC(C)CC(=O)Nc(c1)ccc(c12)n(CCC(C)C)c(=O)c(c2O)C(=NS3(=O)=O)Nc(c34)cccc4 | 6503 | 10.1021/jm050855s |
| 583 | O=C(O)C(=O)/C=C(O)/c1cc(ccc1)OCc(cc2C#N)ccc2 | 6700 | 10.1021/jm0342109 |
| 584 | COc1cc(N(C)C)c(cc1C(C)(C)C)N2CCC(=O)NC2=O | 6700 | 10.1016/j.bmcl.2012.04.017 |
| 585 | c1cccnc1-c(n2)n(C3CCCCC3)c(c24)ccc(c4)C(=O)NCc(c5)ccc(OC)c5OC | 7000 | 10.1016/j.bmcl.2003.12.032 |
| 586 | C1CCCCC1N(c2sc(cn2)C(=O)O)C(=O)c3ccc(cc3)OCc4sccc4 | 7000 | 10.1016/j.bmcl.2004.10.024 |
| 587 | C1CCCCC1N(c2sc(cn2)C(=O)O)C(=O)c3ccc(cc3)Oc4ccccc4 | 7000 | 10.1016/j.bmcl.2004.10.024 |
| 588 | C1CCCC1CCC(=O)NC(\C(=O)O)=C/c2ccc(cc2)Oc3c(I)cccc3 | 7000 | 10.1016/j.bmcl.2005.03.106 |
| 589 | O=C1N(N)C(=S)S/C1=C\c2ccc(F)cc2 | 7000 | 10.1021/jm050859x |
| 590 | O=c1[nH]cccc1-c2c(C(=O)O)n(Cc(ccn3)cc3N)c(c24)ccc(c4)-c5cccc(=O)[nH]5 | 7000 | 10.1021/jm201258k |
| 591 | O=C(O)C(=O)/C=C(O)/c1c(scc1)Sc2cc(Cl)ccc2 | 7500 | 10.1021/jm0504454 |
| 592 | C1CCC[C@@H](C)[C@@H]1N(c2nc(cs2)C(=O)O)C(=O)c3ccc(cc3)Oc4ccccc4 | 7600 | 10.1016/j.bmcl.2004.10.024 |
| 593 | O=C(O)[C@H](C1)C[C@@H](C(=O)O)N1C(=O)c2ccc(C(F)(F)F)cc2 | 7600 | 10.1016/j.bmcl.2005.01.076 |
| 594 | O=C(O)C(=O)CC(=O)c1cc(ccc1)OCc2ccccc2 | 8000 | 10.1021/jm0342109 |
| 595 | N#Cc1ccc(F)c(c12)[nH]c3c2CC[C@]3(C(=O)O)CCC | 8100 | 10.1016/j.bmcl.2006.01.105 |
| 596 | O=C(O)C(=O)/C=C(O)/c1c(cccc1)Cc2ccc(F)cc2 | 8300 | 10.1021/jm0504454 |
| 597 | n1cnc(N)c(c12)c(-c(nn3)cn3C)cn2[C@@H]([C@@]4(C)O)O[C@H](CO)[C@@H]4O | 8400 | 10.1016/j.bmcl.2012.05.067 |
| 598 | O=P(O)(O)O[P@](=O)(O)O[P@@](=O)(O)OC[C@@H]([C@@H](O)[C@@]1(C)O)O[C@@H]1n(c(c23)ncnc3N)cc2C4=CN=NC4 | 8400 | 10.1016/j.bmcl.2012.05.067 |
| 599 | Cc1noc(n1)-c(c(c23)c(N)ncn3)cn2[C@@H]([C@@]4(C)O)O[C@@H]([C@@H]4O)CO[P@@](=O)(O)O[P@](=O)(O)OP(=O)(O)O | 8400 | 10.1016/j.bmcl.2012.05.067 |
| 600 | C1CCCCC1N(c2sc(cn2)C(=O)O)C(=O)c3ccc(cc3)Oc4c(OC)cccc4 | 8500 | 10.1016/j.bmcl.2004.10.024 |
| 601 | C1CCCCC1N(c2sc(cn2)C(=O)O)C(=O)c3ccc(cc3)OCc4c(OC)cccc4 | 8500 | 10.1016/j.bmcl.2004.10.024 |
| 602 | c1ccccc1C(=O)N/C(C(=O)O)=C\c2ccc(cc2)Oc3c(CC)cccc3 | 9400 | 10.1016/j.bmcl.2005.03.066 |
| 603 | O=C(O)c(n1)c(O)c(O)nc1-c2scc(c2)[N+](=O)[O-] | 9400 | 10.1021/jm051064t |
| 604 | C1CCCC1C[C@](CC(=O)O)(OCC2)C(=C23)N=C4[C@@H]3C(Cl)=CC=C4Cl | 9400 | 10.1016/j.bmcl.2010.03.002 |
| 605 | O=C(C1)NCCCCCCNC(=O)c(c2)ccc(c2n13)c(c3-c4cocc4)C5CCCCC5 | 9500 | 10.1016/j.bmcl.2012.03.097 |
| 606 | O=C(O)C(=O)/C=C(O)/c1c(ccs1)Cc2cc(F)ccc2 | 9900 | 10.1021/jm0504454 |
| 607 | c1cccc(c12)n(CCC(C)C)c(=O)n(c2=O)C(=NS3(=O)=O)Nc(c34)cccc4 | 9940 | 10.1021/jm050855s |
| 608 | [nH]1ccnc1-c(n2)n(c(c23)ccc(c3)C(=O)O)C4CCCCC4 | 10000 | 10.1016/j.bmcl.2003.10.023 |
| 609 | c1cc(F)ccc1-c(n2)c(-c3ccc(F)cc3)nc(c24)ncnc4NC[C@@H](CC)CCCC | 10000 | 10.1016/j.bmcl.2004.11.028 |
| 610 | c1cc(C)ccc1/C=C2\SC(=S)N(C2=O)NS(=O)(=O)c3ccccc3 | 10000 | 10.1021/jm050859x |
| 611 | N#Cc1ccc(F)c(c12)[nH]c3c2CCC[C@@]3(C(=O)O)CCC | 10000 | 10.1016/j.bmcl.2006.01.105 |
| 612 | c1cc(Cl)ccc1Cn2cc(c(=O)c(c23)cc(OC)c(c3)OC)-c4onc(n4)-c5ccc(Cl)cc5 | 10000 | 10.1016/j.bmcl.2011.11.013 |
| 613 | c1cc(Cl)ccc1Cn2cc(c(=O)c(c23)cc(OC)c(c3)OC)-c4nnn(n4)Cc5ccccc5 | 10000 | 10.1016/j.bmcl.2011.11.013 |
| 614 | c1cc(Cl)ccc1Cn2cc(c(=O)c(c23)cc(OC)c(c3)OC)-c4noc(n4)CCc5ccccc5 | 10000 | 10.1016/j.bmcl.2011.11.013 |
| 615 | c1ccccc1C(=O)N/C(C(=O)O)=C\c(ccc2)nc2-c3c(Cl)cccc3 | 11000 | 10.1016/j.bmcl.2005.03.066 |
| 616 | s1cccc1-c(c2O)nn(CCC(C)C)c(=O)c2C(N3)=NS(=O)(=O)c(c34)cc(cc4)[C@H]5CCCC5=O | 11000 | 10.1016/j.bmcl.2008.01.007 |
| 617 | c1cc(OC)ccc1-c(nc(c23)cc(cc3)C(=O)O)n2C4CCCCC4 | 11200 | 10.1016/j.bmcl.2006.05.012 |
| 618 | O=C(O)CN(C(=O)OC)C(=O)c1c(OC)ccc(c12)c(C(F)(F)F)c(cc2)OC | 11300 | 10.1016/j.bmcl.2004.06.013 |
| 619 | c1ccsc1[C@@H]([C@H](C2)C(=O)O)N([C@@]2(C(=O)O)CC(C)C)C(=O)c3ccccc3 | 11400 | 10.1016/j.bmcl.2005.01.076 |
| 620 | c1cccnc1-c(n2)n(C3CCCC3)c(c24)ccc(c4)C(=O)O | 12000 | 10.1016/j.bmcl.2003.10.023 |
| 621 | c1ccccc1C(=O)N/C(C(=O)O)=C\c(cc2)oc2-c3ccc(F)cc3 | 12000 | 10.1016/j.bmcl.2005.03.066 |
| 622 | c1cc(O)ccc1NC(=O)/C(NC(=O)c2ccccc2)=C/c3ccc(cc3)Oc4c(Br)cccc4 | 12000 | 10.1016/j.bmcl.2005.03.106 |
| 623 | O=C(O)C(=O)/C=C(O)/c1cc(C)ccc1 | 12000 | 10.1021/jm0504454 |
| 624 | O=P(O)(O)O[P@](=O)(O)O[P@@](=O)(O)OC[C@@H]([C@@H](O)[C@@]1(C)O)O[C@@H]1n(c(c23)ncnc3N)cc2-c4ncccn4 | 12000 | 10.1016/j.bmcl.2012.05.067 |
| 625 | O=C(O)c(c1)ccc(c12)n([C@H]3[C@@H](C)CCCC3)c(n2)-c4ncccc4 | 13000 | 10.1016/j.bmcl.2003.10.023 |
| 626 | C1CSCCC1N(c2sc(cn2)C(=O)O)C(=O)c3ccc(cc3)Oc4ccccc4 | 13000 | 10.1016/j.bmcl.2004.10.024 |
| 627 | c1cc(F)ccc1-c(n2)c(-c3ccc(F)cc3)nc(c24)ncnc4NCCc5ccccc5 | 13000 | 10.1016/j.bmcl.2004.11.028 |
| 628 | O=C(O)C(=O)/C=C(O)/c(ccc1)n1Cc2ccc(O)cc2 | 13000 | 10.1021/jm0504454 |
| 629 | CC(C)(C)CCN1C(=O)C(=C(O)[C@]1(C)C(C)C)C2=CS(=O)(=O)c(c23)c(ccc3)CN(C)S(=O)(=O)C | 13000 | 10.1016/j.bmcl.2008.05.083 |
| 630 | c1cccc(c12)NC(=NS2(=O)=O)c(c(=O)n3C(CC)CC)c(O)c(c34)cccn4 | 13200 | 10.1016/j.bmcl.2005.01.071 |
| 631 | c1ccccc1-c(nc(c23)sc(c3)C(=O)O)n2C4CCCCC4 | 13800 | 10.1016/j.bmcl.2006.05.012 |
| 632 | c1c(N)ccc(c1C(F)(F)F)Oc(cc2)ccc2\C=C(\C(=O)O)NC(=O)CCC3CCCC3 | 14000 | 10.1016/j.bmcl.2005.03.106 |
| 633 | c1c(C)oc(C)c1\C(O)=C\C(=O)C(=O)O | 14000 | 10.1021/jm0504454 |
| 634 | COc1ccc(cc1OC)CCNC(=O)c(cc2)cc(c23)nc(n3C4CCCCC4)-c5ccc(cc5)OCC(=O)NCCCN(C)C | 14000 | 10.1016/j.bmcl.2009.10.136 |
| 635 | N#Cc1ccc(F)c(c12)[nH]c3c2CC[C@@]3(C(=O)O)CC=C | 14500 | 10.1016/j.bmcl.2006.01.105 |
| 636 | c1ccccc1-c(n2)c(-c3ccccc3)nc(c24)ncnc4O | 15000 | 10.1016/j.bmcl.2004.11.028 |
| 637 | c1ccccc1C(=O)N/C(C(=O)O)=C\c2ccc(cc2)Oc3c(OC)cccc3 | 15000 | 10.1016/j.bmcl.2005.03.066 |
| 638 | O1COc(c12)cc(cc2)/C=C3\SC(=S)N(C3=O)NS(=O)(=O)c4ccccc4 | 15000 | 10.1021/jm050859x |
| 639 | N#Cc1ccc(C)c(c12)[nH]c3c2CCC[C@@]3(C(=O)O)CCC | 15000 | 10.1016/j.bmcl.2006.01.105 |
| 640 | O=C(O)C(=O)CC(=O)c1cc(ccc1)NS(=O)(=O)c2ccccc2 | 16000 | 10.1021/jm0342109 |
| 641 | c1cc(OC)ccc1Cn2c(ccc2)\C=C\C(O)=C\C(=O)C(=O)O | 16000 | 10.1021/jm0504454 |
| 642 | Clc1ccc(cc1)/C=C2\SC(=S)N(C2=O)N | 16000 | 10.1021/jm050859x |
| 643 | O=C(O)c(n1)c(O)c(O)nc1-c2sc([N+](=O)[O-])cc2 | 16000 | 10.1021/jm051064t |
| 644 | C1COCCN1c(c(F)c2)cc(c23)n(Cc4ccc(Cl)cc4)cc(c3=O)C(=O)NCc5ccc(Cl)cc5 | 16000 | 10.1016/j.bmcl.2010.11.068 |
| 645 | CCC[C@@](CC(=O)O)(OCC1)C(=C12)N=C3[C@@H]2C(C#N)=CC(=C3C)C(=O)N(C)C | 16000 | 10.1016/j.bmcl.2011.04.052 |
| 646 | Clc1ccc(Cl)c(c12)oc3c2CCO[C@]3(CC)CC(=O)O | 16300 | 10.1016/j.bmcl.2005.08.114 |
| 647 | c1cnccc1-c(n2)n(c(c23)ccc(c3)C(=O)O)C4CCCCC4 | 17000 | 10.1016/j.bmcl.2003.10.023 |
| 648 | c1ccccc1C(=O)N/C(C(=O)O)=C\c2ccc(cc2)Oc3ccccc3 | 17000 | 10.1016/j.bmcl.2005.03.066 |
| 649 | O=C(O)C(=O)/C=C(O)/c1cc(ccc1)NCc2ccccc2 | 17000 | 10.1021/jm0504454 |
| 650 | Clc1ccc(Cl)c(c12)sc3c2CCO[C@]3(C)CC(=O)O | 17600 | 10.1016/j.bmcl.2005.08.114 |
| 651 | O=C(O)C(=O)/C=C(O)/c(cc(I)c1)n1Cc2ccc(F)cc2 | 18000 | 10.1021/jm0504454 |
| 652 | c1cccc(C)c1Cn2c(ccc2)\C=C\C(O)=C\C(=O)C(=O)O | 18000 | 10.1021/jm0504454 |
| 653 | O=C(O)C(=O)/C=C(O)/c1cc(ccc1)NC(=O)C(=O)O | 19000 | 10.1021/jm0504454 |
| 654 | Nc(c1)nccc1Cn(c(c23)ccc(Cl)c3)c(C(=O)OCC)c2-c4ccc[nH]c4=O | 20000 | 10.1016/j.bmcl.2011.10.041 |
| 655 | Nc1cc(ccn1)Cn(c(c23)ccc(c3)O)c(C(=O)O)c2-c4ccc[nH]c4=O | 20000 | 10.1021/jm201258k |
| 656 | S=C1NC(=O)/C(S1)=C/c(cc2)ccc2[N+]([O-])=O | 22000 | 10.1021/jm050859x |
| 657 | Cc1cccc(c1)CC[C@]2(OC(=O)CC(=O)C2)C3CCCC3 | 23000 | 10.1016/j.bmcl.2006.06.065 |
| 658 | O=C(O)C(=O)/C=C(O)/c(ccc1)n1Cc2c(Cl)cccc2 | 24000 | 10.1021/jm0504454 |
| 659 | O=C(O)C(=O)CC(=O)c1c(cccc1)OCCCCC | 25000 | 10.1021/jm0342109 |
| 660 | [O-][N+](=O)c1cc(ccc1)-c2oc(cc2)/C=C(C(=O)O)\NC(=O)c3ccccc3 | 25000 | 10.1016/j.bmcl.2005.03.066 |
| 661 | c1ccc(C)cc1/C=C2\SC(=S)N(C2=O)NS(=O)(=O)c3ccccc3 | 25000 | 10.1021/jm050859x |
| 662 | CN1CCN(CC1)c(c2)c(F)cc(c23)c(=O)c(cn3Cc4ccccc4)NC(=O)NCc5ccccc5 | 26000 | 10.1016/j.bmcl.2010.11.068 |
| 663 | c1cccnc1-c(n2)n(c(c23)ccc(c3)C(=O)N)C4CCCCC4 | 27000 | 10.1016/j.bmcl.2003.10.023 |
| 664 | c1ccccc1C(=O)N/C(C(=O)O)=C\c(cc2)oc2-c3ccc(SC)cc3 | 27000 | 10.1016/j.bmcl.2005.03.066 |
| 665 | c1cc(Cl)c(Cl)cc1C[C@@H]2SC(=S)N([C@H]2O)NS(=O)(=O)c3ccccc3 | 27000 | 10.1021/jm050859x |
| 666 | c1ccncc1-c(n2)n(c(c23)ccc(c3)C(=O)O)C4CCCCC4 | 28000 | 10.1016/j.bmcl.2003.10.023 |
| 667 | OC[C@H]1O[C@H]([C@@H]([C@@H]1F)O)n(cn2)c(c23)ncnc3N | 28000 | 10.1021/jm030424e |
| 668 | c1ccccc1C(=O)N/C(C(=O)O)=C\c2ccc(cc2)Oc3c(C(C)C)cccc3 | 28000 | 10.1016/j.bmcl.2005.03.066 |
| 669 | O=C(O)C(=O)/C=C(O)/c1cc(ccc1)-c2ccccc2 | 28000 | 10.1021/jm0504454 |
| 670 | O=C(O)C(=O)/C=C(O)/c(ccc1)n1Cc2ccccc2 | 29000 | 10.1021/jm0504454 |
| 671 | c1ccccc1-c(n2)c(-c(cc3)ccc3[N+]([O-])=O)nc(c24)ncnc4O | 30000 | 10.1016/j.bmcl.2004.11.028 |
| 672 | c1ccccc1/C=C2\SC(=S)N(C2=O)NS(=O)(=O)c3ccccc3 | 30000 | 10.1021/jm050859x |
| 673 | Fc1cc(F)cc(c12)[nH]c3c2CCC[C@]3(CCC)CC(=O)O | 30000 | 10.1016/j.bmcl.2006.01.105 |
| 674 | O=C(O)C(=O)/C=C(O)/c1cc(ccc1)Cc2cc(Br)cc(Br)c2 | 32000 | 10.1021/jm0504454 |
| 675 | COc(cc1)cc(c12)S(=O)(=O)NC(=N2)c3c(O)c(nn(c3=O)CCC(C)C)-c4ccccc4 | 33000 | 10.1016/j.bmcl.2008.01.007 |
| 676 | O=C(O)c1c(O)c(=O)cc(o1)C(=O)OC(C)(C)C | 36000 | 10.1016/j.bmcl.2004.03.087 |
| 677 | O=C(O)C(=O)/C=C(O)/c1c(cccc1)OCc2ccccc2 | 44000 | 10.1021/jm0504454 |
| 678 | O[C@@H]1[C@@H](CO)O[C@H]([C@@]1(O)C)n2cnc(c23)cnc(n3)N | 46000 | 10.1021/jm030424e |
| 679 | c1cc(CC)ccc1C(=O)CC(=O)C(=O)O | 47000 | 10.1021/jm0342109 |
| 680 | OC[C@H]1O[C@H]([C@@H]([C@@H]1O)OC)n(cn2)c(c23)ncnc3N | 47000 | 10.1021/jm030424e |
| 681 | Cc1coc(C)c1/C(O)=C/C(=O)C(=O)O | 48000 | 10.1021/jm0504454 |
| 682 | c1ccccc1CC[C@]2(OC(=O)CC(=O)C2)C3CCCC3 | 48000 | 10.1016/j.bmcl.2006.06.065 |
| 683 | O=C(O)c(n1)c(O)c(O)nc1-c2sc(C)cc2 | 48600 | 10.1021/jm051064t |
| 684 | c1cccnc1-c(n2)n(c(c23)ccc(c3)C(=O)O)C4CCCCCC4 | 49000 | 10.1016/j.bmcl.2003.10.023 |
| 685 | c1cccc(c12)NC(=NS2(=O)=O)c(c(O)c(c34)cccn4)c(=O)n3C5CCCCC5 | 50000 | 10.1016/j.bmcl.2005.01.071 |
| 686 | O=C(O)C(=O)/C=C(O)/c1occc1 | 50000 | 10.1021/jm0504454 |
| 687 | c1cc(F)ccc1-c(n2)c(-c3ccc(F)cc3)nc(c24)ncnc4NN5[C@@H](C(=O)OC)CCC5 | 51000 | 10.1016/j.bmcl.2004.11.028 |
| 688 | Oc1ccc(cc1)CC[C@]2(OC(=O)CC(=O)C2)C3CCCCC3 | 52000 | 10.1016/j.bmcl.2006.06.065 |
| 689 | c1cccc(c12)cc(o2)-c(n3)n(c(c34)ccc(c4)C(=O)O)C5CCCCC5 | 58000 | 10.1016/j.bmcl.2003.10.023 |
| 690 | c1cocc1-c(n2)c(-c3occc3)nc(c24)ncnc4O | 58000 | 10.1016/j.bmcl.2004.11.028 |
| 691 | C1CCCCC1n(c(n2)C(=O)OCC)c(c23)ccc(c3)C(=O)O | 62000 | 10.1016/j.bmcl.2003.10.023 |
| 692 | c1cc(F)ccc1-c(n2)c(-c3ccc(F)cc3)nc(c24)ncnc4N5CCCCC5 | 68000 | 10.1016/j.bmcl.2004.11.028 |
| 693 | O=C(O)C(=O)/C=C(O)/c1sc(cc1)Cc2ccccc2 | 68000 | 10.1021/jm0504454 |
| 694 | O=c1c(O)c(C(=O)O)nc(n1CC=C)-c2sccc2[N+](=O)[O-] | 69300 | 10.1021/jm051064t |
| 695 | O=C(O)C(=O)/C=C(O)/c1sc(cc1)Cc2cc(F)ccc2 | 74000 | 10.1021/jm0504454 |
| 696 | c1cccnc1-c(n2)n(C3CCC3)c(c24)ccc(c4)C(=O)O | 76000 | 10.1016/j.bmcl.2003.10.023 |
| 697 | c1ccccc1Cn2c(nc(c(c2=O)O)C(=O)O)-c3sccc3[N+](=O)[O-] | 79300 | 10.1021/jm051064t |
| 698 | O=C(O)C(=O)/C=C(O)/c(ccc1)n1Cc2ccc(F)cc2 | 93000 | 10.1021/jm0504454 |
| 699 | O=P(O)(O)O[P@](=O)(O)O[P@@](=O)(O)OC[C@@H]([C@@H](O)[C@@]1(C)O)O[C@@H]1n(cc2-c3ocnn3)c(c24)ncnc4N | 95000 | 10.1016/j.bmcl.2012.05.067 |
| 700 | c1cc(Cl)ccc1COCc(c(=O)c(c23)cc(OC)c(c2)OC)cn3Cc4ccccc4 | 100000 | 10.1016/j.bmcl.2010.11.068 |
| 701 | C[C@@]1(O)[C@H](O)[C@H](CO)O[C@@H]1n(c(c23)ncnc3N)cc2-c4ccccc4 | 100000 | 10.1016/j.bmcl.2012.05.067 |
| 702 | COc(c1)c(OC)cc(c12)c(=O)c(C(=O)c3ccc(C)cc3)cn2CCc4ccccc4 | 110000 | 10.1016/j.bmcl.2010.11.068 |
| 703 | COc(c1)c(OC)cc(c12)c(=O)c(cn2CC3CC3)C(=O)c4ccc(C)cc4 | 120000 | 10.1016/j.bmcl.2010.11.068 |
| 704 | c1cc(Cl)ccc1CCS(=O)(=O)c(c(=O)c(c23)cc(OC)c(c2)OC)cn3Cc4ccccc4 | 150000 | 10.1016/j.bmcl.2010.11.068 |
| 705 | COc(c1)c(OC)cc(c12)c(=O)c(C(=O)c3ccc(C)cc3)cn2Cc4ccccc4 | 170000 | 10.1016/j.bmcl.2010.11.068 |
| 706 | COc1c(Br)cc(cc1C(C)(C)C)N2CCC(=O)N(C)C2=O | 200000 | 10.1016/j.bmcl.2012.04.017 |
| 707 | c1cc(Cl)ccc1CN(C)C(=O)c(cn2Cc3ccc(Cl)cc3)c(=O)c(c24)cc(F)c(c4)N5CCOCC5 | 330000 | 10.1016/j.bmcl.2010.11.068 |
| 708 | c1cccc(c12)n(CCC(C)C)cc(c2=O)C(=NS3(=O)=O)Nc(c34)cccc4 | >10000 | 10.1021/jm050855s |
| 709 | CCOC(=O)c(cc1)cc(c12)n(CCC(C)C)c(=O)c(c2O)C(=NS3(=O)=O)Nc(c34)cccc4 | >10000 | 10.1021/jm050855s |
| 710 | c1ccc([N+](=O)[O-])c(c12)n(CCC(C)C)c(=O)c(c2O)C(=NS3(=O)=O)Nc(c34)cccc4 | >10000 | 10.1021/jm050855s |
| 711 | c1cccc(c12)nc(O)c(c2O)C(=NS3(=O)=O)Nc(c34)cccc4 | >10000 | 10.1021/jm050855s |
| 712 | c1cc(C(=O)O)cc(c12)n(CCC(C)C)c(=O)c(c2O)C(=NS3(=O)=O)Nc(c34)cccc4 | >10000 | 10.1021/jm050855s |
| 713 | c1cccc(c12)n(CCC(C)C)c(=O)c(c2N)C(=NS3(=O)=O)Nc(c34)cccc4 | >10000 | 10.1021/jm050855s |
| 714 | c1ccccc1CCn(c(c23)cccc2)c(=O)c(c3O)C(=NS4(=O)=O)Nc(c45)cccc5 | >10000 | 10.1021/jm050855s |
| 715 | c1c(I)ccc(c12)n(CCC(C)C)c(=O)c(c2O)C(=NS3(=O)=O)Nc(c34)cccc4 | >10000 | 10.1021/jm050855s |
| 716 | c1cscc1CCn(c(c23)cccc2)c(=O)c(c3O)C(=NS4(=O)=O)Nc(c45)cccc5 | >10000 | 10.1021/jm050855s |
| 717 | c1cc(C(C)(C)C)ccc1Cn(c(c23)cccc2)c(=O)c(c3O)C(=NS4(=O)=O)Nc(c45)cccc5 | >10000 | 10.1021/jm050855s |
| 718 | c1cccc(c12)n(CCCC)c(=O)c(c2O)-c([nH]3)nc(c34)cccc4 | >10000 | 10.1021/jm050855s |
| 719 | c1cc([N+](=O)[O-])cc(c12)n(CCC(C)C)c(=O)c(c2O)C(=NS3(=O)=O)Nc(c34)cccc4 | >10000 | 10.1021/jm050855s |
| 720 | c1cc(Br)cc(c12)n(CCC(C)C)c(=O)c(c2O)C(=NS3(=O)=O)Nc(c34)cccc4 | >10000 | 10.1021/jm050855s |
| 721 | c1cccc(c12)n(CCCC(=O)N)c(=O)c(c2O)C(=NS3(=O)=O)Nc(c34)cccc4 | >10000 | 10.1021/jm050855s |
| 722 | C1CCCN1CCn(c(c23)cccc2)c(=O)c(c3O)C(=NS4(=O)=O)Nc(c45)cccc5 | >10000 | 10.1021/jm050855s |
| 723 | c1cccc(c12)n(CCC(C)C)c(=O)c(c2)C(=NS3(=O)=O)Nc(c34)cccc4 | >10000 | 10.1021/jm050855s |
| 724 | O=C(O)c1oc(cc(=O)c1O)C(=O)N(CC)CC | >100000 | 10.1016/j.bmcl.2004.03.087 |
| 725 | COC(=O)c1c(O)c(=O)cc(o1)C(=O)O | >100000 | 10.1016/j.bmcl.2004.03.087 |
| 726 | c1cc(OC)ccc1-c(n2)c(-c3ccc(cc3)OC)nc(c24)ncnc4O | >100000 | 10.1016/j.bmcl.2004.11.028 |
| 727 | c1ccccc1-c(n2)c(-c3ccccc3)nc(c24)ncnc4NCCc(cc5)ccc5F | >100000 | 10.1016/j.bmcl.2004.11.028 |
| 728 | c1ccc(OC)cc1-c(n2)c(-c3cc(OC)ccc3)nc(c24)ncnc4O | >100000 | 10.1016/j.bmcl.2004.11.028 |
| 729 | c1cc(C)ccc1-c(n2)c(-c3ccc(C)cc3)nc(c24)ncnc4O | >100000 | 10.1016/j.bmcl.2004.11.028 |
| 730 | n1cnc(O)c(n2)c1nc(c23)c4c(cccc4)c5c3cccc5 | >100000 | 10.1016/j.bmcl.2004.11.028 |
| 731 | c1ccccc1-c(n2)c(-c3ccccc3)nc(c24)ncnc4N5CCCCC5 | >100000 | 10.1016/j.bmcl.2004.11.028 |
| 732 | c1ccccc1C(=O)N/C(C(=O)O)=C\c(cnc2)cc2-c3c(Cl)cccc3 | >100000 | 10.1016/j.bmcl.2005.03.066 |
| 733 | c1ccccc1C(=O)N/C(C(=O)O)=C\c(cc2)oc2-c3ccc(cc3)CO | >100000 | 10.1016/j.bmcl.2005.03.066 |
| 734 | O=S(=O)(N)c1ccc(cc1)-c2oc(cc2)/C=C(C(=O)O)\NC(=O)c3ccccc3 | >100000 | 10.1016/j.bmcl.2005.03.066 |
| 735 | c1ccccc1C(=O)NC(\C(=O)Nc2ccccc2)=C/c3ccc(cc3)Oc4c(Br)cccc4 | >100000 | 10.1016/j.bmcl.2005.03.106 |
| 736 | c1ccccc1CNC(=O)C(\NC(=O)c2ccccc2)=C\c3ccc(cc3)Oc4c(Br)cccc4 | >100000 | 10.1016/j.bmcl.2005.03.106 |
| 737 | Oc1ccc(cc1)CC[C@@]2(C)OC(=O)CC(=O)C2 | >100000 | 10.1016/j.bmcl.2006.06.065 |
| 738 | c1ccccc1[C@@H]([C@H](C2)C(=O)O)N([C@@]2(C(=O)O)CCC(=O)O)C(=O)c3ccc(C(F)(F)F)cc3 | >20000 | 10.1016/j.bmcl.2005.01.076 |
| 739 | c1ccsc1[C@@H]([C@H](C2)C(=O)O)N([C@@]2(C(=O)O)CC(C)C)C(=O)c3c(C(F)(F)F)cccc3 | >20000 | 10.1016/j.bmcl.2005.01.076 |
| 740 | c1cc(C(F)(F)F)ccc1C(=O)N([C@H]([C@H](C2)C(=O)O)c3ccccc3)[C@@]2(C(=O)O)Cc4ccc(O)cc4 | >20000 | 10.1016/j.bmcl.2005.01.076 |
| 741 | c1ccsc1[C@@H]([C@H](C2)C(=O)O)N([C@@]2(C(=O)O)CC(C)C)C(=O)CCCCCC | >20000 | 10.1016/j.bmcl.2005.01.076 |
| 742 | c1cccc(c12)oc3c2CCO[C@@]3(CCC)CC(=O)O | >20000 | 10.1016/j.bmcl.2005.08.114 |
| 743 | c1cccc(c12)sc3c2CCO[C@]3(CCC)CC(=O)O | >20000 | 10.1016/j.bmcl.2005.08.114 |
| 744 | O=C(O)[C@@H]1CCCN1S(=O)(=O)c2c(N)cccc2 | >20000 | 10.1021/jm060168g |
| 745 | c1cccc(c12)n(CCC(C)C)c(=O)c(c2O)C(=NS3(=O)=O)N(C)c(c34)cccc4 | >2429 | 10.1021/jm050855s |
| 746 | O=C(O)c(n1)c(O)c(O)nc1C2CCCCC2 | >25000 | 10.1021/jm051064t |
| 747 | O=C(O)CN(C(=O)OC)C(=O)c(ccc1)c(c12)ccc(c2C(F)(F)F)OC | >26000 | 10.1016/j.bmcl.2004.06.013 |
| 748 | c1cc(C(=O)O)oc1-c(n2)n(c(c23)ccc(c3)C(=O)O)C4CCCCC4 | >30000 | 10.1016/j.bmcl.2003.10.023 |
| 749 | c1cc(CC)sc1-c(n2)n(c(c23)ccc(c3)C(=O)O)C4CCCCC4 | >30000 | 10.1016/j.bmcl.2003.10.023 |
| 750 | c1c(C)c(C)oc1-c(n2)n(c(c23)ccc(c3)C(=O)O)C4CCCCC4 | >30000 | 10.1016/j.bmcl.2003.10.023 |
| 751 | O=C(O)c(c1)ccc(c12)n(C3CCCCC3)c(n2)-c4c(n[nH]c4)-c5ccccc5 | >30000 | 10.1016/j.bmcl.2003.10.023 |
| 752 | c1cc(CO)oc1-c(n2)n(c(c23)ccc(c3)C(=O)O)C4CCCCC4 | >30000 | 10.1016/j.bmcl.2003.10.023 |
| 753 | O=C1N(N)C(=S)S/C1=C\c2ccccc2 | >30000 | 10.1021/jm050859x |
| 754 | c1cc(Cl)ccc1/C=C2\SC(=S)N(C2=O)Cc3ccccc3 | >30000 | 10.1021/jm050859x |
| 755 | O=C1N(N)C(=S)S/C1=C\c2ccc(cc2)N(C)C | >30000 | 10.1021/jm050859x |
| 756 | O=C1N(N)C(=S)S/C1=C\c2ccc(cc2)OC | >30000 | 10.1021/jm050859x |
| 757 | N#Cc1ccc(C(=O)N)c(c12)[nH]c3c2CCC[C@]3(CCC)CC(=O)O | >33000 | 10.1016/j.bmcl.2006.01.105 |
| 758 | c1cccc(c12)NC(=NS2(=O)=O)c(c3O)c(=O)n(c(c34)cccc4)NCc5ccc(C)cc5 | >5000 | 10.1016/j.bmcl.2005.01.071 |
| 759 | O=C(O)C(=O)CC(=O)c1ccncc1 | >50000 | 10.1021/jm0342109 |
| 760 | CC(=O)CC(=O)C(=O)O | >50000 | 10.1021/jm0342109 |
| 761 | c1cc(C(C)(C)C)ccc1C(=O)CC(=O)C(=O)O | >50000 | 10.1021/jm0342109 |
| 762 | OC[C@H]1O[C@@H]([C@@H](O)[C@]1(O)C)n2cnc(c23)c(N)ncn3 | >50000 | 10.1021/jm030424e |
| 763 | COc1c(O)nc(nc1C(=O)O)-c2ccc(Cl)cc2 | >50000 | 10.1021/jm0494669 |
| 764 | O=C(O)c1nc(nc(c1O)OC)-c2cc(O)ccc2 | >50000 | 10.1021/jm0494669 |
| 765 | n1cc(C(=O)O)sc1N(C(CC2)CCS2(=O)=O)C(=O)c3ccc(cc3)Oc4ccccc4 | >50000 | 10.1016/j.bmcl.2004.10.024 |
| 766 | s1cc(C(=O)O)nc1N(C(C)C)C(=O)c2ccc(cc2)Oc3ccccc3 | >50000 | 10.1016/j.bmcl.2004.10.024 |
| 767 | OC(=O)c(n1)c(O)c(O)nc1Cc2ccccc2 | >50000 | 10.1021/jm051064t |
| 768 | OC(=O)c(c1)ccc(c12)cc(C3CCCCC3)c(n2)-c4ccccc4 | >50000 | 10.1016/j.bmcl.2006.05.012 |
| 769 | c1ccccc1-c(nc(c23)cc([nH]3)C(=O)O)n2C4CCCCC4 | >50000 | 10.1016/j.bmcl.2006.05.012 |
| 770 | COc1ccc(cc1)CC[C@@]2(CCC)OC(=O)CC(=O)C2 | >50000 | 10.1016/j.bmcl.2006.06.065 |
| 771 | OCCNc1ccc(c2c13)CN(Cc2ccc3)c4cccc(Br)c4 | >50000 | 10.1021/jm900517t |
| 772 | c1cccc(c12)NC(=NS2(=O)=O)C3=C(O)[C@@H](N(C3=O)CCC(C)(C)C)c4ccc(F)cc4 | >748 | 10.1016/j.bmcl.2006.01.034 |
