# Supplementary material for: Discovery of Novel Hepatitis C Virus NS5B Polymerase Inhibitors by Combining Random Forest, Multiple e-Pharmacophore Modeling and Docking
Source: PLoS One. 2016 Feb 4;11(2):e0148181. doi: 10.1371/journal.pone.0148181 (PMC4742222; doi:10.1371/journal.pone.0148181)
Supplement: S2 Table — (DOC) [file pone.0148181.s007.doc]

**S2 Table. Structures of the 141 Compounds (in SMILE format) used for the validation set of the RF model together with their experimental bioactivities (in nM).**

| No. | Structure | Activity IC_50_ (nM) | Reference (DOI) |
| --- | --- | --- | --- |
| 1 | C[C@@H]1CC[C@H](CC1)c2nocc2-c3cc(sc3C(=O)O)-c4ccccc4 | 0.11 | 10.1016/j.bmcl.2010.06.008 |
| 2 | Cc1ccc(cc1)-c2cc(=O)[nH]n2-c3cc(sc3C(=O)O)-c4ccccc4 | 0.62 | 10.1016/j.bmcl.2010.06.008 |
| 3 | O=c1[nH]cccc1-c(c(c23)cc(CC)cc2)c(C(=O)NS(=O)(=O)C4CC4)n3Cc5cc(S(=O)(=O)C)c(Cl)cc5F | 2 | 10.1021/jm201322r |
| 4 | O=c1[nH]cccc1-c2c(C(=O)NS(=O)(=O)C(C)C)n(c(c23)ccc(c3)C)Cc4cc(N([O-])O)ccc4F | 4 | 10.1021/jm201322r |
| 5 | c1cc(O)ccc1-c([nH]2)c(c(c23)ccc(c3)C(=O)O)C4CCCCC4 | 4.8 | 10.1021/jm049122i |
| 6 | O=c1[nH]cccc1-c(c(c23)cc(CC)cc2)c(C(=O)NS(=O)(=O)C(C)C)n3Cc4cc(F)ccc4F | 5 | 10.1021/jm201258k |
| 7 | O=S(=O)(C)Nc(cc1)cc(c12)S(=O)(=O)N=C(N2)c(c(O)n(c34)CCCC3)c(=O)c4Cc(cc5)ccc5F | 5 | 10.1016/j.bmcl.2009.05.021 |
| 8 | O=c1[nH]cccc1-c(c(c23)cc(CC)cc2)c(C(=O)NS(=O)(=O)C4CC4)n3Cc5cc(S(=O)(=O)N)c(Cl)cc5Cl | 6 | 10.1021/jm201322r |
| 9 | c1cccc(c12)ncc(c2Cl)Cn(c(c34)cc(F)c(c3)C)c(C(=O)O)c4-c5c(=O)[nH]ccc5 | 6 | 10.1016/j.bmcl.2013.10.060 |
| 10 | c1coc2c1c(F)cc(c23)n(c(C(=O)O)c3-c4c(=O)[nH]ccc4)Cc(c(F)c5)cc(c56)c(N)nn6C | 6 | 10.1016/j.bmcl.2013.11.007 |
| 11 | O=C(O)COc(c1)ccc(c12)NC(=NS2(=O)=O)[C@H](C3=O)C(=O)N(CCC(C)C)c(c34)cccc4 | 8 | 10.1016/j.bmcl.2009.05.091 |
| 12 | c1cccc(c12)n(NC3CCC3)c(=O)c(c2O)C(NS4(=O)=O)=Nc(c45)ccc(c5)NS(=O)(=O)NC | 8.7 | 10.1016/j.bmcl.2006.04.015 |
| 13 | c1cc([NH3+])cc(c12)S(=O)(=O)N=C(N2)c(c3O)c(=O)n(CCC(C)C)c(c34)cccc4 | 13 | 10.1016/j.bmcl.2009.05.091 |
| 14 | CC(C)(C)CCN([C@H]1C(C)(C)C)C(=O)C(=C1O)C2=NS(=O)(=O)c(c23)c(ccc3)CNS(=O)(=O)C | 18 | 10.1016/j.bmcl.2008.05.083 |
| 15 | CS(=O)(=O)Nc1cccc(c1)S(=O)(=O)NC(=O)c(c2-c3ccc[nH]c3=O)n(c(c24)ccc(Cl)c4)Cc5ccccc5F | 18 | 10.1016/j.bmcl.2011.10.041 |
| 16 | Fc1ccc(cc1)CCn2c(=O)c(c(O)n(c23)ncc3)C(=NS4(=O)=O)Nc(c45)ccc(c5)NS(=O)(=O)C | 18 | 10.1016/j.bmcl.2009.05.022 |
| 17 | OC(=O)COC(=CC=1)CC(C12)=C(C=N2)C[C@@H](C(=O)O)NC(=O)c(cc3)cc(c34)nc(-c5cocc5)n4C6CCCCC6 | 19 | 10.1016/j.bmcl.2009.10.136 |
| 18 | Cc1ccc(cc1)S(=O)(=O)NC(=O)c(c2-c3ccc[nH]c3=O)n(c(c24)ccc(Cl)c4)Cc5ccnc(N)c5 | 19 | 10.1016/j.bmcl.2011.10.041 |
| 19 | C1CCCCC1c2c(-c(cc3)ccc3F)cnc(n24)c(cn4)C(=O)N[C@H](C(=O)O)Cc5c[nH]c(c56)cccc6 | 20 | 10.1016/j.bmcl.2009.09.087 |
| 20 | C1CCCCC1n(c(c23)ccc(c2)C(=O)O)c(n3)-c4ccc(cc4F)OCc5cc(N(C(=O)C)C(C)C)ccc5-c6ccc(Cl)cc6 | 21 | 10.1021/jm060269e |
| 21 | CN1CCN(CC1)c(c2)c(F)cc(c23)c(=O)c(cn3Cc4ccc(Cl)cc4)-c5noc(n5)Cc6ccccc6 | 23 | 10.1016/j.bmcl.2011.11.013 |
| 22 | COCCNc1ccc(c2c13)C(=O)N(C(=O)c2ccc3)c4cccc(Br)c4 | 24 | 10.1021/jm900517t |
| 23 | Nc(c1)nccc1Cn(c(c23)ccc(Cl)c3)c(C(=O)NS(=O)(=O)C)c2-c4c[nH]c(=O)[nH]c4=O | 27 | 10.1016/j.bmcl.2011.10.041 |
| 24 | O=S(=O)(C)Nc(cc1)cc(c12)S(=O)(=O)NC(=N2)c(c3O)c(=O)n(CCC(C)C)nc3CCC | 29 | 10.1016/j.bmcl.2008.02.072 |
| 25 | C1CCCCC1c(c(cc2)c3cc24)c5n3[C@@H](C=Cc6c5ccc(c6)OC)C(=O)N(C)CCOCCN(C)S(=O)(=O)NC4=O | 31 | /10.1002/anie.201200110 |
| 26 | OC(=O)/C=C/c1ccc(cc1)NC(=O)C2(CCCC2)NC(=O)c(cc3)cc(c34)n5c(c6c(CCC5)cccc6)c4C7CCCCC7 | 34 | 10.1016/j.bmcl.2012.02.063 |
| 27 | O=S(=O)(C)Nc(cc1)cc(c12)S(=O)(=O)C=C(N2)c(c3O)c(=O)n(CCC(C)C)c(c34)ccc(F)c4 | 41 | 10.1016/j.bmcl.2009.05.004 |
| 28 | c1cc(C(=O)O)cc(c12)c(C)c(-c3ccoc3)n2C4CCCCC4 | 44 | 10.1016/j.bmcl.2006.07.074 |
| 29 | c1ccccc1CN([C@H]([C@@H]23)CCC3)C(=O)C(=C2O)C(N4)=NS(=O)(=O)c(c45)cc(cc5)NS(=O)(=O)C | 46 | 10.1016/j.bmcl.2008.11.048 |
| 30 | c1ccc(O)c(c12)NC3=C(C(=O)CC(C3)(C)C)[C@@H](N2C(=O)c4nn(C)c(C)c4)c(c5F)cccc5OCc6ccccc6 | 50 | 10.1016/j.bmcl.2009.03.035 |
| 31 | s1cccc1-c(c2O)nn(CC3CCC3)c(=O)c2C(N4)=NS(=O)(=O)c(c45)cc(cc5)OCC(=O)N | 55 | 10.1016/j.bmcl.2008.01.007 |
| 32 | O=C(O)C(=O)/C=C(O)/c1cc(ccc1)OCc(c2C#N)cc(Cl)c(Cl)c2 | 56 | 10.1021/jm0504454 |
| 33 | O=C(O)c(n1)c(O)c(O)nc1-c2sccc2N([O-])O | 65 | 10.1021/jm051064t |
| 34 | C1CCCCC1Nc2ccc(c3c24)C(=O)N(C(=O)c3ccc4)c5cccc(Br)c5 | 66 | 10.1021/jm900517t |
| 35 | O=C(O)c(c1)ccc(c12)c(C3CCCCC3)c(C4CCCCC4)n2CC(=O)N5CCC(CC5)N(C)C | 72 | 10.1021/jm050056+ |
| 36 | C1CC(C)(C)Cc(c12)sc(c2C(=O)OCC)NC(=O)NS(=O)(=O)c3n(C)ccn3 | 80 | 10.1016/j.bmcl.2005.09.047 |
| 37 | c1cccc(c12)n(CCCCC)c(=O)c(c2O)C(=NS3(=O)=O)Nc(c34)cccc4 | 80 | 10.1021/jm050855s |
| 38 | O=C(O)[C@@H]1CCCN1S(=O)(=O)c2c(O)c(Cl)c(Cl)c(Cl)c2 | 80 | 10.1021/jm060168g |
| 39 | CS(=O)(=O)Nc(c1)ccc(c12)NC(=NS2(=O)=O)C(=C3O)C(=O)N(CC3(C)C)Cc(cc4)ccc4F | 85 | 10.1016/j.bmcl.2009.09.051 |
| 40 | C1CCCN1c(c2O)nn(CCC(C)(C)C)c(=O)c2C(N3)=NS(=O)(=O)c(c34)cc(cc4)OS(=O)(=O)C | 86 | 10.1016/j.bmcl.2008.08.094 |
| 41 | c1cc(C(=O)O)cc(c12)n(C)c(-c3ccccn3)c2C4CCCCC4 | 90 | 10.1016/j.bmcl.2006.07.074 |
| 42 | O=C1NS(=O)(=O)N(C)CCCCN(C)C(=O)COc(cccc2)c2c(n(C)c(c3)c4ccc13)c4C5CCCCC5 | 97 | 10.1016/j.bmcl.2012.03.097 |
| 43 | C1CC(C)(C)Cc(c12)sc(c2C(=O)OCC)NC(=O)NS(=O)(=O)N(CC3)Cc(c34)cccc4 | 100 | 10.1016/j.bmcl.2005.09.047 |
| 44 | C1COCCN1CCn2c(-c3ccccc3)c(c(c24)ccc(c4)C(=O)O)C5CCCCC5 | 111 | 10.1021/jm049122i |
| 45 | c1cccnc1-c(n2)n(C3CCCCC3)c(c24)ccc(c4)C(=O)N[C@H](C(=O)O)Cc5c[nH]c(c56)ccc(c6)O | 140 | 10.1016/j.bmcl.2003.12.032 |
| 46 | c1c(Cl)cc(Cl)cc1S(=O)(=O)Nc(ccc2)cc2\C(O)=C\C(=O)C(=O)O | 140 | 10.1021/jm0504454 |
| 47 | COc1ccc(cc1Cl)CC[C@@]2(C3CCCC3)OC(O)=C(C(=O)C2)Sc4n(C)ccn4 | 140 | 10.1016/j.bmcl.2006.06.065 |
| 48 | OC(=O)c(c1)ccc(c12)c(C3CCCCC3)c4n2C[C@H]5[C@H](N(C)CC5)c6c4cccc6 | 140 | 10.1021/jm0610245 |
| 49 | c1cccc(c12)c(O)c(cc2CCC(C)C)C(=NS3(=O)=O)Nc(c34)ccc(c4)NS(=O)(=O)C | 150 | 10.1016/j.bmcl.2009.05.063 |
| 50 | C1CCCCC1c2c(-c3ccccn3)n(C)c(c24)cc(cc4)C(=O)NC(C)(C)C(=O)Nc5ccc(cc5)-c6nc(cs6)C(=O)O | 154 | 10.1016/j.bmcl.2011.04.082 |
| 51 | CC(C)CCn1c(=O)c(c(O)c(n12)ccc2)C(N3)=NS(=O)(=O)c(c34)cc(cc4)NS(=O)(=O)C5CC5 | 160 | 10.1016/j.bmcl.2008.04.066 |
| 52 | Brc1ccc(C)c(c12)sc3c2CCO[C@]3(CCC)CC(=O)O | 190 | 10.1016/j.bmcl.2005.08.114 |
| 53 | O=C(C1)NCCCCCN(C)S(=O)(=O)NC(=O)c(c2)ccc(c2n13)c(C4CCCCC4)c3-c5ccc(Cl)cc5 | 190 | 10.1016/j.bmcl.2012.03.097 |
| 54 | C1CCCCC1C(=O)N/C(C(=O)O)=C\c2ccc(cc2)Oc3c(Br)cccc3 | 200 | 10.1016/j.bmcl.2005.03.106 |
| 55 | c1ccccc1/C=C(C)/C=C2\SC(=S)N(C2=O)NS(=O)(=O)c3ccccc3 | 200 | 10.1021/jm050859x |
| 56 | O=C(O)c(n1)c(O)c(O)nc1-c2sccc2NC(=O)Cc3c[nH]c(c34)cccc4 | 200 | 10.1021/jm051064t |
| 57 | OC(=O)/C=C\c1ccc(cc1)NC(=O)C2(CCC2)NC(=O)c(cc3)cc(c34)nc(-c5ccccc5)n4C6CCCCC6 | 200 | 10.1016/j.bmcl.2010.02.003 |
| 58 | Clc1cc(Cl)cc(c1)Nc2c(C#N)c(ns2)O | 200 | 10.1016/j.bmcl.2006.10.002 |
| 59 | CC(C)C[C@@]1(C(=O)O)C[C@H](C(=O)NS(=O)(=O)C(F)(F)F)[C@H](c2cccs2)N1C(=O)c3ccc(C(F)(F)F)cc3 | 200 | 10.1016/j.bmcl.2007.01.034 |
| 60 | O=C(O)c(n1)c(O)c(O)nc1-c2sccc2NC(=O)Cc3c(Cl)cccc3 | 210 | 10.1021/jm051064t |
| 61 | C1=CC(O)=C[C@@H](C12)C(=CN=2)C[C@@H](C(=O)O)NC(=O)c(cc3)cc(c34)nc(-c5cocc5)n4C6CCCCC6 | 220 | 10.1016/j.bmcl.2010.02.003 |
| 62 | c1cccc(c12)C(CCCC)(CCCC)C(=O)C(=C2O)C(=NS3(=O)=O)Nc(c34)ccc(c4)OCC(=O)N | 260 | 10.1016/j.bmcl.2008.06.043 |
| 63 | C1CCCCC1c2c(-c3cocc3)n(CC)c(c24)cc(cc4)C(=O)NC(C)(C)C(=O)Nc5ccc(cc5)/C=C/C(=O)O | 282 | 10.1016/j.bmcl.2011.04.059 |
| 64 | c1cc(C(=O)O)c(Cl)c(c12)n(C)c(-c3ccoc3)c2C4CCCC4 | 290 | 10.1016/j.bmcl.2006.07.074 |
| 65 | CCC[C@@](CC(=O)O)(OCC1)C(=C12)N=C3[C@@H]2C(C#N)=CC(=C3C)OCCc4ccncc4 | 310 | 10.1016/j.bmcl.2011.04.052 |
| 66 | C1C[C@H](C)CC[C@H]1C(=O)N(C(C)C)c2c(C(=O)O)cc(cc2)Oc3ccccc3C4CC4 | 330 | 10.1016/j.bmcl.2013.09.102 |
| 67 | c1cc(F)ccc1CN(C2=O)[C@@H](C(C)(C)C)C(O)=C2C(=CS3(=O)=O)Nc(c34)ccc(F)c4 | 338 | 10.1016/j.bmcl.2009.08.023 |
| 68 | O=C(O)c(c1)ccc(c12)n(C3CCCCC3)c(n2)-c4ccc(cc4)OCCc5ccccc5-c6ccccc6 | 340 | 10.1016/j.bmcl.2006.01.032 |
| 69 | CC(C)(C)C#Cc(sc1C(=O)O)cc1N(C(=O)[C@@H](CC2)CC[C@H]2C)N(C)c3ccncc3 | 346 | 10.1016/j.bmcl.2012.05.025 |
| 70 | O=C(O)C(=O)CC(=O)c1c(cccc1)OCCCC#N | 350 | 10.1021/jm0342109 |
| 71 | FC(F)(F)Oc1ccccc1Oc(cc2)cc(C(=O)O)c2NS(=O)(=O)c(cc3)ccc3C | 370 | 10.1016/j.bmcl.2013.09.102 |
| 72 | Fc1cc(Cl)ccc1Cn2cnc(=O)c(c23)cc(cc3)Oc4ncccc4C(F)(F)F | 400 | 10.1016/j.bmcl.2013.05.037 |
| 73 | n1nn[nH]c1-c(cn2)c(n23)ncc(c3C4CCCCC4)-c(cc5)ccc5Oc6ccccc6 | 400 | 10.1016/j.bmcl.2009.09.087 |
| 74 | C1CC[C@H]([C@H]12)N(CCC(C)C)C(=O)C(=C2O)C(N3)=NS(=O)(=O)c(c34)cc(cc4)NS(=O)(=O)C | <10 | 10.1016/j.bmcl.2008.11.048 |
| 75 | c1ccsc1[C@@H]([C@H](C2)C(=O)O)N([C@@]2(C(=O)O)CC(C)C)C(=O)c3cc(Cl)c(Cl)cc3 | 600 | 10.1016/j.bmcl.2005.01.076 |
| 76 | c1cc(Cl)c(Cl)cc1/C(C)=C2\SC(=S)N(C2=O)NS(=O)(=O)c3ccccc3 | 600 | 10.1021/jm050859x |
| 77 | c1cc(F)c(Cl)cc1CN2C(=O)C(=C(O)C23CC3)C4=NS(=O)(=O)c(c45)c(ccc5)CN(C)S(=O)(=O)C | 620 | 10.1016/j.bmcl.2008.05.083 |
| 78 | C1CCCc(c12)sc(c2C(=O)OCC)NC(=O)NS(=O)(=O)c3cccc(c34)cccc4 | 700 | 10.1016/j.bmcl.2005.09.047 |
| 79 | c1cc(Cl)c(Cl)cc1/C=C2\SC(=S)N([C@@H]2O)NS(=O)(=O)c3ccc(F)cc3 | 700 | 10.1021/jm050859x |
| 80 | Clc1ccc(Cl)c(c12)sc3c2CCO[C@]3(CC(=O)O)CCCC | 750 | 10.1016/j.bmcl.2005.08.114 |
| 81 | OCCNc1ccc(c2c13)C(=O)N(Cc2ccc3)c4cccc(Br)c4 | 850 | 10.1021/jm900517t |
| 82 | FC(F)(F)c1c(OC)ccc(c12)c(ccc2)C(=O)N(CC(=O)O)CC(=O)O | 900 | 10.1016/j.bmcl.2004.06.013 |
| 83 | O=C(C)COc(cc1)ccc1NC(=O)[C@H](C)NC(=O)c(cc2)cc(c23)nc(-c4cocc4)n3C5CCCCC5 | 900 | 10.1016/j.bmcl.2009.10.136 |
| 84 | c1ccccc1Cn(c(c23)ccc(Cl)c3)c(C(=O)O)c2-c4ccccc4F | 900 | 10.1021/jm201258k |
| 85 | CN(C)Cc(cc1)ccc1-c(n2CC(=O)N(C)C)c(c(c23)ccc(c3)C(=O)O)C4CCCCC4 | 920 | 10.1021/jm050056+ |
| 86 | CC(C)(C)c1cc(N)c(C)cc1SC(C(O2)=O)=C(O)C[C@]2(C3CCCC3)CCc4ccc(O)cc4 | 920 | 10.1128/AAC.01008-06 |
| 87 | c1ccnc(c12)n(CCC(C)C)c(=O)c(c2O)C(NS3(=O)=O)=Nc(c34)ccc(c4)OCCC | 930 | 10.1016/j.bmcl.2006.04.022 |
| 88 | CCC[C@@](CC(=O)O)(OCC1)C(=C12)N=C3[C@@H]2C(C#N)=CC(=C3C)C(=O)NCc4ccccc4 | 980 | 10.1016/j.bmcl.2011.04.052 |
| 89 | O=C(O)c1c(O)c(=O)n(C)c(n1)-c2cc(O)ccc2 | 1000 | 10.1021/jm0494669 |
| 90 | COc1ccc(cc1Cl)CC[C@]2(OC(=O)CC(=O)C2)C3CCCC3 | 1000 | 10.1016/j.bmcl.2006.06.065 |
| 91 | COc(c1)c(OC)cc(c12)c(=O)c(C(=O)c3ccc(C)cc3)cn2Cc4ccc(Cl)c(Cl)c4 | 1100 | 10.1016/j.bmcl.2011.11.068 |
| 92 | O=C(O)c(c1)ccc(c12)n(C3CCCC3)c(n2)-c4ccc(cc4)OCc5ccc(Cl)c(Cl)c5 | 1300 | 10.1016/j.bmcl.2006.01.032 |
| 93 | O=C(O)c(n1)c(O)c(O)nc1-c2sccc2NS(=O)(=O)c(cc3)cc(c34)cccc4 | 1420 | 10.1021/jm051064t |
| 94 | O=C(O)c1ncnc(c12)n(c(n2)-c3cocc3)C4CCCCC4 | 1600 | 10.1016/j.bmcl.2004.11.028 |
| 95 | s1c(C)ccc1-c2nn(Cc3ccccc3)c(=O)c(c2O)C(N4)=CS(=O)(=O)c(c45)cc(cc5)NS(=O)(=O)C | 1600 | 10.1016/j.bmcl.2008.07.014 |
| 96 | O=C(O)c(c1)cccc1NC(=O)[C@H](C)NC(=O)c(cc2)cc(c23)nc(-c4cocc4)n3C5CCCCC5 | 1610 | 10.1016/j.bmcl.2009.10.136 |
| 97 | n1c(N)nc(O)c(c12)ncn2[C@H]3[C@H](O)[C@H](F)[C@H](O3)CO | 1800 | 10.1021/jm030424e |
| 98 | O=C(O)C(=O)/C=C(O)/c1c(C)cc(C)cc1 | 2000 | 10.1021/jm0504454 |
| 99 | CC(C)CCn1c(=O)c(c(O)c(n12)ccc2)C(N3)=NS(=O)(=O)c(c34)cc(cc4)OC | 2200 | 10.1016/j.bmcl.2008.04.066 |
| 100 | c1cccc(c12)NC(=NS2(=O)=O)C3=C(O)[C@H](Cc4cnccc4)N(C3=O)Cc5ccc(F)cc5 | 2466 | 10.1016/j.bmcl.2006.01.034 |
| 101 | C1CCCCC1N(c2sc(cn2)C(=O)O)C(=O)c3ccc(cc3)Oc(cc4Cl)ccc4Cl | 2600 | 10.1016/j.bmcl.2004.10.024 |
| 102 | CCCNC(=O)C(=CC=1C#N)[C@@H](C)C2=NC(=C3C12)[C@](OCC3)(CCC)CC(=O)O | 3000 | 10.1016/j.bmcl.2011.04.052 |
| 103 | c1cc(C(=O)O)cc(c12)cc(C3CCCCC3)c(n2)-c4ccccc4 | 4800 | 10.1016/j.bmcl.2006.05.012 |
| 104 | Cc1cc(Cl)c(C)cc1S(=O)(=O)Nc(c2C(=O)O)cc(s2)-c3ccccc3 | 5000 | 10.1016/j.bmcl.2004.08.018 |
| 105 | c1ccccc1C(=O)N/C(C(=O)O)=C\c(cc2)oc2-c3ccccc3 | 6700 | 10.1016/j.bmcl.2005.03.066 |
| 106 | O=C(O)C(=O)/C=C(O)/C12C[C@H]3C[C@@H](C1)C[C@@H](C2)C3 | 6700 | 10.1021/jm0504454 |
| 107 | CC(C)(C)c1cc(ccc1O)N2CCC(=O)NC2=O | 7600 | 10.1016/j.bmcl.2012.04.017 |
| 108 | C1CCCCN1c2sc(cc2)\C(O)=C\C(=O)C(=O)O | 8200 | 10.1021/jm0504454 |
| 109 | O=C(O)[C@H](C1)C[C@@H](C(=O)O)N1C(=O)c2cc(Cl)c(Cl)cc2 | 12000 | 10.1016/j.bmcl.2005.01.076 |
| 110 | C1CCCC1CCC(=O)NC(\C(=O)O)=C/c2ccc(cc2)Oc3c(Br)cc(F)cc3 | 15000 | 10.1016/j.bmcl.2005.03.106 |
| 111 | c1cc(F)ccc1C(=O)c2cn(C)c(c2)\C=C\C(O)=C\C(=O)C(=O)O | 16000 | 10.1021/jm0504454 |
| 112 | O=C(O)C(=O)/C=C(O)/C=C/c1c[nH]c(c12)ccc(Cl)c2 | 18000 | 10.1021/jm0504454 |
| 113 | c1ccc(F)cc1Cn2c(ccc2)\C=C\C(O)=C\C(=O)C(=O)O | 18000 | 10.1021/jm0504454 |
| 114 | O=c1c(O)c(C(=O)O)oc(c12)cccc2 | 19000 | 10.1016/j.bmcl.2004.03.087 |
| 115 | O=C(O)C(=O)/C=C(O)/c1cn(C)c(c1)Cc2cc(F)ccc2 | 21000 | 10.1021/jm0504454 |
| 116 | O=C(O)C(=O)\C=C(O)\c(c1O)cnc(c12)cc(Cl)cc2 | 26000 | 10.1021/jm0504454 |
| 117 | O=C(O)C(=O)/C=C(O)/c(s1)cc(c12)COc3c2cc(F)cc3 | 27000 | 10.1021/jm0504454 |
| 118 | O=C(O)C(=O)CC(=O)c1c(cccc1)OCc2ccccc2 | 44000 | 10.1021/jm0342109 |
| 119 | c1cccc(c1N([O-])O)Oc(cc2)ccc2/C=C(C(=O)O)\NC(=O)c3ccccc3 | 73000 | 10.1016/j.bmcl.2005.03.066 |
| 120 | c1cc(OC)ccc1-c(nc2/C=C/C(=O)O)n(c2)C3CCCCC3 | 77300 | 10.1016/j.bmcl.2006.05.012 |
| 121 | c1cc(F)ccc1-c(n2)c(-c3ccc(F)cc3)nc(c24)ncnc4NCCc(c5OC)cccc5 | 80000 | 10.1016/j.bmcl.2004.11.028 |
| 122 | CCCCS(=O)(=O)N(CCC1)C[C@@]1(\C(O)=C\C(=O)C(=O)O)Cc2ccc(Cl)cc2 | 81400 | 10.1021/jm0504454 |
| 123 | C[C@@]1(O)[C@H](O)[C@H](CO)O[C@@H]1n(c(c23)ncnc3N)cc2-c4ccccc4 | 100000 | 10.1016/j.bmcl.2012.05.067 |
| 124 | c1c(F)ccc(c12)n(CCC3CC3)c(=O)c(c2O)-c(nc4O)nc(c45)cccc5 | >10000 | 10.1021/jm050855s |
| 125 | c1c(F)ccc(c12)n(CC(=O)N(C)C)c(=O)c(c2O)C(=NS3(=O)=O)Nc(c34)cccc4 | >10000 | 10.1021/jm050855s |
| 126 | O=C(O)c1c(O)c(=O)cc(o1)COCc2ccccc2 | >100000 | 10.1016/j.bmcl.2004.03.087 |
| 127 | o1cc(O)c(=O)cc1C(=O)OCC | >100000 | 10.1016/j.bmcl.2004.03.087 |
| 128 | C1CCC[C@H]2[C@@H]1c(c23)nc4c(n3)c(O)ncn4 | >100000 | 10.1016/j.bmcl.2004.11.028 |
| 129 | C1CCCCC1n2nc(/C=C/C(=O)O)cc2-c3ccccn3 | >100000 | 10.1016/j.bmcl.2006.07.074 |
| 130 | c1cc(C(=O)O)cc(c12)S(=O)(=O)C(c3ccoc3)=C2C4CCCC4 | >100000 | 10.1016/j.bmcl.2006.07.074 |
| 131 | O=C(O)[C@@H]1CCCN1S(=O)(=O)c2c(cc(C)c(Cl)c2)NC(=O)c3ccccc3 | >20000 | 10.1021/jm060168g |
| 132 | O=C(O)c(c1)ccc(c12)n(C3CCCCC3)c(n2)-c4oc(cc4)-c5ccc(Cl)cc5 | >30000 | 10.1016/j.bmcl.2003.10.023 |
| 133 | Clc1ccc(cc1)/C=C2\SC(=S)NC2=O | >30000 | 10.1021/jm050859x |
| 134 | c1c(Cl)ccc(c12)[nH]c3c2CCC[C@]3(CCC)CC(=O)O | >33000 | 10.1016/j.bmcl.2006.01.105 |
| 135 | c1ccccc1CCC(=O)C(=O)O | >50000 | 10.1021/jm0342109 |
| 136 | c1ccccc1C(=O)C[C@H](O)C(=O)O | >50000 | 10.1021/jm0342109 |
| 137 | CC(C)(C)C(=O)CC(=O)C(=O)O | >50000 | 10.1021/jm0342109 |
| 138 | n1cc(O)c(O)nc1-c2cc(O)ccc2 | >50000 | 10.1021/jm0494669 |
| 139 | O=C(O)c1nc(sc1)NC(=O)c2ccc(cc2)Oc3ccccc3 | >50000 | 10.1016/j.bmcl.2004.10.024 |
| 140 | C1CCCCC1c2cc(/C=C/C(=O)O)[nH]c2-c3ccccn3 | >50000 | 10.1016/j.bmcl.2006.07.074 |
| 141 | c1cccnc1-c(n2)[nH]c(c23)ccc(c3)C(=O)O | >500000 | 10.1016/j.bmcl.2003.10.023 |
