# Supplementary material for: Discovery of Novel Hepatitis C Virus NS5B Polymerase Inhibitors by Combining Random Forest, Multiple e-Pharmacophore Modeling and Docking
Source: PLoS One. 2016 Feb 4;11(2):e0148181. doi: 10.1371/journal.pone.0148181 (PMC4742222; doi:10.1371/journal.pone.0148181)
Supplement: S3 Table — (DOC) [file pone.0148181.s008.doc]

**S3 Table. Structures of the 116 compounds (in SMILE format) used for the validation of the evaluation of performances of the e-pharmacophore models together with their experimental bioactivities (in nM).**

| No. | Structure | Activity IC_50_ (nM) | Reference (DOI) |
| --- | --- | --- | --- |
| 1 | c1ccccc1-c(sc2C([O-])=O)cc2-c3conc3C4CCCCC4 | 0.074 | 10.1016/j.bmcl.2010.06.008 |
| 2 | c1ccccc1-c(sc2C([O-])=O)cc2-c3cnnn3C4CCCCC4 | 0.34 | 10.1016/j.bmcl.2010.06.008 |
| 3 | c1cccc(c12)NC(=NS2(=O)=O)[C@@H]3C(=O)[C@H](C(C)(C)C)N(C3=O)CCC(C)(C)C | 1.7 | 10.1016/j.bmcl.2006.01.034 |
| 4 | c1coc2c1c(F)cc(c23)n(Cc(c(Cl)c4)cc(c45)cn[nH]5)c(C([O-])=O)c3-c6c(=O)[nH]ccc6 | 3 | 10.1016/j.bmcl.2013.11.007 |
| 5 | c1coc2c1c(F)cc(c23)n(Cc(c(c4)C)cc(c45)cn[nH]5)c(C([O-])=O)c3-c6c(=O)[nH]ccc6 | 3 | 10.1016/j.bmcl.2013.11.007 |
| 6 | COc(c1)c(F)ccc1CN(C2=O)[C@@H](C(C)(C)C)C(=O)[C@H]2C(=CS3(=O)=O)Nc(c34)ccc(c4)[N-]S(=O)(=O)C | 4 | 10.1016/j.bmcl.2009.08.023 |
| 7 | c1ccnc(c12)n(CCC(C)C)c([O-])c(c2=O)C([N-]S3(=O)=O)=Nc(c34)ccc(c4)NS(=O)(=O)CCCC | 5 | 10.1016/j.bmcl.2006.04.022 |
| 8 | CC(C)CCn1c([O-])c(c(=O)n(c12)nc(c2)C)C(=NS3(=O)=O)Nc(c34)ccc(c4)NS(=O)(=O)C | 5 | 10.1016/j.bmcl.2009.05.022 |
| 9 | Fc1ccc(cc1)Cn2c([O-])c(c(=O)n(c23)ncc3)C(=NS4(=O)=O)Nc(c45)ccc(c5)NS(=O)(=O)C | 5 | 10.1016/j.bmcl.2009.05.022 |
| 10 | c1cccc(c12)c(OC)c(c(O)c2CCC(C)C)C(=NS3(=O)=O)Nc(c34)ccc(c4)NS(=O)(=O)C | 5 | 10.1016/j.bmcl.2009.05.063 |
| 11 | O=S(=O)(C)Nc(cc1)cc(c12)S(=O)(=O)N=C(N2)c(c(=O)c3CCC(C)C)c([O-])n(c34)cccc4 | 5 | 10.1016/j.bmcl.2009.05.021 |
| 12 | O=S(=O)(C)Nc(cc1)cc(c12)S(=O)(=O)N=C(N2)c(c(=O)c3CCC(C)C)c([O-])n(c34)cc(F)cc4 | 5 | 10.1016/j.bmcl.2009.05.021 |
| 13 | O=S(=O)(C)[N-]c(cc1)cc(c12)S(=O)(=O)C=C(N2)c(c3=O)c([O-])n(c(c34)ccc(F)c4)Cc5ccc(F)cc5 | 5 | 10.1016/j.bmcl.2009.05.004 |
| 14 | CCC[C@@](CC(=O)[O-])(OCC1)C(=C12)N=C3[C@@H]2C(C#N)=CC(=C3C)OCc4cc(C)on4 | 6 | 10.1016/j.bmcl.2011.04.052 |
| 15 | O=c1[nH]cccc1-c2c(C(\[O-])=N\S(=O)(=O)CC)n(c3ccc(c4c23)cco4)Cc5cc(F)ccc5F | 6 | 10.1016/j.bmc.2013.01.024 |
| 16 | c1ccnc(c12)n(CCC(C)C)c([O-])c(c2=O)C(=NS3(=O)=O)Nc(c34)ccc(c4)[N-]S(=O)(=O)/N=C([O-])\OCCC#N | 6.7 | 10.1016/j.bmcl.2006.04.015 |
| 17 | O=c1[nH]cccc1-c2c(C([O-])=O)n(c3ccc(c4c23)OC(O4)(F)F)Cc5cc(F)ccc5F | 7 | 10.1016/j.bmc.2013.01.024 |
| 18 | CC(C)(C)CCN(C1=O)[C@@H](C(C)(C)C)C(=O)[C@H]1C(=CS2(=O)=O)Nc(c23)ccc(c3)[N-]S(=O)(=O)C | 8 | 10.1016/j.bmcl.2009.08.023 |
| 19 | O=c1[nH]cccc1-c2c(C(\[O-])=N\S(=O)(=O)C)n(c3ccc(c4c23)cco4)Cc5cc(F)ccc5F | 9 | 10.1016/j.bmc.2013.01.024 |
| 20 | CS(=O)(=O)COc(ccc1)c(c12)S(=O)(=O)NC2=C3C(=O)[C@H](C(C)(C)C)N(C3=O)Cc4ccc(F)cc4 | 9 | 10.1016/j.bmcl.2009.08.022 |
| 21 | O=C(N)C(C)(C)Oc(c1)ccc(c12)NC(=NS2(=O)=O)[C@H](C3=O)C(=O)N(c(c34)ccc(F)c4)CCC5CC5 | 9 | 10.1016/j.bmcl.2009.05.091 |
| 22 | c1c(F)ccc(c12)n(CCC3CC3)c([O-])c(c2=O)C(=NS4(=O)=O)Nc(c45)cccc5 | 10 | 10.1021/jm050855s |
| 23 | O=S(=O)(C)[N-]c(cc1)cc(c12)S(=O)(=O)C=C(N2)c(c3=O)c([O-])n(c(c34)ccc(F)c4)Cc5cc(OC)c(F)cc5 | 10 | 10.1016/j.bmcl.2009.05.004 |
| 24 | c1c(F)c(F)cc(c12)nc(Cl)c(c2)Cn(c(c34)cc(F)c(c3)C)c(C([O-])=O)c4-c5c(=O)[nH]ccc5 | 10 | 10.1016/j.bmcl.2013.10.060 |
| 25 | O=S(=O)(C)Nc(c1)ccc(c12)NC(=NS2(=O)=O)c3c(=O)n(CCC(C)C)cc(c3[O-])-c4cccs4 | 11 | 10.1016/j.bmcl.2004.12.030 |
| 26 | O=S(=O)(C)[N-]c(cc1)cc(c12)S(=O)(=O)C=C(N2)c(c3=O)c([O-])n(CCC(C)(C)C)c(c34)ccc(F)c4 | 12 | 10.1016/j.bmcl.2009.05.004 |
| 27 | c1ccnc(c12)n(CCC(C)C)c([O-])c(c2=O)C(=NS3(=O)=O)Nc(c34)ccc(c4)NS(=O)(=O)NCc5cccc(c5)N(O)O | 13 | 10.1016/j.bmcl.2006.04.015 |
| 28 | c1ccsc1-c2nn(CC3(C(F)(F)F)CC3)c(=O)c(c2[O-])C(=N4)NS(=O)(=O)c(c45)cc(cc5)[N-]S(=O)(=O)C | 13 | 10.1016/j.bmcl.2008.02.072 |
| 29 | c1ccsc1-c2nn(CC(C)(C)C)c(=O)c(c2[O-])C(=N3)NS(=O)(=O)c(c34)cc(cc4)[N-]S(=O)(=O)C | 14 | 10.1016/j.bmcl.2008.02.072 |
| 30 | Cc1ccccc1COC(=O)c(c(=O)c(c23)cc(OC)c(c2)OC)cn3Cc4ccc(C(F)(F)F)cc4F | 15 | 10.1016/j.bmcl.2011.11.068 |
| 31 | c1cc(C([O-])=O)cc(c12)n(C)c(-c3ccoc3)c2C4CCCCC4 | 16 | 10.1016/j.bmcl.2006.07.074 |
| 32 | C[NH+]1CCN(CC1)c(c2)c(F)cc(c23)c(=O)c(cn3Cc4ccc(Cl)cc4)-c5noc(n5)Cc6ccc(F)cc6 | 19 | 10.1016/j.bmcl.2011.11.013 |
| 33 | C1CCCCC1c2c(-c(cc3)ccc3F)cnc(n24)c(cn4)C(=O)N[C@H](C(=O)[O-])Cc(cc5)ccc5O | 20 | 10.1016/j.bmcl.2009.09.087 |
| 34 | c1cc(C([O-])=O)cc(c12)n(C)c(-c3ccoc3)c2C4CCCC4 | 21 | 10.1016/j.bmcl.2006.07.074 |
| 35 | [O-]C(=O)c(c1)ccc(c12)c(C3CCCCC3)c4n2C[C@H]5[C@@H](N(CC5)C(=O)C[NH+](C)C)c6c4ccc(c6)OC | 23 | 10.1021/jm0610245 |
| 36 | COc(c1)ccc(c12)NC(=NS2(=O)=O)[C@H](C3=O)C(=O)N(c(c34)ccc(F)c4)CCC5CC5 | 24 | 10.1016/j.bmcl.2009.05.091 |
| 37 | C1CCCCC1c2c(-c(cc3)ccc3F)cnc(n24)c(cn4)C(=O)N[C@H](C(=O)N)Cc(cc5)ccc5O | 24 | 10.1016/j.bmcl.2009.09.087 |
| 38 | c1cc(C([O-])=O)cc(c12)[nH]c(-c3ccoc3)c2C4CCCC4 | 25 | 10.1016/j.bmcl.2006.07.074 |
| 39 | [O-]C(=O)c(c1)ccc(c12)c(C3CCCCC3)c4n2CCOc5c4cccc5 | 26 | 10.1021/jm0610245 |
| 40 | O=S(=O)(C)[N-]c(cc1)cc(c12)S(=O)(=O)NC(=N2)c(c3[O-])c(=O)n(CCC(C)C)nc3C=C(C)C | 29 | 10.1016/j.bmcl.2008.02.072 |
| 41 | c1cc(C([O-])=O)cc(c12)[nH]c(-c3ccoc3)c2C4CCCCC4 | 30 | 10.1016/j.bmcl.2006.07.074 |
| 42 | [O-]C(=O)/C=C/c1ccc(cc1)OC(=O)C2(CCCC2)NC(=O)c(cc3)cc(c34)n5c(c6c(OCC5)cccc6)c4C7CCCCC7 | 30 | 10.1016/j.bmcl.2011.03.067 |
| 43 | O=S(=O)(C)[N-]c(cc1)cc(c12)S(=O)(=O)NC(=N2)c(c3[O-])c(=O)n(CCC(C)C)nc3-c4ccccc4 | 31 | 10.1016/j.bmcl.2008.02.072 |
| 44 | C1CCCCC1c2c(-c3ocnc3)n(C)c(c24)cc(cc4)C(=O)NC(C)(C)C(=O)Nc5ccc(cc5)/C=C/C(=O)[O-] | 34 | 10.1016/j.bmcl.2011.04.059 |
| 45 | C1CCCN1c(c2[O-])nn(CCC3CC3)c(=O)c2C(N4)=NS(=O)(=O)c(c45)cc(cc5)OS(=O)(=O)C | 38 | 10.1016/j.bmcl.2008.08.094 |
| 46 | C1CCCC[C@@H]([C@@H]12)N(CCC(C)(C)C)C(=O)[C@@H](C2=O)C(N3)=NS(=O)(=O)c(c34)cc(cc4)NS(=O)(=O)C | 39 | 10.1016/j.bmcl.2008.11.048 |
| 47 | [O-]C(=O)c(c1)ccc(c12)c(C3CCCCC3)c4n2C[C@H]5[C@@H]([N@@H+](C)CC5)c6c4cccc6 | 40 | 10.1021/jm0610245 |
| 48 | [O-]C(=O)/C=C/c1ccc(cc1)NC(=O)C2(CCCC2)NC(=O)c(cc3)cc(c34)n5c(c6c(ocn6)CCC5)c4C7CCCCC7 | 44 | 10.1016/j.bmcl.2012.02.063 |
| 49 | c1cccc(c12)c3n(CCO2)c4c(c3C5CCCCC5)ccc(c4)C(=O)NC(C)(C)C([O-])=O | 46 | 10.1021/jm0610245 |
| 50 | N#Cc1c(F)cc(C)c(c12)sc3c2CCO[C@@]3(CC([O-])=O)CCC | 50 | 10.1016/j.bmcl.2005.08.114 |
| 51 | c1cc(C)cc(c12)S(=O)(=O)N=C(N2)c(c3=O)c([O-])n(CCC(C)C)c(c34)cccc4 | 50 | 10.1016/j.bmcl.2009.05.091 |
| 52 | c1cc(C([O-])=O)nc(c12)n(C)c(-c3ccoc3)c2C4CCCCC4 | 51 | 10.1016/j.bmcl.2006.07.074 |
| 53 | C[C@@H]1CC[C@H](CC1)C(=O)N(N(C)CCO)c2cc(sc2C(=O)[O-])C#CC(C)(C)C | 52 | 10.1016/j.bmcl.2012.05.025 |
| 54 | COc(cc1)cc(c12)OCC(=O)N(C)CCOCCN(C)S(=O)(=O)/N=C(\[O-])c3ccc4c(c3)n(C)c2c4C5CCCCC5 | 56 | 10.1016/j.bmcl.2012.03.097 |
| 55 | C1CCCCC1c(c(c23)ccc(c2)C([O-])=O)c(n3CC(=O)N(C)C)-c4ccccc4 | 59 | 10.1016/j.bmcl.2006.05.012 |
| 56 | Cn1cccc1-c(n2)n(C3CCCCC3)c(c24)ccc(c4)C(=O)N[C@H](c5cscn5)CC(=CN=6)[C@H](C67)C=C(O)C=C7 | 60 | 10.1016/j.bmcl.2010.02.003 |
| 57 | c1ccc(O)c(c12)NC3=C(C(=O)CC(C3)(C)C)[C@@H](N2C(=O)c4ncncc4)c(c5F)cccc5OCc6ccccc6 | 60 | 10.1016/j.bmcl.2009.03.035 |
| 58 | C1CCCCC1n(c(c23)ccc(c2)C(=O)[O-])c(n3)-c4ccc(cc4F)OCc5cc(N6CCCC6=O)ccc5-c7ccc(Cl)cc7 | 61 | 10.1021/jm060269e |
| 59 | C1CCCCC1c2c(-c(n3)cccc3C)n(C)c(c24)cc(cc4)C(=O)NC(C)(C)C(=O)Nc5ccc(cc5)/C=C/C(=O)[O-] | 61 | 10.1016/j.bmcl.2011.04.059 |
| 60 | O=S(=O)(C)Nc(c1)ccc(c12)NC(=NS2(=O)=O)C(=C3[O-])C(=O)C(CCC(C)C)(CCC(C)C)c(c34)cccc4 | 68 | 10.1016/j.bmcl.2008.06.043 |
| 61 | c1ccccc1C(=O)N/C(C(=O)[O-])=C\c2ccc(cc2)Oc3c(F)cccc3Cl | 70 | 10.1016/j.bmcl.2005.03.066 |
| 62 | C1[C@@H](C2)C[C@H](C3)C[C@@H]2CC13C(=O)N/C(C(=O)[O-])=C\c4ccc(cc4)Oc5c(Br)cccc5 | 70 | 10.1016/j.bmcl.2005.03.106 |
| 63 | CC(C)C[C@@]1(C(=O)[O-])C[C@H](C(=O)[O-])[C@H](c2nccs2)N1C(=O)c3ccc(cc3)C(C)(C)C | 70 | 10.1016/j.bmcl.2007.01.034 |
| 64 | c1ccccc1Cn(c(c23)nccc2)c([O-])c(c3=O)C([N-]S4(=O)=O)=Nc(c45)ccc(c5)OC | 76 | 10.1016/j.bmcl.2006.04.022 |
| 65 | c1ccc(O)c(c12)NC3=C(C(=O)CC(C3)(C)C)[C@@H](N2C(=O)C)c(c(Cl)c4)ccc4Cl | 81 | 10.1016/j.bmcl.2009.03.035 |
| 66 | CC(C)(C)C#Cc(sc1C(=O)[O-])cc1N(C(C)C)C(=O)[C@@H]2CC[C@H](C)CC2 | 86 | 10.1016/j.bmcl.2012.05.025 |
| 67 | c1ccnc(c12)n(CCC(C)C)c([O-])c(c2=O)C([N-]S3(=O)=O)=Nc(c34)ccc(c4)NS(=O)(=O)/N=C([O-])\OCCOC | 87 | 10.1016/j.bmcl.2006.04.015 |
| 68 | C1CCCCC1c2c(-c3ccccn3)n(C)c(c24)cc(cc4)C(=O)NC5(CCC5)C(=O)Nc6ccc(C(=O)[O-])cc6 | 93 | 10.1016/j.bmcl.2011.04.082 |
| 69 | O=S(=O)(C)Nc(c1)ccc(c12)NC(=NS2(=O)=O)C(=C3[O-])C(=O)C(CCC)(CCC)c(c34)cccc4 | 99 | 10.1016/j.bmcl.2008.06.043 |
| 70 | c1cocc1-c(n2)n(C3CCCCC3)c(c24)ccc(c4)C(=O)N[C@H](C(=O)[O-])Cc5c[nH]c(c56)ccc(c6)OC(C(=O)[O-])(C)C | 100 | 10.1016/j.bmcl.2003.12.032 |
| 71 | [O-]C(=O)/C=C/c1ccc(cc1)NC(=O)C2(CCC2)NC(=O)c(cc3)cc(c34)nc(-c5ccccn5)n4C6CCCCC6 | 100 | 10.1016/j.bmcl.2010.02.003 |
| 72 | Fc1cc(F)cc(F)c1Cn2cnc(=O)c(c23)cc(cc3)Oc4c(C(F)(F)F)c(ccn4)OC | 100 | 10.1016/j.bmcl.2013.05.037 |
| 73 | c1cccc(c12)NC(=NS2(=O)=O)c(c3=O)c([O-])n(c(c34)cccc4)[NH2+]CC5CCC5 | 108 | 10.1016/j.bmcl.2005.01.071 |
| 74 | CC(C)CCn1c([O-])c(c(=O)c(n12)ccc2)C(N3)=NS(=O)(=O)c(c34)cc(cc4)N(C)S(=O)(=O)C | 110 | 10.1016/j.bmcl.2008.04.066 |
| 75 | O=C([O-])c(s1)c(C)c(c12)cc(cc2)NC(=O)C3(CCC3)NC(=O)c(cc4)cc(c45)n(C)c(-c6ccccn6)c5C7CCCCC7 | 122 | 10.1016/j.bmcl.2011.04.082 |
| 76 | c1ccc(O)c(c12)NC3=C(C(=O)CC(C3)(C)C)[C@@H](N2C(=O)C(C)C)c(c4C)cccc4OCc5ccccc5 | 124 | 10.1016/j.bmcl.2009.03.035 |
| 77 | c1cccc(c12)NC(=NS2(=O)=O)c(c3=O)c([O-])n(c(c34)cccc4)NCc5ccccc5 | 129 | 10.1016/j.bmcl.2005.01.071 |
| 78 | C1CCCCC[C@@H]([C@H]12)N(c(cc3)ccc3F)C(=O)[C@@H](C2=O)C(N4)=NS(=O)(=O)c(c45)cc(cc5)NS(=O)(=O)C | 130 | 10.1016/j.bmcl.2008.11.048 |
| 79 | O=C([O-])\C=C\c(cc1)ccc1NC(=O)[C@H](C)NC(=O)c(cc2)cc(c23)nc(-c4cocc4)n3C5CCCCC5 | 130 | 10.1016/j.bmcl.2009.10.136 |
| 80 | CC[C@H](C)[C@](CC(=O)[O-])(OCC1)C(=C12)N=C3[C@@H]2C(C#N)=CC=C3C | 130 | 10.1016/j.bmcl.2010.03.002 |
| 81 | C=1C=C(O)CC(C12)=C(C=N2)C[C@@H](c3cscn3)NC(=O)c(cc4)cc(c45)nc(-c6ccccn6)n5C7CCCCC7 | 140 | 10.1016/j.bmcl.2010.02.003 |
| 82 | N12CCN(C)S(=O)(=O)/N=C([O-])\c3ccc4c(c3)n(C)c(c4C5CCCCC5)c6c(cccc6)OCC(=O)N(CC1)CC2 | 140 | 10.1016/j.bmcl.2012.03.097 |
| 83 | [NH3+]CCOc(c1)ccc(c12)NC(=NS2(=O)=O)[C@H](C3=O)C(=O)N(CCC(C)C)c(c34)cccc4 | 142 | 10.1016/j.bmcl.2009.05.091 |
| 84 | CC(C)CC[C@@H](n(c12)ccc2)C([O-])=C(C1=O)C(=NS3(=O)=O)Nc(c34)ccc(c4)NS(=O)(=O)C | 150 | 10.1016/j.bmcl.2009.05.022 |
| 85 | c1cccc(c12)n(CCCC#N)c([O-])c(c2=O)C(=NS3(=O)=O)Nc(c34)cccc4 | 172 | 10.1021/jm050855s |
| 86 | C1CCCCC1n(c(c23)ccc(c2)C(=O)[O-])c(n3)-c4ccc(cc4)OCc5cnc(C)nc5-c6ccc(Cl)cc6 | 188 | 10.1021/jm060269e |
| 87 | C1C[C@H](C)CC[C@H]1C(=O)N(C(C)C)c2c(C(=O)[O-])cc(cc2)Oc3c(C(F)(F)F)cncc3 | 190 | 10.1016/j.bmcl.2013.09.102 |
| 88 | c1c(Br)c(Br)ccc1/C=C2\SC(=S)N(C2=O)NS(=O)(=O)c3ccccc3 | 200 | 10.1021/jm050859x |
| 89 | C1CCCCC1n(c(c23)ccc(c2)C(=O)[O-])c(n3)-c4ccc(cc4)OCc5cc(Cl)ccc5-c6ccccc6 | 200 | 10.1021/jm060269e |
| 90 | CC(C)(C)CCN([C@H](C1=O)C(C)(C)C)C(=O)[C@@H]1C2=[NH+]S(=O)(=O)c(c23)c(ccc3)OCC(=O)N | 200 | 10.1016/j.bmcl.2008.05.083 |
| 91 | n1nn[n-]c1-c(cn2)c(n23)ncc(c3C4CCCCC4)-c(cc5)ccc5OCc6cccc(c6)C(=O)OC | 220 | 10.1016/j.bmcl.2009.09.087 |
| 92 | O=C([O-])c(c1)ccc(c12)c(C3CCCCC3)c(C#C)n2CC(=O)N4CCC(CC4)[NH+](C)C | 228 | 10.1021/jm050056+ |
| 93 | s1cccc1-c(c2[O-])nn(CCC(C)C)c(=O)c2C(N3)=NS(=O)(=O)c(c34)cc(cc4)/C=C/C(=O)N | 230 | 10.1016/j.bmcl.2008.01.007 |
| 94 | c1cc(C)ccc1C(=O)N/C(C(=O)[O-])=C\c2ccc(cc2)Oc3c(Br)cccc3 | 250 | 10.1016/j.bmcl.2005.03.106 |
| 95 | O=C([O-])[C@@H]1CCCN1S(=O)(=O)c2c([O-])c(Br)cc(Cl)c2 | 260 | 10.1021/jm060168g |
| 96 | FC(F)(F)c1ccccc1Oc(cc2)cc(C(=O)[O-])c2NS(=O)(=O)c(cc3)ccc3C | 270 | 10.1016/j.bmcl.2013.09.102 |
| 97 | O=S(=O)(C)Nc(c1)ccc(c12)NC(=NS2(=O)=O)C(C3=O)=C([O-])[C@](C)(c(c34)cccc4)CCCc5ccccc5 | 280 | 10.1016/j.bmcl.2004.12.030 |
| 98 | O=C([O-])c(c1)ccc(c12)c(C3CCCCC3)c(CC)n2CC(=O)N4CCC(CC4)[NH+](C)C | 283 | 10.1021/jm050056+ |
| 99 | C=1C=C(O)CC(C12)=C(C=N2)C[C@@H](c3cscn3)NC(=O)c(cc4)cc(c45)nc(-c6cocc6)n5C7CCCCC7 | 300 | 10.1016/j.bmcl.2009.10.136 |
| 100 | Brc1cc(C(F)(F)F)cc(c1)Nc2c(C#N)c([O-])ns2 | 300 | 10.1016/j.bmcl.2006.10.002 |
| 101 | C1C[C@H](C)CC[C@H]1C(=O)N(C(C)C)c2c(C(=O)[O-])cc(cc2)Oc3ccccc3C(F)(F)C | 300 | 10.1016/j.bmcl.2013.09.102 |
| 102 | Fc1cc(F)cc(F)c1Cn2cnc(=O)c(c23)cc(cc3)Oc4ccccc4 | 300 | 10.1016/j.bmcl.2013.05.037 |
| 103 | c1ccncc1Cn(c(c23)cccc2)c([O-])c(c3=O)C(=NS4(=O)=O)Nc(c45)cccc5 | 316 | 10.1021/jm050855s |
| 104 | N#Cc1ccc(C)c(c12)sc3c2CCO[C@]3(CC([O-])=O)CCC | 320 | 10.1016/j.bmcl.2005.08.114 |
| 105 | C1CCCCC1c(c(c23)ccc(n2)C([O-])=O)c(n3CC(=O)N(C)C)-c4ccccc4 | 320 | 10.1016/j.bmcl.2006.05.012 |
| 106 | s1cccc1-c(c2[O-])nn(CCC(C)C)c(=O)c2C(N3)=NS(=O)(=O)c(c34)cc(O)cc4 | 340 | 10.1016/j.bmcl.2008.01.007 |
| 107 | c1ccccc1Oc(cc2)cc(C(=O)[O-])c2NS(=O)(=O)c(cc3)c(F)cc3Br | 340 | 10.1016/j.bmcl.2013.09.102 |
| 108 | c1ccccc1CN([C@H](C2=O)C(C)C)C(=O)C2=C3NS(=O)(=O)c(c34)c(ccc4)OCc5nocn5 | 350 | 10.1016/j.bmcl.2008.05.083 |
| 109 | CCC[C@@](CC(=O)[O-])(OCC1)C(=C12)N=C3[C@@H]2C(Cl)=CC=C3Cl | 370 | 10.1016/j.bmcl.2010.03.002 |
| 110 | C1=CC(O)=C[C@@H](C12)C(=CN=2)C[C@@H](C([O-])=O)NC(=O)c(cc3)cc(c34)nc(n4C5CCCCC5)C(=O)c(cc6)cc(c67)nc(-c8cocc8)n7C9CCCC9 | 380 | 10.1016/j.bmcl.2010.02.003 |
| 111 | c1cocc1-c(n2)n(C3CCCCC3)c(c24)ccc(c4)C(=O)N[C@H](C(=O)[O-])Cc5c[nH]c(c56)cccc6 | 400 | 10.1016/j.bmcl.2003.12.032 |
| 112 | n1nn[n-]c1-c(cn2)c(n23)ncc(c3C4CCCCC4)-c(cc5)ccc5OC6CCCCC6 | 400 | 10.1016/j.bmcl.2009.09.087 |
| 113 | s1cccc1-c2nn(CCC(C)(C)CC)c(=O)c(c2[O-])C(N3)=CS(=O)(=O)c(c34)cc(cc4)[N-]S(=O)(=O)C | <10 | 10.1016/j.bmcl.2008.07.014 |
| 114 | c1ccn(c12)n(CCC(C)(C)C)c([O-])c(c2=O)C(N3)=CS(=O)(=O)c(c34)cc(cc4)[N-]S(=O)(=O)C | <10 | 10.1016/j.bmcl.2008.07.014 |
| 115 | c1cc(F)ccc1CN([C@@H]([C@@H]23)CCC3)C(=O)[C@@H](C2=O)C(N4)=NS(=O)(=O)c(c45)cc(cc5)NS(=O)(=O)C | <10 | 10.1016/j.bmcl.2008.11.048 |
| 116 | C1CC[C@@H]([C@@H]12)N(CCC(C)C)C(=O)[C@@H](C2=O)C(N3)=NS(=O)(=O)c(c34)cc(cc4)NS(=O)(=O)C | <10 | 10.1016/j.bmcl.2008.11.048 |
