# Supplementary material for: Discovery of Novel Hepatitis C Virus NS5B Polymerase Inhibitors by Combining Random Forest, Multiple e-Pharmacophore Modeling and Docking
Source: PLoS One. 2016 Feb 4;11(2):e0148181. doi: 10.1371/journal.pone.0148181 (PMC4742222; doi:10.1371/journal.pone.0148181)
Supplement: S4 Table — (DOC) [file pone.0148181.s009.doc]

**S4 Table. Important descriptors used in the RF model and their importance values.**

| descriptor | | | |  |
| --- | --- | --- | --- | --- |
| no. | name | class | definition | importance values |
| 1 | RDF090m | RDF descriptors | Radial Distribution Function - 090 / weighted by mass | 25.44 |
| 2 | SM12_AEA(dm) | Edge adjacency indices | spectral moment of order 12 from augmented edge adjacency mat. weighted by dipole moment | 25.36 |
| 3 | RTs | GETAWAY descriptors | R total index / weighted by I-state | 22.88 |
| 4 | SM02_AEA(ri) | Edge adjacency indices | spectral moment of order 2 from augmented edge adjacency mat. weighted by resonance integral | 22.68 |
| 5 | SM09_EA(bo) | Edge adjacency indices | spectral moment of order 9 from edge adjacency mat. weighted by bond order | 22.23 |
| 6 | SsssCH | Atom-type E-state indices | Sum of sssCH E-states | 21.93 |
| 7 | SM04_AEA(ri) | Edge adjacency indices | spectral moment of order 4 from augmented edge adjacency mat. weighted by resonance integral | 21.75 |
| 8 | SM14_AEA(dm) | Edge adjacency indices | spectral moment of order 14 from augmented edge adjacency mat. weighted by dipole moment | 21.67 |
| 9 | GGI4 | 2D autocorrelations | topological charge index of order 4 | 20.45 |
| 10 | HTs | GETAWAY descriptors | H total index / weighted by I-state | 19.9 |
| 11 | CATS2D_04_LL | CATS 2D | CATS2D Lipophilic-Lipophilic at lag 04 | 19.83 |
| 12 | SM12_EA(ri) | Edge adjacency indices | spectral moment of order 12 from edge adjacency mat. weighted by resonance integral | 18.78 |
| 13 | CATS2D_03_LL | CATS 2D | CATS2D Lipophilic-Lipophilic at lag 03 | 17.93 |
| 14 | MPC08 | Walk and path counts | molecular path count of order 8 | 17.43 |
| 15 | H2s | GETAWAY descriptors | H autocorrelation of lag 2 / weighted by I-state | 15.76 |
| 16 | Ram | Topological indices | ramification index | 13.22 |
