# Supplementary material for: Discovery of Novel Hepatitis C Virus NS5B Polymerase Inhibitors by Combining Random Forest, Multiple e-Pharmacophore Modeling and Docking
Source: PLoS One. 2016 Feb 4;11(2):e0148181. doi: 10.1371/journal.pone.0148181 (PMC4742222; doi:10.1371/journal.pone.0148181)
Supplement: S5 Table — (DOC) [file pone.0148181.s010.doc]

**S5 Table. Results of RF model validation by three datasets.**

| dataset | model | no. of descriptors | TP*^d^* | FN*^e^* | TN*^f^* | FP*^g^* | SE (%)*^h^* | SP (%)*^i^* | Q (%)*^j^* |
| --- | --- | --- | --- | --- | --- | --- | --- | --- | --- |
| Set-150*^a^* | I | 626 | 60 | 14 | 57 | 10 | 81.1 | 85.1 | 83.0 |
|  | II | 44 | 58 | 16 | 60 | 7 | 78.4 | 89.6 | 83.7 |
|  | III | 13 | 55 | 19 | 58 | 9 | 74.3 | 86.6 | 80.1 |
| Set-400*^b^* | I | 577 | 57 | 17 | 56 | 11 | 77.0 | 83.6 | 80.1 |
|  | II | 43 | 58 | 16 | 56 | 11 | 78.4 | 83.6 | 80.9 |
|  | III | 16 | 60 | 14 | 59 | 8 | 81.1 | 88.1 | 84.4 |
| Set-950*^c^* | I | 626 | 63 | 11 | 60 | 7 | 85.1 | 89.6 | 87.2 |
|  | II | 47 | 59 | 15 | 61 | 6 | 79.7 | 91.0 | 85.1 |
|  | III | 12 | 55 | 19 | 59 | 8 | 74.3 | 88.1 | 80.9 |

*^a^*Set-150: positive ≤150nm, negative > 150nm. *^b^*Set-400: positive≤400nm, negative≥600nm. *^c^*Set-950: positive ≤950nm, negative > 950nm. *^d^* TP, true positive. *^e^*FN, false negative. *^f^*TN, true negative. *^g^*FP, false positive. *^h^*SE(%): sensitivity, SE = TP/(TP+FN). *^i^*SP(%): specificity, SP = TN/(TN+FP). *^j^*Q(%): overall accuracy, Q=(TP+TN)/(TP+FP+TN+FN).

**Detailed discussion of S5 Table.** To evaluate the effect of various activity samples on random forest accuracy and performance, we construct three datasets: (i) Set-150: positives ≤ 150 nM, negatives > 150 nM, (ii) Set-400: positives ≤ 400 nM, negatives ≥ 600 nM, (iii) Set-950: positives ≤ 950 nM, negatives > 950 nM [1]. And three models are built for each dataset based on the accuracy decrement importance of descriptor: (i) model I: accuracy decrement importance ≥ 0, (ii) model II: accuracy decrement importance ≥ 5.0, (iii) model III: accuracy decrement importance ≥ 6.0. And the results of RF model validation by above three datasets are shown in Table S5. For Set-150, the model III has a value of SE, SP, and Q were 74.3%, 86.6%, and 80.1%, respectively, with 13 descriptors. For Set-950, the model III has a value of SE, SP, and Q were 74.3%, 88.1%, and 80.9%, respectively, with 12 descriptors. For Set-400, the model III has a good SE, SP, and Q values were 81.1%, 88.1% and 84.4%, respectively, with 16 descriptors. Therefore, we can see that Set-400 generated better RF model than Set-150 and Set-950. So, Set-400 in our RF model is reasonable.

**Reference**

1. Weidlich IE, Filippov I V, Brown J, Kaushik-Basu N, Krishnan R, Nicklaus MC, et al. Inhibitors for the hepatitis C virus RNA polymerase explored by SAR with advanced machine learning methods. Bioorg Med Chem. Elsevier Ltd; 2013;21: 3127–37. doi: 10.1016/j.bmc.2013.03.032 PMID: 23608107
