# Supplementary material for: Discovery of Novel Hepatitis C Virus NS5B Polymerase Inhibitors by Combining Random Forest, Multiple e-Pharmacophore Modeling and Docking
Source: PLoS One. 2016 Feb 4;11(2):e0148181. doi: 10.1371/journal.pone.0148181 (PMC4742222; doi:10.1371/journal.pone.0148181)
Supplement: S6 Table — (DOC) [file pone.0148181.s011.doc]

**S6 Table. Results of the chosen randomly data set RF model and chosen by scaffolds RF model validation by independent test sets.**

| method | model | no. of descriptors | TP*^a^* | FN*^b^* | TN*^c^* | FP*^d^* | SE (%)*^e^* | SP (%)*^f^* | Q (%)*^g^* |
| --- | --- | --- | --- | --- | --- | --- | --- | --- | --- |
| scaffold | I | 577 | 57 | 17 | 56 | 11 | 77.0 | 83.6 | 80.1 |
|  | II | 43 | 58 | 16 | 56 | 11 | 78.4 | 83.6 | 80.9 |
|  | III | 16 | 60 | 14 | 59 | 8 | 81.1 | 88.1 | 84.4 |
| random | I | 627 | 60 | 14 | 56 | 11 | 81.1 | 83.6 | 82.3 |
|  | II | 35 | 61 | 13 | 55 | 12 | 82.4 | 82.1 | 82.3 |
|  | III | 14 | 58 | 16 | 55 | 12 | 78.4 | 82.1 | 80.1 |

*^a^* TP, true positive. *^b^*FN, false negative. *^c^*TN, true negative. *^d^*FP, false positive. *^e^*SE(%): sensitivity, SE = TP/(TP+FN). *^f^*SP(%): specificity, SP = TN/(TN+FP). *^g^*Q(%): overall accuracy, Q=(TP+TN)/(TP+FP+TN+FN).

**Detailed discussion of S6 Table.** To ensure the data sets having a relatively equal distribution in the chemical structural space, we construct the training set according to compound distributions in the chemical space based on their scaffolds. There are several papers also chose compounds from each category based on scaffolds to create the training and test set [1,2]. Martin et al also indicated that rational division method usually yielded better results than random division [3]. In this study, we evaluated the performance of two methods (random and scaffold) by independent test sets and determined whether scaffold method lead to more predictive model compared to random method. The results are shown in Table S6. For scaffold method, the model III has a value of SE, SP, and Q were 81.1%, 88.1%, and 84.4%, respectively, with 16 descriptors. For random method, the model III has a value of SE, SP, and Q were 78.4%, 82.1%, and 80.1%, respectively, with 14 descriptors. The results showed that models based on scaffold method generated better statistical results for the test sets than models based on random division.

**Reference**

1. Ren JX, Li LL, Zheng RL, Xie HZ, Cao ZX, Feng S, et al. Discovery of novel Pim-1 kinase inhibitors by a hierarchical multistage virtual screening approach based on svm model, pharmacophore, and molecular docking. J Chem Inf Model. 2011;51: 1364–1375. doi: 10.1021/ci100464b PMID: 21618971

2. Wei L, Ying X. Prediction of Hepatitis C Virus Non-Structural Proteins 5B Polymerase Inhibitors Using Machine Learning Methods. Acta Phys -Chim Sin. 2011;27: 1407–1416.

3. Martin TM, Harten P, Young DM, Muratov EN, Golbraikh A, Zhu H, et al. Does rational selection of training and test sets improve the outcome of QSAR modeling? J Chem Inf Model. 2012;52: 2570–2578. doi: 10.1021/ci300338w PMID: 23030316
