# Supplementary material for: Discovery of Novel Hepatitis C Virus NS5B Polymerase Inhibitors by Combining Random Forest, Multiple e-Pharmacophore Modeling and Docking
Source: PLoS One. 2016 Feb 4;11(2):e0148181. doi: 10.1371/journal.pone.0148181 (PMC4742222; doi:10.1371/journal.pone.0148181)
Supplement: S7 Table — (DOC) [file pone.0148181.s012.doc]

**S7 Table. Validation of e-pharmacophore 3HHK models.**

| Hypothesis | EF1%*^a^* | RIE*^b^* | BEDROC(α=160.9)*^c^* | BEDROC(α=20) |
| --- | --- | --- | --- | --- |
| A5A6D8D10 | 1.5 | 0.62 | 0.141 | 0.053 |
| A5A6D8R14 | 12 | 3.09 | 0.733 | 0.263 |
| A5A6D8R16 | 4.6 | 1.99 | 0.345 | 0.169 |
| A5A6D10R16 | 4.6 | 2.00 | 0.232 | 0.171 |
| A5A6D10R14 | 3.1 | 2.60 | 0.256 | 0.222 |
| A5A6R14R16 | 12 | 6.02 | 0.772 | 0.514 |
| A5D8D10R14 | 1.5 | 0.57 | 0.066 | 0.048 |
| A5D8D10R16 | 0 | 0.18 | 0.001 | 0.015 |
| A5D8R14R16 | 7.7 | 1.93 | 0.452 | 0.165 |
| A5D10R14R16 | 4.6 | 1.13 | 0.260 | 0.096 |
| A6D8D10R14 | 1.5 | 0.31 | 0.140 | 0.027 |
| A6D8R14R16 | 4.6 | 1.54 | 0.365 | 0.131 |
| A6D10R14R16 | 4.6 | 1.28 | 0.272 | 0.109 |

*^a^*EF: Enrichment factor at 1% of the decoy data set. *^b^*RIE: Robust initial enhancement. *^c^*BEDROC: Boltzmann-enhanced discrimination of receiver operating characteristic.
