# Supplementary material for: Discovery of Novel Hepatitis C Virus NS5B Polymerase Inhibitors by Combining Random Forest, Multiple e-Pharmacophore Modeling and Docking
Source: PLoS One. 2016 Feb 4;11(2):e0148181. doi: 10.1371/journal.pone.0148181 (PMC4742222; doi:10.1371/journal.pone.0148181)
Supplement: S8 Table — (DOC) [file pone.0148181.s013.doc]

**S8 Table. Validation of e-pharmacophore 3SKA models.**

| Hypothesis | EF1%*^a^* | RIE*^b^* | BEDROC(α=160.9)*^c^* | BEDROC(α=20) |
| --- | --- | --- | --- | --- |
| A2D3R9R10R11 | 9.2 | 1.88 | 0.527 | 0.161 |
| D5H6R9R10R11 | 1.5 | 1.78 | 0.038 | 0.152 |
| D3H6R9R10R11 | 4.6 | 1.13 | 0.298 | 0.096 |
| D3D5R9R10R11 | 3.1 | 0.62 | 0.261 | 0.053 |
| D3D5H6R10R11 | 3.1 | 0.59 | 0.166 | 0.050 |
| D3D5H6R9R11 | 3.1 | 0.57 | 0.125 | 0.048 |
| D3D5H6R9R10 | 1.5 | 0.49 | 0.045 | 0.042 |
| A2H6R9R10R11 | 4.6 | 1.17 | 0.301 | 0.100 |
| A2D5R9R10R11 | 3.1 | 0.88 | 0.261 | 0.075 |
| A2D5H6R10R11 | 0 | 0.93 | 0.026 | 0.079 |
| A2D5H6R9R11 | 4.6 | 3.71 | 0.238 | 0.317 |
| A2D5H6R9R10 | 0 | 0.76 | 0.001 | 0.065 |
| A2D3H6R10R11 | 6 | 1.59 | 0.281 | 0.136 |
| A2D3H6R9R11 | 3.1 | 1.11 | 0.245 | 0.095 |
| A2D3H6R9R10 | 4.6 | 1.13 | 0.298 | 0.096 |
| A2D3D5R10R11 | 3.1 | 0.62 | 0.261 | 0.053 |
| A2D3D5R9R10 | 3.1 | 0.75 | 0.193 | 0.064 |
| A2D3D5R9R11 | 3.1 | 0.75 | 0.261 | 0.064 |
| A2D3D5H6R11 | 3.1 | 0.61 | 0.210 | 0.052 |
| A2D3D5H6R10 | 3.1 | 0.58 | 0.143 | 0.049 |
| A2D3D5H6R9 | 0 | 1.11 | 0.064 | 0.095 |

*^a^*EF: Enrichment factor at 1% of the decoy data set. *^b^*RIE: Robust initial enhancement. *^c^*BEDROC: Boltzmann-enhanced discrimination of receiver operating characteristic.
