# Supplementary material for: Discovery of Novel Hepatitis C Virus NS5B Polymerase Inhibitors by Combining Random Forest, Multiple e-Pharmacophore Modeling and Docking
Source: PLoS One. 2016 Feb 4;11(2):e0148181. doi: 10.1371/journal.pone.0148181 (PMC4742222; doi:10.1371/journal.pone.0148181)
Supplement: S9 Table — (DOC) [file pone.0148181.s014.doc]

**S9 Table. Validation of e-pharmacophore 4DRU models.**

| Hypothesis | EF1%*^a^* | RIE*^b^* | BEDROC(α=160.9)*^c^* | BEDROC(α=20) |
| --- | --- | --- | --- | --- |
| A5H8H10R13 | 0 | 1.39 | 0.010 | 0.096 |
| A5H8H10R12 | 0 | 0.42 | 0 | 0.029 |
| A5H8R12R13 | 29 | 10.83 | 0.969 | 0.751 |
| A5H10R12R13 | 0 | 0.80 | 0.002 | 0.055 |
| H8H10R12R13 | 0 | 1.48 | 0.005 | 0.102 |

*^a^*EF: Enrichment factor at 1% of the decoy data set. *^b^*RIE: Robust initial enhancement. *^c^*BEDROC: Boltzmann-enhanced discrimination of receiver operating characteristic.
