# Supplementary material for: Discovery of Novel Hepatitis C Virus NS5B Polymerase Inhibitors by Combining Random Forest, Multiple e-Pharmacophore Modeling and Docking
Source: PLoS One. 2016 Feb 4;11(2):e0148181. doi: 10.1371/journal.pone.0148181 (PMC4742222; doi:10.1371/journal.pone.0148181)
Supplement: S10 Table — (DOC) [file pone.0148181.s015.doc]

**S10 Table. Validation of e-pharmacophore 2GIR models.**

| Hypothesis | EF1%*^a^* | RIE*^b^* | BEDROC(α=160.9)*^c^* | BEDROC(α=20) |
| --- | --- | --- | --- | --- |
| N5H2R7 | 12 | 4.20 | 0.292 | 0.247 |
| N5H4R7 | 0 | 3.18 | 0.010 | 0.187 |
| H2H4R7 | 0 | 1.89 | 0.013 | 0.111 |

*^a^*EF: Enrichment factor at 1% of the decoy data set. *^b^*RIE: Robust initial enhancement. *^c^*BEDROC: Boltzmann-enhanced discrimination of receiver operating characteristic.
