# Supplementary material for: Discovery of Novel Hepatitis C Virus NS5B Polymerase Inhibitors by Combining Random Forest, Multiple e-Pharmacophore Modeling and Docking
Source: PLoS One. 2016 Feb 4;11(2):e0148181. doi: 10.1371/journal.pone.0148181 (PMC4742222; doi:10.1371/journal.pone.0148181)
Supplement: S11 Table — (DOC) [file pone.0148181.s016.doc]

**S11 Table. Validation of e-pharmacophore 3PHE models.**

| Hypothesis | EF1%*^a^* | RIE*^b^* | BEDROC(α=160.9)*^c^* | BEDROC(α=20) |
| --- | --- | --- | --- | --- |
| A4R11R13R14 | 12 | 2.34 | 0.228 | 0.138 |
| A4H7R11R13 | 6 | 1.51 | 0.098 | 0.089 |
| A4H7R11R14 | 6 | 2.14 | 0.138 | 0.126 |
| H7R11R13R14 | 6 | 2.27 | 0.138 | 0.134 |
| A4H7R13R14 | 6 | 1.85 | 0.100 | 0.109 |

*^a^*EF: Enrichment factor at 1% of the decoy data set. *^b^*RIE: Robust initial enhancement. *^c^*BEDROC: Boltzmann-enhanced discrimination of receiver operating characteristic.
