# Supplementary material for: Discovery of Novel Hepatitis C Virus NS5B Polymerase Inhibitors by Combining Random Forest, Multiple e-Pharmacophore Modeling and Docking
Source: PLoS One. 2016 Feb 4;11(2):e0148181. doi: 10.1371/journal.pone.0148181 (PMC4742222; doi:10.1371/journal.pone.0148181)
Supplement: S12 Table — (DOC) [file pone.0148181.s017.doc]

**S12 Table. ROC AUCs and enrichment factors (EF) obtained for the set-noW, set-2W and set-3W proteins.**

| Ranking efficiency | 3HHK-noW*^a^* | 3SKA-noW | 3HHK-2W*^b^* | 3SKA-2W | 3HHK-3W*^c^* | 3SKA-3W |
| --- | --- | --- | --- | --- | --- | --- |
| AUC | 0.81 | 0.58 | 0.72 | 0.66 | 0.76 | 0.72 |
| EF2% | 0.13 | 0.07 | 0.08 | 0.13 | 0.11 | 0.16 |
| EF3% | 0.18 | 0.07 | 0.08 | 0.15 | 0.14 | 0.18 |
| EF4% | 0.21 | 0.10 | 0.13 | 0.18 | 0.14 | 0.18 |

*^a^*noW: Absence of any water molecules. *^b^*2W: The two main waters (i.e., WB and W2) mediate the interactions of various ligands with the protein, as in Barreca *et al* (2013). *^c^*3W: The three main waters (i.e., WB, W2 and W5) interact with both the protein and ligands, as in Barreca *et al* (2013).

**Detailed discussion of S12 Table.** In the paper of Barreca [1], cross-docking experiments indicated that the presence of explicit water molecules always improved the docking performance of WMIs (water-mediated inhibitors). And the formation of water-mediated hydrogen bond interaction provided higher docking scores for many of the ligands. At the same time, they pointed out that the impact of water molecules in docking-based experiments was highly dependent on several factors like target identity and conformation and ligand chemotype. When dealing with NWMIs (nonwater-mediated inhibitors), the water molecules probably imposed steric constraints that hamper the ligand to correctly fit the allosteric site. As evident from bound crystal structures, most of the NWMIs are able to displace WB (water bridge) by placing an acceptor group in the position usually occupied by the WB oxygen.

In this study, to evaluate the effect of water molecule on docking-based virtual screening simulations, 63 inhibitors and 1000 decoys molecules were docked against two NS5B polymerase crystal structures 3HHK and 3SKA that represent bound inhibitors in the palm I region. The results show that the best performing structures were found to be 3HHK-noW (3HHK with no water molecules) and 3SKA-3W (3SKA with water WB, W2 and W5), with ROC AUC of 0.81 and 0.72 and enrichment factors of 0.21 and 0.18 at 4% of the virtual screening, respectively (see Table S12). However, for 3HHK, the presence of water molecules (3HHK-2W and 3HHK-3W) reduce the accuracy of virtual screening with the ROC AUC of 0.72 and 0.76 and enrichment factors of 0.13 and 0.14 at 4% of the virtual screening, respectively. For 3SKA, although the presence of water molecules improve the accuracy of the virtual screening, water molecules can be replaced by an acceptor group as described by Barreca. Then we removed water molecules from the complexes in docking procedure.

**Reference**

1. Barreca ML, Iraci N, Manfroni G, Gaetani R, Guercini C, Sabatini S, et al. Accounting for target flexibility and water molecules by docking to ensembles of target structures: The HCV NS5B palm site i inhibitors case study. J Chem Inf Model. 2014;54: 481–497. doi: 10.1021/ci400367m PMID: 23952658
