# Supplementary material for: Discovery of Novel Hepatitis C Virus NS5B Polymerase Inhibitors by Combining Random Forest, Multiple e-Pharmacophore Modeling and Docking
Source: PLoS One. 2016 Feb 4;11(2):e0148181. doi: 10.1371/journal.pone.0148181 (PMC4742222; doi:10.1371/journal.pone.0148181)
Supplement: S13 Table — (DOC) [file pone.0148181.s018.doc]

**S13 Table. Evaluation results of the performance of various VS methods by screening a validation set that comprises 73 known HCV NS5B inhibitors and 2190 decoys from pubchem database.**

| Method | Predicted inhibitors | Hits | Yield (%)*^a^* | Hit rate (%)*^b^* | Enrichment factor*^c^* | Time cost (hours) |
| --- | --- | --- | --- | --- | --- | --- |
| RB-VS | 361 | 68 | 93.15 | 15.85 | 5.84 | 0.071 |
| PB-VS | 432 | 64 | 87.67 | 14.81 | 4.59 | 2.167 |
| DB-VS | 592 | 65 | 89.04 | 8.89 | 3.82 | 22.044 |
| RB/PB | 91(361/91) | 56(68/56) | 76.71 | 61.54 | 19.08 | 0.353 |
| RB/PB/DB | 67(361/91/67) | 56(68/56/51) | 69.86 | 76.12 | 25.96 | 1.160 |

*^a^*Yield: percentage of predicted compounds in known inhibitors. *^b^*Hit rate: percentage of known inhibitors in predicted compounds. *^c^*Enrichment factor: ratio of hit rate to the percentage of known inhibitors in validation set.

**Detailed discussion of S13 Table.** In order to evaluate the performance of the multistage VS approach, we created a validation set that comprises 73 known HCV NS5B polymerase inhibitors and 2190 decoys from PubChem database to assess different VS methods. Results were presented in Table S13. First, the RB-VS, PB-VS and DB-VS methods were individually applied to screen the validation set. For the RB-VS, 68 of 73 positives were predicted correctly and 1897 of 2190 negatives were correctly predicted with a positive yield of 93.15%, a hit rate of 15.85%, and an enrichment factor of 5.84. The time cost in the screening of the validation set by the RB-VS is approximately 0.071 hours on 1 processor, which includes the time spent for the calculation of molecular descriptors. For the PB-VS method, 64 of 73 positives were predicted correctly and 1822 of 2190 negatives were correctly predicted, with a positive yield 87.67%, a hit rate of 14.81%, and an enrichment factor of 4.59. The time cost in the screening of the validation set by PB-VS is approximately 2.167 hours. For the DB-VS method, 65 of 73 positives were predicted correctly and 1663 of 2190 negatives were correctly predicted, with a positive yield 89.04%, a hit rate of 8.89%, and an enrichment factor of 3.82. The time cost in the screening of the validation set by PB-VS is approximately 22.044 hours. All the tests were run on the same computer. Therefore, the RB-VS was applied as the first filter as it was shown to be the fastest among the selected VS methods (see Table S13). DB-VS was the last filter applied because it was the slowest.

Further, the RB-VS and PB-VS were combined to screen the validation set with RB-VS first performed followed by PB-VS. The RB-VS identified 68 positive compounds. They were further filtered by PB-VS, and 56 of 68 positives were predicted correctly, with a positive yield 76.71%, a hit rate of 61.54%, and an enrichment factor of 19.08. The time cost in the screening of the validation set by RB/PB VS is approximately 0.353 hours. Obviously, the combination of RB-VS and PB-VS considerably increases the hit rate and the enrichment factor compared with the sole use of RB-VS or PB-VS, and the time used is also reduced compared with the sole use of PB-VS.

Finally the Combining RB-VS, PB-VS and DB-VS method was employed to screen the validation set with RB-VS performed first, followed by PB-VS, and finally DB-VS. As mentioned above, the combined RB/PB VS method predicted 56 positive compounds. They were further screened by DB-VS, and 51 of 56 positives were predicted correctly, with a positive yield 69.86%, a hit rate of 76.12%, and an enrichment factor of 25.96. The time cost in the screening of the validation set by RB/PB/DB VS is approximately 1.160 hours. Results presented above, the combination of RB-VS, PB-VS and DB-VS significantly increases the hit rate and the enrichment factor compared with the sole use of RB-VS, PB-VS, and DB-VS as well as the combined RB/PB VS. And the time used is also reduced compared with the sole use of DB-VS. Hence, the combined VS methods is good alternatives for single VS methods and are suitable for screening large databases such as the NCI database. And the RB/PB/DB sequence for these three methods is the best one.
