# Supplementary material for: Discovery of Novel Hepatitis C Virus NS5B Polymerase Inhibitors by Combining Random Forest, Multiple e-Pharmacophore Modeling and Docking
Source: PLoS One. 2016 Feb 4;11(2):e0148181. doi: 10.1371/journal.pone.0148181 (PMC4742222; doi:10.1371/journal.pone.0148181)
Supplement: S14 Table — (DOC) [file pone.0148181.s019.doc]

**S14 Table.** The number of compounds from NCI database and time consumption after glide SP docking, e-pharmacophore, random forest, multistage virtual screening and data fusion methods.

| PDB | Docking-score (SP) | Number of compounds  (DB) | Fitness  (PB) | Number of compounds (PB) |
| --- | --- | --- | --- | --- |
| 3HHK | -6.0 | 43,028 | 1.6 | 2835 |
| 3SKA | -6.0 | 73,110 | 1.5 | 452 |
| 2BRK | -6.0 | 48,338 | 2.0 | 1 |
| 4DRU | -6.0 | 124,047 | 1.8 | 141 |
| 2GIR | -6.0 | 42,262 | 1.8 | 1619 |
| 3PHE | -6.0 | 23,172 | 1.8 | 246 |
| Method | | Total number of compounds | Time (hours) | |
| Random Forest (RB) | | 14,895 | ~8 | |
| E-pharmacophore (PB) | | 3569 | ~72 | |
| Glide SP Docking (DB) | | 148,337 | ~7911 | |
| Multistage VS (RB-PB-DB) | | 539 | ~20 | |
| Data Fusion (RB+PB+DB) | | 1070 | ~7960 | |
