# Supplementary material for: Discovery of Novel Hepatitis C Virus NS5B Polymerase Inhibitors by Combining Random Forest, Multiple e-Pharmacophore Modeling and Docking
Source: PLoS One. 2016 Feb 4;11(2):e0148181. doi: 10.1371/journal.pone.0148181 (PMC4742222; doi:10.1371/journal.pone.0148181)
Supplement: S15 Table — (DOC) [file pone.0148181.s020.doc]

**S15 Table.** Values of pK_D_, pIC_50_ and logP calculated using the program QikProp for compounds N1-N5.

| Compounds | pK_D_ | pIC_50_ | QPlogPo/w |
| --- | --- | --- | --- |
| N1 | 4.20 | 5.44 | 3.48 |
| N2 | 5.33 | 5.52 | 5.24 |
| N3 | 4.06 | 4.62 | 1.41 |
| N4 | 3.91 | 5.70 | 4.25 |
| N5 | -- | 5.66 | 4.19 |
