# Supplementary material for: Discovery of Novel Hepatitis C Virus NS5B Polymerase Inhibitors by Combining Random Forest, Multiple e-Pharmacophore Modeling and Docking
Source: PLoS One. 2016 Feb 4;11(2):e0148181. doi: 10.1371/journal.pone.0148181 (PMC4742222; doi:10.1371/journal.pone.0148181)
Supplement: S16 Table — (DOC) [file pone.0148181.s021.doc]

**S16 Table. Compare the Obtained Results with Different Papers [1–3].**

| Paper (DOI) | Method | | Activity | |
| --- | --- | --- | --- | --- |
| DOI:10.1021/ci9004749 | | 3D-QSAR and structure based screen | | IC_50_: 0.01~20 µM |
| DOI:10.1016/j.ejmech.2014.01.062 | | Chemical genetics-based discovery | | EC_50_: 1.1~100 µM |
| DOI:10.1021/jm4004522 | | MD Simulations and Structure-Based Design | | EC_50_: 3.3~380 nM |
| This paper | | Combining RB-PB-DB | | EC_50_: 1.61~26.88 µM  IC_50_: 2.01~3.63 µM |

**Reference**

1. Musmuca I, Caroli A, Mai A, Kaushik-Basu N, Arora P, Ragno R. Combining 3-D quantitative structure-activity relationship with ligand based and structure based alignment procedures for in silico screening of new hepatitis c virus NS5B polymerase inhibitors. J Chem Inf Model. 2010;50: 662–676. doi: 10.1021/ci9004749 PMID: 20225870

2. Jin G, Lee S, Choi M, Son S, Kim GW, Oh JW, et al. Chemical genetics-based discovery of indole derivatives as HCV NS5B polymerase inhibitors. Eur J Med Chem. Elsevier Masson SAS; 2014;75: 413–425. doi: 10.1016/j.ejmech.2014.01.062 PMID: 24561671

3. Hucke O, Coulombe R, Bonneau P, Bertrand-Laperle M, Brochu C, Gillard J, et al. Molecular dynamics simulations and structure-based rational design lead to allosteric HCV NS5B polymerase thumb pocket 2 inhibitor with picomolar cellular replicon potency. J Med Chem. 2014;57: 1932–1943. doi: 10.1021/jm4004522 PMID: 23773186
