# Supplementary material for: Discovery of Novel Hepatitis C Virus NS5B Polymerase Inhibitors by Combining Random Forest, Multiple e-Pharmacophore Modeling and Docking
Source: PLoS One. 2016 Feb 4;11(2):e0148181. doi: 10.1371/journal.pone.0148181 (PMC4742222; doi:10.1371/journal.pone.0148181)
Supplement: S1 File — (ZIP) [file pone.0148181.s005.zip › N1 Final Report.doc]

PracticaChem

**Project Report**

Code:

Project Leader: Li Wang

Project member: Jing Wang

Jul , 9 , 2014

# 1. Delivery Information

| Compound ID | Struture | Amount | Spectra | Delivery Date |
| --- | --- | --- | --- | --- |
| N1 |  | 55mg | PN310-34 | 7,9,2014 |

# 2. Goal

To synthesize 55mg of compound

**Synthetic route**

# 3. Experimental and discussion

**3**

**Experimental:** **PN310-27**

Benzenesulfonyl chloride（1g，5.66mmol）was added dropwised to a solution of tert-butyl hydrazinecarboxylate（1.1g，8.5 mmol）in ether（20ml）at 0℃, then the reaction solution was stirred at room temperature for 1hr. Monitored by TLC, purified by Pre-TLC to get 850mg product of white solid, yield55.1%.

**4**

**Experimental: PN310-33**

Compound **3**（350mg，1.3mmol）dissolved in DCM（2.5ml），TFA（2.5ml）was added, then the reaction was stirred at room temperature for 3h. Monitored by TLC, the reaction mixture was concentrated , the residue was dissolved in EA, washed with 1M HCl for 3 times，combined the aqueous，adjust pH 8~9， extracted with EA for 3times，combined the organic layer，washed with brine, dried with Na2SO4 and concentrated to give compound **4** 140mg, yield 62.5%.

**Note:**

**7**

**Experimental:** **PN310-13**

A solution of Compound **5**（5g，5.4mmol）and triethoxymethane（4.47ml，2.7mmol）in 30ml toluene was refluxed under N_2_ for 18hrs. The reaction mixture was concentrated, the residue was dissolved in 20ml acetone , K_2_CO_3_（2.2g，16,2mmol）and (bromomethyl)benzene （3.8ml, 32.4mmol） was added and the reaction was stirred at 60℃ for overnight. Monitored by TLC, cooled to rt, TEA（3.7ml，27mmol）and EA was added to the reaction, 10%H_2_SO_4_ was added and the reaction was become clearly, the organic layer was washed with water, brine, dried and column with PE/EA=15/1 to get 500mg of white solid product, yield 33.7%.

**Note:**

**8**

**Experimental:** **PN310-31**

MeI(140µl，2.2mmol) was added to a mixture of **7** (200mg，0.73mol) and K2CO3（403mg，2.92mol）in DMF(10ml), the mixture was stirred at rt for 6hrs. Monitored by TLC, EA was added and the solution washed with water for 8 times, brine, dried and concentrated togive compound 8 180mg, yield 81.8%.

**Note:**

**9**

c

**Experimental:** **PN310-32**

Compound **8** (180mg, 0.6mol) was dissolved in 5ml methanol, NaOH (47mg，1.2mmol) was added to the reaction, water was added then NaOH was dissolved, refluxed for 2hrs. The reaction solution was concentrated, 5ml water was added and acified with 1N HCl to pH=3~4, extrated with EA for 3 times, combined the EA, washed with brine, dried with Na_2_SO_4_, concentrated to give 9 170 mg,yield 98%.

**10**

**Experimental:** **PN310-**

Compound **9**（170mg, 0.59mmol）, **4**（140mg, 0.81mmol）dissolved in DCM（10ml）, EDCI(170mg，0.88mmol), HOBt（120mg, 0.88mmol）, DIPEA（295µl，1.77mmol） was added to the solution, the stired at rt for overnight, Monitored by TLC, DCM was added and washed with water and brine, purified by Pre-TLC to giveproduct 55mg, yield 21.0%.

# 4. Spectrum

**TLC analysis:**

**^1^H NMR**

**PN310-27**

**PN310-13**

**PN310-31**

**PN310-34**
