# Supplementary material for: Discovery of Novel Hepatitis C Virus NS5B Polymerase Inhibitors by Combining Random Forest, Multiple e-Pharmacophore Modeling and Docking
Source: PLoS One. 2016 Feb 4;11(2):e0148181. doi: 10.1371/journal.pone.0148181 (PMC4742222; doi:10.1371/journal.pone.0148181)
Supplement: S1 File — (ZIP) [file pone.0148181.s005.zip › N2 Final Report.doc]

PracticaChem

**Project Report**

Code: N2

Project Leader: Na Zhang

Project member: Na Zhang

6 , 26 , 2014

# 1. Delivery Information

| Compound ID | Structure | Amount | Spectra | Delivery Date |
| --- | --- | --- | --- | --- |
| **N2** |  | 15mg | PN310-08 | 26,6,2014 |

# 2. Goal

To synthesize 15mg of compound **N2**.

**Synthetic route**

# 3. Experimental and discussion

**Experimental:** **PN310-05**

**1**（1g, 6.6mmol） was dissolved in 10%NaOH（6mL），**2**（0.6g，6.6mmol） was added and the reaction solution was heated to 90℃ for 2hrs. The reaction was cooled to rt, con. HCl was added, filtered and the solid was washed whit water, dried and recrystal with EtOH to give product. 0.17g, yield 12%。

**Experimental: PN310-08**

**3**（50mg，0.24mmol）dissolved in DMF, **4**（37uL，0.24mmol）, EDCI（55mg，0.288mmol)，HOBT（39mg，0.288mmol)，DIPEA（62mg，0.48mmol) were added to the solution, then stirred at rt for overnight. DCM was added to the solution, washed with water for 3 times, brine, dried with Na2SO4, concentrated and purified by Pre-TLC（PE/EA=6/1）15mg，yield 14.0%.

**Note:**

**TLC analysis:**

**^1^H NMR**

**LC-MS**
