# Supplementary material for: Discovery of Novel Hepatitis C Virus NS5B Polymerase Inhibitors by Combining Random Forest, Multiple e-Pharmacophore Modeling and Docking
Source: PLoS One. 2016 Feb 4;11(2):e0148181. doi: 10.1371/journal.pone.0148181 (PMC4742222; doi:10.1371/journal.pone.0148181)
Supplement: S1 File — (ZIP) [file pone.0148181.s005.zip › N3 Final Report.doc]

PracticaChem

**Project Report**

Code: **N3**

Project Leader: Jing Zhao

Project member: Jing Zhao

Jun. 26, 2014

# 1. Delivery Information

| Compound ID | Structure | Amount | Spectra | Delivery Date |
| --- | --- | --- | --- | --- |
| **N3** |  | 80mg | NMR: PN295-57 | Jun.26.2014 |

# 2. Goal

To synthesize 80mg of **N3 .**

**Synthetic route**

3. Experimental and discussion

**N3-01**

**Experimental:** **PN295-54**

To chlorosulfonic acid (20.0mL) was added Compound **1** (9.60 g , 0.1mol) in a state divided into several portions, and the mixture was stirred at 150°C for 3 hours. The reaction solution was left standing to cool to room temperature, thionyl chloride (11.0 mL) was then added dropwise, and the mixture was stirred at 100°C for 4 hours. TLC (PE:EtOAc=2:1) showed that the reaction was finished. The reaction solution was left standing to cool to room temperature, poured into iced water, and neutralized with sodium carbonate. The resultant solid was collected through filtration, washed with water, then dissolved in chloroform, and dried over anhydrous sodium sulfate, concentrated to get the compound **2** (6.04g, yield: 31.1%) as a light brown solid.

Note:

1. 滴加氯磺酸反应过程很剧烈，一定要小心且慢。

**N3-02**

**Experimental:** **PN295-55**

To a stirred solution of compound **2** (500mg, 2.57mmol) and Boc-NH_2_NH_2_(509mg,3.85mmol) in DCM(10mL), DIPEA(0.85mL, 5.14mmol) was added. The reaction mixture was stirred at room temperature overnight .. The reaction solution was cooled to room temperature. The resultant solid was collected through filtration, washed with chloroform several times, dried over and got the compound **3** (430mg, yield:57.6%) as a white solid.

Note:

1. 产物紫外灯下不显色。

**N3-03**

**Experimental:** **PN295-56**

To a stirred solution of compound **3** (150mg, 0.52mmol) in Methanol(5mL),HCl.Et_2_O(2mL) was added. The reaction mixture was stirred at room temperature about 2hours. The reaction solution was concentrated to get the compound **4** (120mg, yield:>100%) as a colorless oil .

**N3-04**

**Experimental:** **PN295-51**

A mixture solution of compound **5** (4.34mL, 45mmol) and compound **B** was heated at 100°C in an open flask for 1hour. TLC (PE:EtOAc=5:1) showed that the reaction was finished. The reaction solution was cooled to room temperature. The resultant solid(compound **6**) was collected through filtration, and used directly in next step.

**N3-05**

**Experimental:** **PN295-52**

A mixture solution of compound **6** and Dowtherm A (100mL) was refluxed for 8hours. TLC (PE:EtOAc=5:1) showed that the reaction was finished. The reaction solution was cooled to room temperature. The resultant solid was collected through filtration, washed with PE several times, dried over and got the compound **7** (1.2g) as a white solid.

**N3-06**

**Experimental:** **PN295-53**

A mixture of compound **7** (500mg, 2.12mmol) in 10% NaOH solution(10mL) was refluxed for 1 hour. TLC (DCM/MeOH =10:1) showed that the reaction was finished. The reaction solution was cooled to room temperature and acidified with 10% sulfuric acid. The precipitate was filtered off, washed with water, dried over and got the compound **8**(400mg, yield:91%) as a white solid..

**N3-06**

**Experimental:** **PN295-57**

A mixture of compound **8** (109mg, 0.53mmol), compound **4** (120mg, 0.63mmol), HATU(240mg, 0.63mmol), DIPEA(0.26mL, 1.59mmol) in DMF(5mL) was stirred at room temperature overnight. TLC (DCM/MeOH =10:1) showed that the reaction was finished. The reaction solution was concentrated, dissolved into DCM/MeOH =10:1,The precipitate was filtered off, dried over and got the **N3**(80mg, yield:40%) as a white solid..

4.TLC analysis:

5. ^1^H NMR

1,2-dimethyl-1H-imidazole-4-sulfonyl chloride

tert-butyl 2-(1,2-dimethyl-1H-imidazol-4-ylsulfonyl)hydrazinecarboxylate

Ethyl8-fluoro-4-oxo-1,4-dihydroquinoline-3-carboxylate

8-fluoro-4-oxo-1,4-dihydroquinoline-3-carboxylic acid

N-(8-fluoro-4-oxo-1,4-dihydroquinoline-3-carbonyl)-1,2-dimethyl-1H-imidazole-4-sulfonohydrazide
