# Supplementary material for: Discovery of Novel Hepatitis C Virus NS5B Polymerase Inhibitors by Combining Random Forest, Multiple e-Pharmacophore Modeling and Docking
Source: PLoS One. 2016 Feb 4;11(2):e0148181. doi: 10.1371/journal.pone.0148181 (PMC4742222; doi:10.1371/journal.pone.0148181)
Supplement: S1 File — (ZIP) [file pone.0148181.s005.zip › N4 Final Report.doc]

PracticaChem

**Project Report**

Code: N4

Project Leader: Li Wang

Project member: Xueting Wang

Aug 17, 2015

# 1. Delivery Information

| Compound ID | Structure | Amount | Spectra | Delivery Date |
| --- | --- | --- | --- | --- |
| N4 |  | 10mg | NMR | Jul，2014 |

# 2. Goal

To synthesize 10mg of N4.

# 3. Synthetic route

# 4. Experimental and discussion

**N4-01**

To a stirred solution of compound B（10.68g，97.03mmol）in 1,2-DCE (75mL) and AlCl_3_ (12.94g,97.03mmol), 1 (15g,97.03mmol) was added at 35℃ and stirred for 1 h. The reaction was poured onto ice water and adjusted PH-6 using with 1N HCl solution, And then filtered, The filtrate was poured onto water and extracted with DCM. The organic phase was washed sat brine, dried over Na_2_SO_4_ filtered, and evaporated under vacuum. The crude product was purified by silica gel chromatography and afforded the product 12g .

**N4-02**

To a stirred solution of compound 2（6.3g，27.6mmol）and K_2_CO_3_ (15.26g,110.4mmol) in DMF（50mL）, The mixture was degassed for 10 min and refilled with Ar, then was added AC_2_O（11.26g，110.4mmol）. The reaction mixture was stirred at 115℃ for 1.5 h under Ar. Diluted the reaction mixture with water and extracted with EA, finally organic layer was washed with brine, dried over Na_2_SO_4_ , filtered, and evaporated under vacuum. The crude product was washing with Et_2_O , filtered, and afforded the product 5.24g.

**N4-03**

To the solution of compound 3 (240mg, 0.951mmol) and K_2_CO_3_ (263mg, 1.903mmol) in DMF (5mL) was added C (108mg, 1.141mmol) over 0.5h under Ar. Diluted the reaction mixture with water and extracted with EA, the aqueous phase was adjusted PH-4 using with 1N NaOH solution, extracted with EA, finally organic layer was washed with brine, dried over Na_2_SO_4_ filtered, and evaporated under vacuum to get product 180mg.

**N4-04**

To a stirred solution of compound 4（175mg，0.564mmol）and B (84mg,0.677mmol) in THF（4mL）was added TEA（227mg, 2.25mmol）, EDCI (119mg, 0.621mmol), HOBT (84mg, 0.621mmol) at 0℃. The reaction mixture was stirred at room temperature overnight. Diluted the reaction mixture with water and extracted with EA, finally organic layer was washed with brine, dried over Na_2_SO_4_ filtered, and evaporated under vacuum, then through the thick lining board purification to get the product 40mg .
